# Risk prediction models for cancer therapy related cardiac dysfunction in patients with cancer and cancer survivors: systematic review and meta-analysis

**DOI:** 10.1136/bmj-2025-084062

**Published:** 2025-09-23

**Authors:** Clara Gomes, Jesse Geels, Thomas P A Debray, Arjan Malekzadeh, Folkert W Asselbergs, Marijke Linschoten

**Affiliations:** 1 Department of Cardiology, Amsterdam Cardiovascular Sciences, Amsterdam University Medical Centre, University of Amsterdam, Amsterdam, Netherlands; 2 Smart Data Analysis and Statistics, Utrecht, Netherlands; 3 Medical Library, Amsterdam University Medical Centre, University of Amsterdam, Amsterdam, Netherlands; 4 Institute of Health Informatics, University College London, London, UK; 5 National Institute for Health Research University College London Hospitals Biomedical Research Centre, University College London, London, UK; 6 Netherlands Heart Institute, Utrecht, Netherlands

## Abstract

**Objectives:**

To systematically review all prediction models developed or validated for cancer therapy related cardiac dysfunction (CTRCD) and to quantitatively analyse their performance.

**Design:**

Systematic review and meta-analysis.

**Data sources:**

Embase, Medline, and the Cochrane Central Register of Controlled Trials, from inception to 23 August 2024.

**Eligibility criteria for selecting studies:**

Studies that developed or externally validated multivariable models to predict CTRCD risk in young people (children, adolescents, and young adults (≤39 years)) or older adults (≥40 years) with cancer or cancer survivors treated with systemic antineoplastic agents. Studies on radiation induced cardiotoxicity were excluded.

**Methods:**

Two reviewers independently screened studies, extracted data, and assessed risk of bias using the Prediction model Risk Of Bias ASsessment Tool. Performance measures were pooled using random effects meta-analyses.

**Results:**

56 studies were included, reporting 51 developed models and 12 externally validated models. Most models were developed in adults (n=34/51, 67%), primarily for breast cancer (n=20/34, 59%) or haematological malignancies (n=6/34, 18%) to determine pretreatment risk (n=33/34, 97%). In young people, most developed models (n=16/17, 94%) focused on long term risk assessment, mostly in survivors of haematological malignancies. Discrimination and calibration metrics were reported for 13/51 (25%) developed models and 6/44 (14%) external validations. Nearly all models were at high risk of bias. 12/51 (24%) developed models underwent external validation, four of 17 (24%) in young people and eight of 34 (24%) in adult populations. The Heart Failure Association-International Cardio-Oncology Society (HFA-ICOS) tool was the most frequently validated (11 times), mainly in patients with breast cancer receiving HER2 (human epidermal growth factor receptor 2) targeted therapies (5/11, 45%). Across all external validations, this tool consistently underestimated risk, with observed event rates exceeding predicted risks, especially in studies where mild CTRCD was the most frequently reported outcome. Among patients with breast cancer treated with anti-HER2 agents, the pooled C statistic was 0.60 (95% confidence interval 0.52 to 0.68). In this population, observed pooled event rates were 12% in the low risk group (<2% predicted), 15% in the medium risk group (2-9%), 25% in the high risk group (10-19%), and 41% in the very high risk group (≥20%).

**Conclusions:**

Existing prediction models for CTRCD need additional evidence before widespread clinical adoption. Poor reporting of key performance metrics and limited external validation studies currently restrict their thorough evaluation. The HFA-ICOS tool shows suboptimal performance, especially when mild forms of CTRCD are included as events. Future research should prioritise validating and updating existing models using large, clustered datasets across various malignancies to enhance the assessment of their performance, generalisability, and clinical utility in routine practice.

**Systematic review registration:**

PROSPERO CRD42023475469.

## Introduction

Cancer therapy related cardiac dysfunction (CTRCD) is a serious complication of cancer treatments. Although anthracyclines and the monoclonal antibody trastuzumab are well known for their association with this toxicity,^1^ ^2^ risk also extends to other cytostatic agents, immunotherapies, and molecular targeted therapies.^3^ ^4^ ^5^ ^6^ ^7^ The spectrum of CTRCD encompasses a range of manifestations, which in the earliest stages may be reflected by increased levels of cardiac biomarkers (eg, troponin and brain natriuretic peptide (BNP)) or a reduction in global longitudinal strain, progressing to asymptomatic left ventricular systolic dysfunction and potentially culminating in symptomatic heart failure.^7^ CTRCD can have profound consequences for patients with cancer and cancer survivors, limiting tolerability to cancer treatment and negatively impacting quality of life and survival.^8^ Given the widespread use of antineoplastic agents associated with CTRCD, accurate prediction models are essential to enable targeted screening and monitoring, facilitating earlier diagnosis, timely treatment, and the initiation of preventive measures.

The first clinical cardio-oncology guideline released by the European Society of Cardiology (ESC) in 2022 recommends the use of the Heart Failure Association-International Cardio-Oncology Society (HFA-ICOS) risk assessment tool for patients scheduled to undergo potentially cardiotoxic antineoplastic treatments.^7^ This tool was, however, based on expert opinion,^9^ and its prognostic value and clinical utility across different settings remain unclear. Multiple other recommendations have been written, including the 2016 American Society of Clinical Oncology guideline for the prevention and monitoring of cardiac dysfunction in adults with cancer,^10^ and the 2020 European Society of Medical Oncology consensus recommendations on the management of cardiac disease in patients with cancer.^11^ However, these guidelines merely provide a list of patient and treatment related factors that should be taken into consideration to identify patients at increased risk for developing CTRCD, rather than a risk prediction model.

Although several CTRCD risk prediction models have been developed for patients with various malignancies,^12^ ^13^ ^14^ ^15^ none has been thoroughly evaluated. In this systematic review, we comprehensively evaluated existing CTRCD risk prediction models and related external validation studies and assessed the performance and risk of bias of the models to determine their reliability to guide personalised screening and monitoring of CTRCD in clinical practice.

## Methods

This systematic review is reported according to the Transparent Reporting of multivariable prediction models for Individual Prognosis or Diagnosis for Systematic Reviews and Meta-Analyses (TRIPOD-SRMA) guideline^16^ (see supplementary table 1) and the reporting guidelines of the Preferred Reporting Items for Systematic reviews and Meta-Analyses (PRISMA) (see supplementary table 2), including the extensions for abstracts and literature searches (PRISMA-S) (see supplementary tables 3 and 4).^17^ ^18^ The protocol is available online. The protocol underwent some amendments. At the time of protocol registration, we planned to include only models with at least three predictors. During the screening phase, we opted to track models with only two predictors and ultimately included them if they met all other eligibility criteria. Similarly, although the original protocol specified the exclusion of non-English studies, we reviewed these using online translation tools. Of the seven non-English articles identified, none met all inclusion criteria; therefore, no studies were excluded solely based on language.

### Search strategy

We conducted a systematic literature search in Medline (Ovid), Embase (Ovid), and the Cochrane Central Register of Controlled Trials to identify relevant studies published from inception of each database to 21 September 2023. We repeated the search with the original search string on 23 August 2024. The search string was generated together with a medical librarian (AM), after establishing a comprehensive set of keywords and subject headings pertaining to cancer and CTRCD (see supplementary table 5). Search filters originally developed and validated to identify prognostic studies using the PubMed interface^19^ ^20^ ^21^ were adapted for use within the Ovid interface. The search was restricted to studies in humans and specific publication types, excluding case reports, reviews, editorials, and letters. No restrictions were applied based on language or publication date. Duplicates were removed using an online tool developed in-house (DedupEndNote). To identify further relevant studies, we screened reference lists of selected studies, reviews on the topic, and clinical guidelines.

### Study selection and eligibility criteria

Two researchers (CG and ML) manually and independently screened study titles and abstracts for eligibility, resolving any discrepancies through discussion. Full text articles were assessed for relevance, with disagreements resolved through consultation with a third researcher (TPAD). Non-English studies were translated using online translation software.

Studies were eligible if they met the following criteria: reported the development, validation, or update of one or multiple prediction models to estimate CTRCD risk in patients with cancer or cancer survivors; the outcome of interest encompassed any form of CTRCD as described in the ESC cardio-oncology guideline, including symptomatic heart failure, any new decline in global longitudinal strain or left ventricular ejection fraction, or new increase in cardiac biomarkers (troponins or BNP)^7^; CTRCD was either the primary outcome or part of a composite cardiovascular outcome; the study population was primarily composed of patients treated with chemotherapy or targeted treatments, such as protein kinase inhibitors, monoclonal antibodies, or immunotherapy; the intended time of use of the model was either before the initiation of systemic anticancer treatment or in cancer survivors; and the prediction model included at least two predictors. External validation studies were included if they met the listed criteria, regardless of whether the prediction model was originally developed specifically for patients with cancer or for another population (eg, models developed to predict cardiovascular risk in individuals without cancer). Models based on expert opinion, rather than a formal development process, were also considered eligible if externally validated.

We excluded studies that primarily focused on radiation induced cardiotoxicity; evaluated the predictive or incremental value of individual predictors; had a sample size of <100 participants; developed models that relied on the collection of longitudinal data during cancer treatment (eg, changes in left ventricular ejection fraction from baseline) and therefore could not be applied at the initiation of cancer treatment; used a non-nested case-control or cross sectional design; or were solely published on preprint servers.

### Data extraction and risk of bias assessment

We used the CHecklist for critical Appraisal and data extraction for systematic Reviews of prediction Modelling Studies (CHARMS) to extract data from selected studies,^22^ supplemented by items extracted during a previous systematic review on cardiovascular risk prediction models.^23^

For studies describing the development of a prognostic model, we extracted data on study design, study population and sample size, outcome definitions, prediction horizon, duration of follow-up, number and type of predictors incorporated into the model, number of outcome events, modelling method, method of internal validation, performance measures (eg, C statistic, calibration, or overall performance), and model presentation. For data extraction of external validation studies, we utilised a different extraction sheet, including information on the type of external validation performed, whether the validation was conducted by the same investigators who originally developed the model, if the model was adjusted or updated, and performance measures before and after any adjustments or updates. When a study reported validations in multiple independent cohorts, we counted each as a separate external validation. When both the C statistic and the area under the receiver operating characteristic curve were reported, we extracted the C statistic—throughout this study both are referred to as C statistic for consistency.

Outcome definitions were extracted as reported in each study. For each outcome of interest, we collected all available performance metrics. For instance, if a study reported a composite cardiovascular outcome and heart failure separately, we extracted performance measures on both. Similarly, if performance was assessed at multiple time points, all were recorded. If multiple models were developed within a single study, we extracted data for all the models unless the authors explicitly identified one as the model intended for clinical use. In studies that compared multiple machine learning techniques to identify the best performing approach, we only extracted data from the model with the best performance. Two researchers (CG and JG) independently extracted data. Any disagreements were resolved through discussion and by consulting a third researcher (ML).

The Prediction model Risk Of Bias ASsessment Tool (PROBAST) was used to evaluate the risk of bias of retrieved studies,^24^ according to the four key domains: participants, predictors, outcome, and analysis. Two researchers (CG and JG) independently conducted the assessments in duplicate, utilising a standardised quality assessment spreadsheet. Each potential source of bias was graded as low, high, or unclear, with a justification provided for each judgement. We evaluated the applicability of studies in relation to the target population defined in the research question of this systematic review. Any disagreements were resolved through discussion, with input from a third researcher (ML) when necessary. We did not assess small study effects or publication bias using funnel plots or statistical tests, as only a few models were validated more than once, and traditional methods are underpowered with the inclusion of fewer than 10 studies.

### Data synthesis and statistical analysis

For descriptive purposes, we categorised the included studies based on the age group of the population in which the model was developed or validated: children together with adolescents and young adults (≤39 years) versus older adults (≥40 years). Predictors were combined into related categories (eg, body mass index grouped together with obesity), as displayed in supplementary table 6, and plotted to provide an overview of the most included predictors across all developed models. Likewise, outcome definitions were grouped as either CTRCD, including heart failure, decline in left ventricular ejection fraction or global longitudinal strain, or increase in cardiac biomarkers; or composite cardiovascular outcomes, including other cardiovascular events beyond CTRCD (see supplementary table 7).

Continuous variables were reported as median and interquartile range (IQR) or mean and standard deviation, depending on data distribution. Categorical variables were summarised using absolute counts and percentages. Given the infrequent reporting of calibration measures, we compared the observed outcome proportions from external validation studies of the most validated risk assessment tool (HFA-ICOS) with the expected risk for each risk stratum according to this tool. Confidence intervals (CIs) for proportions were estimated using Wilson’s method.^25^ Potential sources of heterogeneity in the performance of this tool were assessed by categorising studies based on the severity of the most frequently reported CTRCD events, as defined by the 2022 ESC cardio-oncology guidelines^7^: studies predominantly reporting mild CTRCD, and studies in which moderate to severe (or symptomatic) CTRCD predominated (see supplementary table 8). From this analysis we excluded those studies with a composite endpoint including other cardiovascular events beyond CTRCD.

Meta-analyses were conducted to assess the performance of the HFA-ICOS tool, with a focus on the C statistic and observed outcome proportions across different risk strata. A pooled C statistic represents a weighted average of this measure across multiple external validation studies, providing an overall estimate of the model’s discrimination across different studies. Observed outcome proportions were also pooled for each risk stratum and compared with expected risks, offering an indirect assessment of calibration. Before pooling, both measures were transformed to the logit scale. For the logit transformed C statistic, we calculated standard errors from reported CIs, when available. If CIs were not reported, we derived standard errors from the reported sample sizes and observed number of events, following established methods.^26^ ^27^ ^28^

Random effects meta-analyses were performed using the restricted maximum likelihood estimator for between study variance and the Hartung-Knapp-Sidik-Jonkman adjustment, which accounts for heterogeneity when estimating pooled performance measures and CIs.^28^ ^29^ As a sensitivity analysis, we excluded HFA-ICOS external validation studies with high applicability concerns to our research question (according to PROBAST criteria) and pooled C statistics and observed outcome proportions only from studies with low concerns to assess the robustness of our findings. All analyses were conducted in R (version 4.3.2), utilising the metafor,^30^ metamisc,^31^ and meta^32^ packages.

### Patient and public involvement

No patients or members of the public were involved in this study, as it was based solely on secondary analysis of data from previously published studies.

## Results

**Fig 1:**
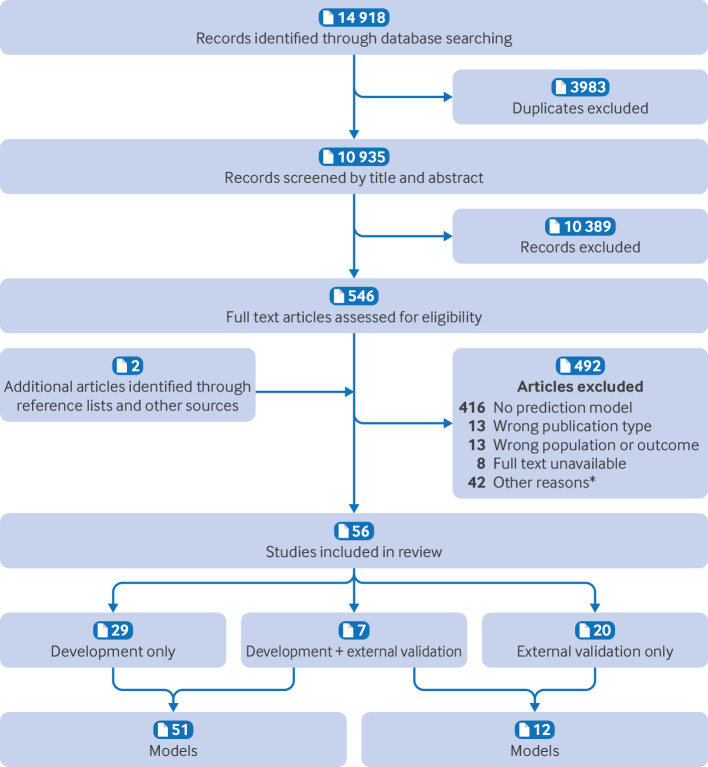
Flow diagram of study selection. *Supplementary table 9 provides a detailed account of the reasons for exclusion of these studies, which either presented a prediction model or conducted multivariable modelling but did not fully meet the inclusion criteria

Out of 10 935 articles screened by title and abstract, 546 were selected for full text assessment. Among the 546 studies, 42 involved multivariable modelling but were excluded after discussion for not meeting inclusion criteria, with most classified as prognostic factor studies (see supplementary table 9). Ultimately, 54 studies were included, supplemented by two additional studies identified through snowballing—which were not found in our initial search, bringing the total to 56 studies. Among these, 29 studies developed one or more prediction models, 20 conducted external validation, and seven performed both (fig 1). Supplementary table 10 presents a detailed overview of each study included in this systematic review.

**Fig 2:**
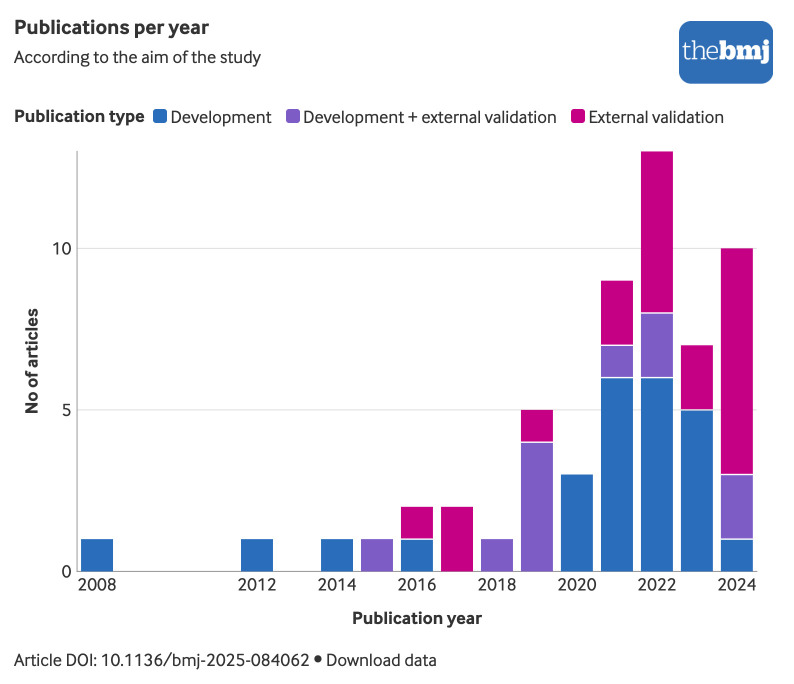
Publications per year according to aim of study (development, external validation or both). An interactive version of this graphic is available at https://public.flourish.studio/visualisation/24729343/

The number of studies developing or validating CTRCD prediction models has grown substantially in recent years, with a particular increase in those focusing on external validation (fig 2). In total, 63 prediction models were identified: 51 were developed to predict CTRCD risk in patients with cancer or cancer survivors, nine were originally developed for patients without cancer (n=8) or to predict a non-cardiovascular outcome (n=1), and three were derived from expert opinion rather than a formal model development process. Supplementary table 11 provides a detailed summary of the models that were identified through external validation studies, but for which the original development study did not fulfil the inclusion criteria of this systematic review.

### Studies on CTRCD risk prediction model development

#### Study design and participants

A total of 51 prediction models were identified, of which 67% (34/51) were developed in data derived from adults. Most models originated from cohort studies (45/51, 88%), primarily conducted in North America and Europe. Table 1 shows the key characteristics of the studies.

**Table 1:** Characteristics of included studies stratified by age category for developed models and external validations. Values are number (percentage) unless stated otherwise

Characteristics	Developed models (n=51)			External validations (n=44)	
	Adults (n=34)	Young people* (n=17)		Adults (n=36)	Young people* (n=8)
**Study design**					
Cohort	31 (91)	14 (82)		34 (94)	6 (75)
Randomised trial	3 (9)	(-)		1 (3)	2 (25)
Nested case-control/case-cohort	(-)	3 (18)		1 (3)	(-)
**Geographical location**					
North America	14 (41)	13 (76)		13 (36)	4 (50)
Europe	11 (32)	3 (18)		20 (56)	4 (50)
Asia	8 (24)	1 (6)		3 (8)	(-)
Intercontinental	1 (3)	(-)		(-)	(-)
**Median age at cancer diagnosis (years)**					
<15	(-)	15 (88)		(-)	8 (100)
30-40	(-)	2 (12)		(-)	(-)
40-50	4 (12)	(-)		3 (8)	(-)
50-60	21 (62)	(-)		25 (69)	(-)
60-80	8 (24)	(-)		7 (19)	(-)
Not reported	1 (3)	(-)		1 (3)	(-)
**Main malignancy**					
Combined childhood cancers†	(-)	14 (82)		(-)	8 (100)
Breast	20 (59)	(-)		18 (50)	(-)
Haematological malignancies‡	6 (18)	3 (18)		4 (11)	(-)
Other cancers in adults§	7 (21)	(-)		11 (31)	(-)
Not reported	1 (3)	(-)		3 (8)	(-)
**Main systemic treatment**					
Anthracycline based chemotherapy	15 (44)	8 (47)		9 (25)	1 (12)
Other chemotherapy¶	5 (15)	9 (53)		4 (11)	7 (87)
HER2 targeted therapies**	4 (12)	(-)		10 (28)	(-)
Other targeted therapies††	6 (18)	(-)		5 (14)	(-)
Unspecified	4 (12)	(-)		8 (22)	(-)
**Prediction timepoint**					
Before cancer treatment	33 (97)	1 (6)		35 (97)	(-)
Survivors (≥1 year from last treatment)	1 (3)	16 (94)		1 (3)	8 (100)
**Median follow-up (years)**					
<1	8 (24)	1 (6)		6 (17)	(-)
1-5	21 (62)	3 (18)		24 (67)	(-)
>5	5 (15)	13 (76)		4 (11)	8 (100)
Not reported	(-)	(-)		2 (6)	(-)
**Outcome definition**					
Composite	14 (41)	(-)		22 (61)	(-)
CTRCD	20 (59)	17 (100)		14 (39)	8 (100)
**Outcome handling**					
Binary	19 (56)	7 (41)		15 (42)	(-)
Time to event	15 (44)	10 (59)		21 (58)	8 (100)
**Modelling method‡‡**					
Logistic regression	16 (47)	1 (6)			
Survival analysis:					
Cox regression	9 (26)	4 (24)			
Other survival methods	4 (12)	6 (35)			
Other methods	5 (15)	6 (35)			
**Internal validation method**					
Random split sample	12 (35)	5 (29)			
Bootstrapping	10 (29)	6 (35)			
Cross validation	4 (12)	6 (35)			
None	8 (24)	(-)			
**Summary metrics, median (IQR) **					
Sample size	420 (225-1377)	1433 (796-13 060)		629 (207-1593)	1362 (364-1445)
Event rate	11.4 (7.1-19.5)	7.1 (2.2-9.6)		14.5 (8.6-24.0)	1.9 (1.7-5.7)
No of predictors	7 (5-10)	8 (7-11)		12 (7-20)	6 (4-8)
EPV ratio	10.3 (4.9-22.5)	16.0 (5.9-20.4)		7.2 (3.2-23.9)	4.0 (2.8-6.1)

CTRCD=cancer therapy related cardiac dysfunction; EPV=event per variable; HER2=human epidermal growth factor receptor 2; IQR=interquartile range; (-)=equal to zero.

* Children, adolescents, and young adults (≤39 years).

† Including haematological malignancies (eg, acute leukaemias and lymphomas), brain tumours, neuroblastoma, nephroblastoma, soft tissue sarcomas, bone tumours, and germ cell tumours.

‡ Haematopoietic cell transplantation survivors (n=2); patients with multiple myeloma (n=2), Hodgkin lymphoma (n=2), acute leukaemia (n=2), diffuse large B cell lymphoma (n=1), chronic myeloid leukaemia (n=1); and combinations of these malignancies (n=3).

§ Combination of different adult related cancers (eg, breast cancer, haematological malignancies, gastrointestinal cancer), and other cancers: lung cancer (n=2), colorectal cancer (n=1), and hepatocellular cancer (n=1).

¶ Patients treated with chemotherapy, when anthracyclines are not the primary treatment. Other specified agents: antimetabolites, fluoropyrimidines, alkylating agents, and anti-microtubule agents.

** With or without anthracyclines.

†† Proteasome inhibitors (carfilzomib), protein kinase inhibitors (sorafenib), immunomodulatory drugs, and monoclonal antibodies (nivolumab, pembrolizumab).

‡‡ Other survival methods include Weibull regression, cause specific hazard models, subdistribution hazard models, competing risk regression, and random survival forest. Other methods include artificial neural networks, random forest, and XGBoost.

Among the models developed in adults, breast cancer was the most common malignancy (20/34, 59%), followed by haematological malignancies (6/34, 18%). In contrast, for models developed in children, adolescents, and young adults, the study population often comprised cancer survivors (16/17, 94%) with a diverse range of cancers (14/17, 82%), including haematological malignancies and embryonal tumours (table 1). In paediatric populations, a few well established cohorts were leveraged to develop multiple prediction models. The Childhood Cancer Survivor Study cohort served as the basis for seven CTRCD prediction models developed by two independent research groups. Four of these models were tailored to different age categories,^33^ while the remaining three varied in their approach to account for use of anthracycline and radiation (see supplementary table 10).^34^

Although prediction horizons were seldom reported, follow-up periods were generally longer in children, adolescents, and young adults (>5 years’ duration), reflecting the intended use of the model in cancer survivors. In contrast, models in adults were typically developed to be applied before cancer treatment, and patients were followed-up during shorter periods (<5 years) (table 1). Anthracycline based chemotherapy was the mainstay of treatment in 44% (15/34) of models developed in adults and in 47% (8/17) of those developed in children, adolescents, and young adults.

#### Outcomes and modelling methods

Among children, adolescents, and young adults, all models were developed to predict heart failure and other CTRCD surrogates, such as a new increase in cardiac biomarkers and/or a new decline in global longitudinal strain or left ventricular ejection fraction. In adults, a substantial proportion of models (14/34, 41%) were developed to predict a composite outcome, including other cardiovascular events besides CTRCD (see supplementary table 7). The outcome was assessed as a binary variable in 56% (19/34) of models developed in adults and in 41% (7/17) of models developed in children, adolescents, and young adults, without accounting for censoring or competing risks such as cancer related mortality. Logistic regression and survival analysis were used in the development of 78% (40/51) of models (table 1).

#### Predictors

**Fig 3:**
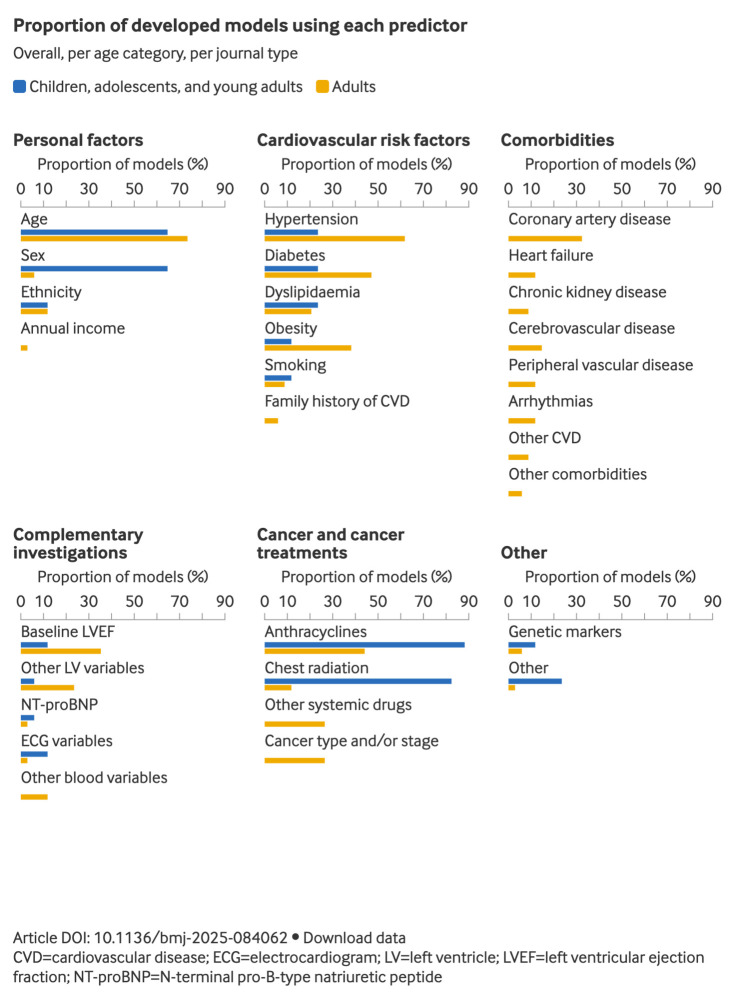
Percentage of developed models using each predictor, by age group. An interactive version of this graphic is available at https://public.flourish.studio/story/3303126/

Developed models included a median of 7 (IQR 5-11) predictors. In children, adolescents, and young adults, 65% (11/17) of developed models included age, sex, anthracycline use and/or dose, and radiation therapy (fig 3). Among models developed for adults, the five most included predictors were age (25/34, 74%), hypertension (21/34, 62%), diabetes (16/34, 47%), use of anthracyclines (15/34, 44%), and chest radiation (13/34, 38%). Supplementary table 12 provides an overview of the main predictors included in each model, grouped into broad domains (eg, demographics, comorbidities, cancer treatment). To illustrate how model complexity relates to discrimination, the corresponding C statistics are also provided.

Models published in cardiology journals (19/51, 37%) often incorporated cardiovascular comorbidities, cardiac imaging variables (eg, baseline left ventricular ejection fraction), and cardiac serum biomarkers, in contrast with models published in oncology journals (23/51, 45%). Figure 3 shows the most included predictors in models published in oncology versus cardiology journals.

#### Internal validation and reporting of performance measures

A total of 43 models (43/51, 84%) underwent internal validation, with the most employed internal validation methods listed in table 1 and specified for each model in supplementary table 13. The C statistic was the most frequently reported performance metric, available for 94% (48/51) of models. Calibration measures were reported for 27% (14/51) of the developed models, while both calibration and discrimination measures were reported for 25% (13/51) of models (table 2). Supplementary table 13 outlines all reported performance measures.

**Table 2:** Reporting of performance measures for developed models (n=51) and models that were externally validated (n=24)

Performance measures	No (%) of developed models (n=51)	No (%) of external validations (n=44)
Discrimination measures:		
Any discrimination measure	48 (94)	37 (84)
C statistic	48 (94)	34 (77)
Other measures*	15 (29)	8 (18)
Calibration measures:		
Any calibration measure	14 (27)	11 (25)
Calibration plot	10 (20)	11 (25)
Calibration slope and intercept	1 (2)	0 (0)
Observed:expected	2 (4)	0 (0)
Hosmer-Lemeshow test	5 (10)	0 (0)
Overall performance measures:		
Brier score	2 (4)	1 (2)
Other performance measures†	4 (8)	1 (2)
Any performance measure	49 (96)	37 (84)
Both calibration and discrimination	13 (25)	6 (14)

* Includes sensitivity, specificity, positive predictive value, and negative predictive value.

† Includes precision, recall, F1 score, misclassification rate, false negative rate, and false positive rate.

#### Model presentation

Models were presented as a sum score (n=21), online calculator (n=9), full regression formula, including intercept and coefficients (n=5), nomogram (n=3), and risk score table (n=1). Additionally, three models presented predictor coefficients but lacked an intercept, while 17 out of 51 models (33%) presented neither regression coefficients nor intercepts (see supplementary table 10).

### Studies on external validation of a CTRCD risk prediction model

Twenty seven studies reported 44 external validations of 24 models. Among these, 12 models were either originally developed in populations without cancer (n=8), aimed at predicting non-cardiovascular outcomes (eg, cancer mortality (n=1)), or based on expert opinion without formal development methodology (n=3). Notably, 13 external validations were conducted either in the same study as the model was developed or by the same research group, while seven validations (spanning five models) were performed independently by separate research groups.

Among adults, the HFA-ICOS risk assessment tool was the most frequently validated, with 11 external validations. However, the tool is composed of six different models depending on the type of treatment administered (anthracyclines, HER2 (human epidermal growth factor receptor 2) targeted therapies, vascular endothelial growth factor (VEGF) inhibitors, BCR-ABL (Breakpoint cluster region Abelson murine leukaemia viral oncogene homolog 1) inhibitors, multiple myeloma therapies, and RAF (rapidly accelerated fibrosarcoma) and MEK (mitogen activated protein kinase) inhibitors). Most external validations (n=5) were conducted in patients with breast cancer receiving HER2 targeted therapies, followed by patients with breast cancer receiving anthracyclines (n=3). Table 3 outlines the main characteristics of the HFA-ICOS external validation studies. Among the other models externally validated in adults, one model was validated three times, four were validated twice, and the remaining 16 were validated once (see supplementary table 13). In children, adolescents, and young adults, two models were validated three times and two were validated once, all among survivors of childhood cancer for ≥5 years.

**Table 3:** Studies that conducted external validation of the HFA-ICOS risk assessment tool grouped by class of agents

Reference	Cancer	Treatment	Design	Location	Outcome definition	Follow-up*	Sample	Events	C statistic	Calibration†	Overall applicability	Overall risk of bias
**HER2 targeted therapies**												
Battisti 2021^35^	Breast	Trastuzumab+/-pertuzumab/anthracyclines	Cohort	UK, single centre	Cardiovascular death; LVEF decline; heart failure; trastuzumab discontinuation due to cardiotoxicity	<1 year	931	155	0.56	NR	Low	High
Cronin 2023^36^	Breast	Trastuzumab+/-anthracyclines	Cohort	Ireland, multicentre	Cardiovascular death; LVEF decline; heart failure; trastuzumab discontinuation due to cardiotoxicity	>5 years	507	23	0.64	NR	Low	High
Liu 2022^37^	Breast	Trastuzumab+/-anthracyclines	Cohort	China, single centre	LVEF decline	336 days	212	72	0.67	NR	High	High
Suntheralingam 2022^38^	Breast	Trastuzumab+anthracyclines (90%)	Cohort	Canada, single centre	LVEF decline	~1.5 years	629	151	0.58	Poor	Low	High
Tini 2022^39^	Breast	Trastuzumab+/-Pertuzumab	Cohort	Italy, multicentre	Left ventricular dysfunction/any cardiovascular event‡	2.6 years (mean)	171	21	NR	NR	High	High
**Anthracyclines**												
Di Lisi 2024^40^	Breast	Anthracyclines+trastuzumab (18%)	Cohort	Italy, multicentre	LVEF/GLS decline	NR	109	26	NR	NR	Unclear	High
Rivero-Santana 2024^41^	Breast 64%, NHL 17%, other	Anthracyclines+trastuzumab (17%)	Registry	Spain, multicentre	Symptomatic CTRCD; moderate-severe asymptomatic CTRCD	54.8 months	1066	69	0.78	Good	Low	High
Tini 2022^39^	Breast	Anthracyclines	Cohort	Italy, multicentre	Left ventricular dysfunction/any cardiovascular event‡	2.4 years (mean)	202	4	NR	NR	High	High
**BCR-ABL inhibitors**												
Fernando 2024^42^	CML	Nilotinib	Cohort	UK, single centre	Any cardiovascular event§	62.9 months	229	48	0.68	NR	High	High
**VEGF inhibitors**												
Stefanini 2024^43^	Hepatocellular carcinoma	Sorafenib	Registry	Italy, multicentre	Major cardiovascular events¶	11.3 months	843	34	0.654	NR	High	High
**Unspecified**												
Shibata 2022^44^	Haematological malignancies 82%, breast 18%	Multiple treatment classes	Cohort	Japan, single centre	Any cardiovascular event**	716 days	486	97	NR	NR	High	High

BCR-ABL=breakpoint cluster region Abelson murine leukaemia viral oncogene homolog 1; CML=chronic myeloid leukaemia; CTRCD=cancer therapy related cardiac dysfunction; GLS=global longitudinal strain; HER2=human epidermal growth factor receptor 2; HFA-ICOS=Heart Failure Association-International Cardio-Oncology Society; LVEF=left ventricular ejection fraction; NHL=non-Hodgkin lymphoma; NR=not reported; VEGF=vascular endothelial growth factor.

* Median by default, mean when specified.

† Calibration as assessed by authors of the study.

‡ Including cardiac tamponade, stroke, ectopic ventricular beats, uncontrolled arterial hypertension, heart failure with preserved ejection fraction, and pulmonary embolism.

§ Including acute coronary syndrome, acute peripheral vascular disease, hypertension, arrhythmias, cerebrovascular accidents, heart failure, syncope, and venous thromboembolism.

¶ Including heart failure, acute coronary syndrome, cerebrovascular accidents, and peripheral ischaemia.

** Including heart failure, left ventricular dysfunction, acute coronary syndrome, venous thromboembolism, new arterial hypertension, atrial fibrillation, bradycardia, QT prolongation, and pericardial effusion.

Overall, six external validations (6/44, 14%) reported both calibration and discrimination measures, while seven (7/44, 16%) reported no performance metrics (table 2). Studies that lacked performance measures primarily described the distribution of patients across risk strata and how these categorisations aligned with observed outcomes. Supplementary figure 1 shows the distribution of C statistics across development datasets (with or without internal validation) and external validation samples, and supplementary table 13 provides further details.

#### Quality assessment

**Fig 4:**
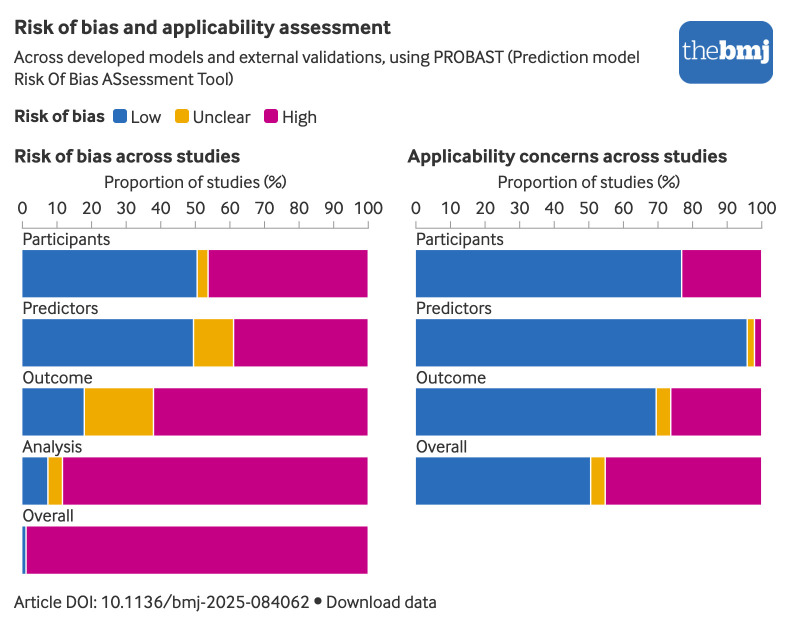
Risk of bias and applicability assessment according to the Prediction model Risk Of Bias ASsessment Tool (PROBAST). An interactive version of this graphic is available at https://public.flourish.studio/visualisation/24730549/

Supplementary tables 14 and 15 provide the assessment of risk of bias and applicability for each model’s development and external validation for each PROBAST domain and for each signalling question, respectively. The overall risk of bias was deemed high for all developed models (n=51) and for 98% (43/44) of external validations, primarily due to concerns in the analysis domain (fig 4). Key factors included inadequate reporting of performance measures (75/95, 79%), poor handling of missing data (72/95, 76%), failure to account for censoring and competing risks (61/95, 64%), and lack of strategies to prevent overfitting (31/51, 61%). Other concerns were related to the handling of categorical and continuous predictors (54/95, 57%), selection of predictors based on univariable analysis (23/51, 45%), and small sample sizes (29/95, 31%). Additionally, 33% (17/51) of developed models and 59% (26/44) of external validations raised concerns about applicability, predominantly in the participants and outcome domains.

### Quantitative analysis

#### HFA-ICOS risk assessment tool

**Fig 5:**
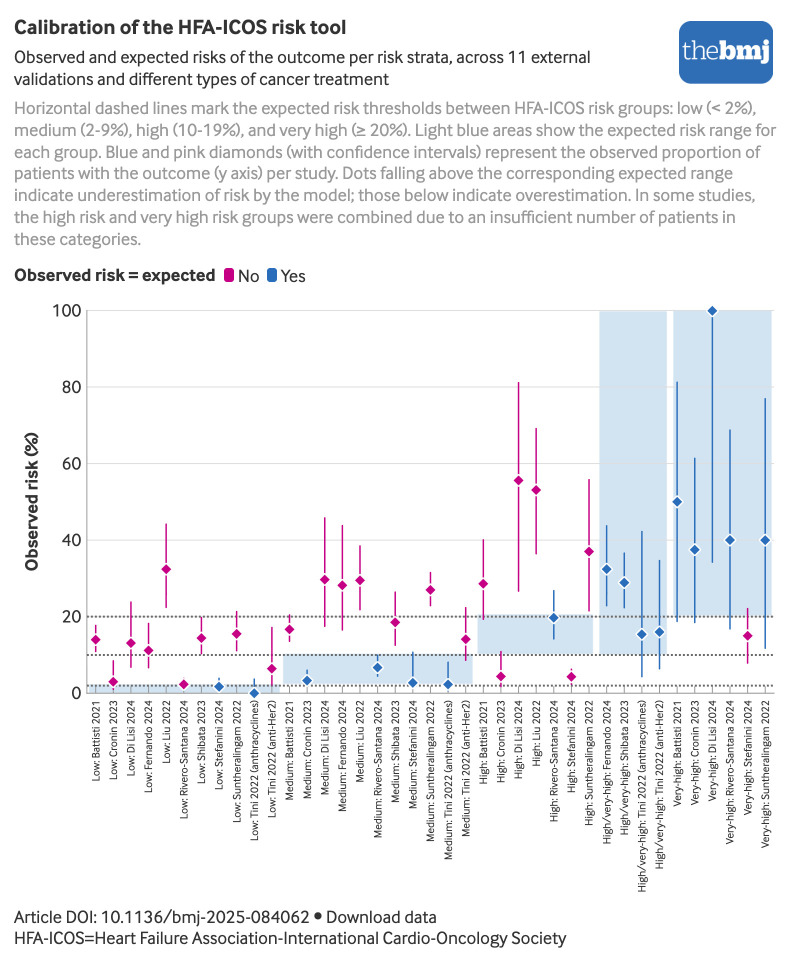
Calibration of HFA-ICOS risk tool across 11 external validation studies. Horizontal dashed lines mark the expected risk thresholds between HFA-ICOS risk group^9^: low (<2%), medium (2-9%), high (10-19%), and very high (≥20%). Light blue areas show the expected risk range for each group. Blue and pink diamonds (with confidence intervals) represent the observed proportion of patients with the outcome (y axis) per study. Diamonds above the corresponding expected range indicate underestimation of risk by the model; those below indicate overestimation. In some studies, the high risk and very high risk groups were combined owing to an insufficient number of patients in these categories. HFA-ICOS=Heart Failure Association-International Cardio-Oncology Society. An interactive version of this graphic is available at https://public.flourish.studio/visualisation/24744092/

Figure 5 shows the observed event proportions by HFA-ICOS risk category across all validation studies, compared with the expected risk ranges.^9^ In most studies the incidence of CTRCD and other cardiovascular events in patients classified as low or medium risk exceeded the 10% threshold typically associated with high risk, indicating that this tool underestimates the actual risk. Similarly, among high risk patients, event incidences exceeded 20%, a level more consistent with very high risk classifications according to this tool. Notably, when studies focusing on CTRCD related events were categorised based on the severity of the predominantly reported events (ie, mild versus moderate to severe CTRCD) (see supplementary table 8), the observed outcome proportions more closely aligned with the expected risks in the studies focusing on more severe events (see supplementary figure 2).

**Fig 6:**
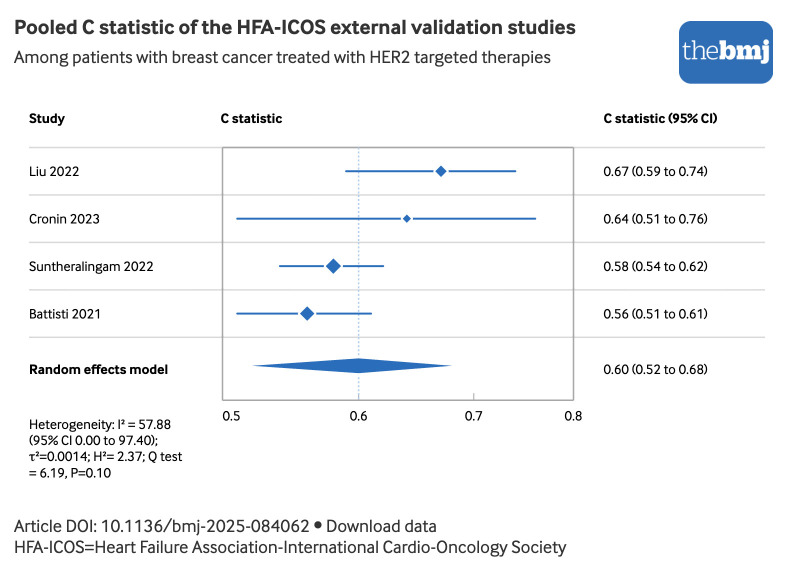
Forest plot of the C statistic of studies that externally validated the HFA-ICOS risk assessment tool in patients with breast cancer receiving HER2 (human epidermal growth factor receptor 2) targeted therapies. External validation studies of the HFA-ICOS tool in patients receiving other cancer therapies were excluded, as they correspond to different underlying models. An interactive version of this graphic is available at https://public.flourish.studio/visualisation/24743196/

The meta-analysis was restricted to studies validating the model in patients treated with HER2 targeted therapies (n=5), given the limited number of external validation studies for the models of other classes of drugs (ie, anthracyclines, VEGF inhibitors and BCR-ABL inhibitors) (table 3). The pooled observed risks were 12% (low risk group), 15% (medium risk), 25% (high risk), and 41% (very high risk), exceeding the expected risks by <2%, 2-9%, 10-19%, and ≥20%, respectively (see supplementary figure 3).^9^ Importantly, three of these studies reported mild CTRCD as the predominant outcome. The pooled C statistic, based on the four studies that reported this metric, was 0.60 (95% CI 0.52 to 0.68) (fig 6) with moderate heterogeneity (I^2^=57.88% (95% CI 0.00 to 97.40), τ^2^=0.0014, H^2^=2.37). Results of a sensitivity analysis limited to studies with low concerns about applicability were similar (see supplementary figures 4 and 5).

## Discussion

This systematic review provides a comprehensive summary and quantitative analysis of CTRCD risk prediction models developed for adults and for young people (children, adolescents, and young adults) with cancer and cancer survivors treated with systemic antineoplastic agents. The key findings were: most studies were deemed at high risk of bias; most prediction models were developed in data derived from patients with breast cancer, and other cancer populations were underrepresented in both model development and validation studies; the limited number of external validation studies, combined with substantial heterogeneity between studies and poor reporting of performance measures, hindered thorough assessment of the performance of existing prediction models; the HFA-ICOS risk assessment tool was the most validated, particularly in patients with breast cancer treated with HER2 targeted therapies, but its discriminative performance was suboptimal in this population, with a pooled C statistic of 0.60 (95% CI 0.52 to 0.68); and overall the HFA-ICOS risk assessment tool tended to systematically underestimate the risk of CTRCD, particularly in studies where mild CTRCD events comprised the majority of reported outcomes.

Although a substantial number of CTRCD risk prediction models exist, evidence supporting their clinical use remains limited. We assessed all developed models and 98% (43/44) of external validations as being at high risk of bias, primarily due to methodological concerns within the analysis domain (see supplementary table 14). These findings align with previous systematic reviews of risk prediction models, which have highlighted pervasive methodological shortcomings and poor reporting standards across various clinical domains.^45^ ^46^ ^47^

Despite the growing body of research on CTRCD risk stratification, many cancer populations remain underrepresented in both model development and validation. No studies have been conducted in Africa, Australia, or central or South America, and most studies in adult populations focus on breast cancer, likely due to the high incidence and relatively favourable prognosis. However, emerging evidence suggests that, for example, patients with haematological malignancies face a substantially higher cardiovascular risk, particularly heart failure, compared with many other types of cancer, in both the short and the long term.^48^ ^49^ ^50^ Historically, the poor survival in these patients may have diverted attention from cardiovascular outcomes, but recent improvements in outcomes underscore the need to include these patient populations in CTRCD risk prediction research.^51^ ^52^ In total we identified nine models that were developed and/or validated in haematological malignancies, of which five have been externally validated showing moderate performance (see supplementary table 13). Patients who have undergone a haematopoietic stem cell transplantation have been most studied, with all three available models externally validated.^53^ The CARE-BMT (Cardiovascular Registry in Bone Marrow Transplantation) showed the best discrimination (C statistic 0.77, 95% CI 0.72 to 0.83), but no information was provided on calibration. Further independent validations are necessary before this model can be considered the reference tool in this setting.

Among children, adolescents, and young adults, 16 out of 17 (94%) CTRCD prediction models were developed specifically for cancer survivors, of which four underwent external validation (see supplementary table 13). The models by Chow et al, based on easily available predictors (sex, age, and cancer treatment), showed variable performance across validation cohorts (C statistics 0.63 to 0.82; supplementary figure 1).^34^ In contrast, the tool developed by Leerink et al, externally validated once, showed good discrimination (C statistic 0.86, 95% CI 0.83 to 0.89) and calibration.^54^ Despite this model’s simplicity—comprising just three predictors—it includes baseline left ventricular ejection fraction, which may limit its applicability in some settings. Without further evidence, it is not possible to determine which of these tools performs best, as they were evaluated in studies adopting different outcome definitions and prediction horizons. This may explain why the updated recommendations for cardiomyopathy surveillance in children, adolescents, and young adult cancer survivors from the International Late Effects of Childhood Cancer Guideline Harmonization Group do not include any existing models.^55^ Instead, they proposed a risk stratification tool based on cumulative anthracycline and chest radiation doses, which, to the best of our knowledge, lacks formal methodological development and external validation.

Overall, only 12 (24%) of the 51 identified models developed to predict CTRCD have undergone external validation. Of these, seven were validated only once (see supplementary table 13), often within the original study or by the same research group. This raises major concerns about the generalisability of these models to different settings and populations, as well as the potential presence of publication bias and selective reporting.^56^ ^57^ Among the models validated more than once, unclear prediction horizons, heterogeneity in outcome definitions, and performance metric reporting further hindered robust evaluation of their accuracy.

The HFA-ICOS risk assessment tool, endorsed by the ESC,^7^ is currently the most extensively externally validated tool for risk stratification of cardiovascular toxicity in adults with cancer. Despite its prominence, our systematic review indicates that evidence supporting the clinical use of HFA-ICOS is insufficient. The tool is proposed to estimate the risk of cardiovascular toxicity for six distinct classes of cancer drugs, and as such it comprises six different models. Of the 11 external validation studies identified, most focused on the HFA-ICOS score for HER2 targeted therapies (n=5), followed by anthracyclines (n=3). Notably, no external validation studies were found for multiple myeloma therapies, and validation for patients treated with BCR-ABL or VEGF inhibitors was each limited to a single study (table 3). Additionally, while one study evaluated the tool in patients with melanoma treated with BRAF and MEK inhibitors, we excluded it from this review owing to small sample size (n=63).^58^

Across all external validation studies of the HFA-ICOS risk assessment tool (ie, regardless of class of antineoplastic drug), we identified an important mismatch between observed outcomes and expected risks. The tool consistently underestimated the risk of CTRCD and other cardiovascular events, with observed risks exceeding predictions across all risk strata, except the very high risk category (fig 5). This underestimation was most pronounced in studies where mild CTRCD comprised most events (see supplementary figure 2A), suggesting that the HFA-ICOS tool may not be suitable to predict milder forms of CTRCD, unless calibrated for that purpose. Furthermore, it is important to consider that the clinical significance of mild CTRCD has been questioned in recent studies conducted in patients with breast cancer treated with anthracyclines^59^ and trastuzumab,^60^ showing a low progression rate from mild to more severe forms of CTRCD during follow-up. Considering these findings, prospective cohort studies with sufficient follow-up are crucially needed to clarify the prognostic importance of mild CTRCD.

In studies focusing on moderate to severe or symptomatic CTRCD, the HFA-ICOS tool appears to perform better (see supplementary figure 2B). The largest validation to date included more than a thousand patients (mainly with breast cancer) undergoing treatment with anthracyclines, and reported a C statistic of 0.78 (95% CI 0.71 to 0.84) with good calibration to predict moderate or severe CTRCD.^41^ Interestingly, although mild CTRCD was not included in the outcome, 61% of patients fulfilled the definition of this complication, with high incidences across all risk strata (62% in low risk patients, 63% in moderate, 52% in high, and 30% in very high), again illustrating the tool’s poor calibration for such events. No other external validation studies have reported performance measures for the HFA-ICOS tool in anthracycline treated populations, and only a few have assessed its ability to predict more severe outcomes (see supplementary table 8).

Besides the questionable prognostic accuracy of the HFA-ICOS tool, its complexity raises concerns about practicality and cost effectiveness. Depending on the type of cancer systemic treatment, the tool incorporates 16 to 22 predictors—including demographic variables, cardiovascular risk factors, comorbidities, electrocardiographic (ECG) and cardiac imaging variables, serum biomarkers, and use of cancer treatments—some of which may be difficult to obtain, especially in resource limited settings. While the tool may be suitable for use by specialised cardio-oncology clinics, the comprehensive cardiovascular evaluation it requires limits its feasibility in routine oncology practice. Although the ESC guidelines propose a stepwise approach, starting cardiovascular toxicity risk assessment with easily available predictors (such as age, cardiovascular risk factors, and cancer treatment), followed by selective testing of cardiac biomarkers and echocardiography in patients with pre-existing cardiovascular disease or patients deemed at risk of CTRCD, this approach is not further substantiated.^7^

Simpler models may provide a practical alternative. For example, one study used an ECG model based on four variables, originally developed to predict sudden death in patients with coronary artery disease to predict CTRCD.^61^ The model showed good discrimination (C statistic 0.84, 95% CI 0.77 to 0.92), performing better than many more complex models included in this review (see supplementary figure 1). Similarly, the AI-ECG model, a deep learning model originally trained to detect left ventricular systolic dysfunction from ECG images,^62^ showed promising results in predicting left ventricular ejection fraction <40% in a cohort of patients with breast cancer or non-Hodgkin lymphoma (C statistic 0.82, 95% CI 0.74 to 0.89).^63^ These studies demonstrate the potential of simple and widely available tools, such as ECGs, to offer practical alternatives to more complex and resource demanding models, especially before referral of patients to specialised cardio-oncology care. Nonetheless, without head-to-head comparisons using the same population and outcomes, it remains unclear which models offer the optimal balance between feasibility and predictive performance.

### Strengths and limitations of this review

This study comprehensively summarised existing CTRCD risk prediction models for patients with cancer and cancer survivors undergoing systemic antineoplastic treatment. A previous systematic review focused on cardiotoxicity prediction models in patients with breast cancer^64^ identified comparable methodological limitations, including studies with a high risk of bias, and highlighted a widespread lack of external validation. Our review provides a broader and more rigorous synthesis of models developed and validated across diverse cancer populations and systemic treatment regimens. Additionally, our study offers a detailed assessment of the predictive performance of the HFA-ICOS risk assessment tool, including a meta-analysis of its performance in patients with breast cancer receiving HER2 targeted therapies, further advancing the understanding of its prognostic value.

This systematic review also has some limitations. Heterogeneity was present among the studies included in the meta-analyses, particularly in the definition of CTRCD, the use of composite outcomes incorporating other cardiovascular events, and varying follow-up durations. This variability likely reflects the lack of guidance on the intended use of the HFA-ICOS tool, including the outcomes targeted by the models and the intended prediction horizon, which may influence the pooled estimates.^7^ ^9^ Furthermore, by focusing exclusively on systemic cancer therapies, we excluded studies on radiation induced cardiotoxicity, which may have reduced the representation of certain malignancies such as lung cancer. Likewise, malignancies treated with hormonal therapy, such as prostate cancer, were excluded, limiting the applicability of our findings to these populations.

### Implications and recommendations for future research

Accurate prediction of CTRCD risk carries important clinical implications. Early identification of high risk individuals before the initiation of cancer treatment creates a window of opportunity for the initiation of cardioprotective strategies, can aid in the selection or modification of cancer therapies to regimens associated with less cardiotoxicity,^65^ and facilitate closer cardiac monitoring during and after treatment.^66^ Evidence suggests that such proactive approaches can reduce the incidence and severity of cardiotoxicity, potentially improving long term cardiovascular outcomes.^67^ ^68^ ^69^ ^70^ ^71^ ^72^ Importantly, since referral of all patients to specialised cardio-oncology clinics is not feasible, oncologists have a central role in recognising and assessing cardiovascular risk, ensuring referral to specialised care, when appropriate. Primary care doctors, as key members of long term patient care, play a complementary role and should be alert to the heightened cardiovascular risk in patients with cancer and cancer survivors, in both the short and the long term, to enable timely screening and early diagnosis of cardiovascular disease in this population. This underscores the need for simple, accessible risk stratification tools.

The findings from our study offer important insights for clinical practice and future research. While the HFA-ICOS risk assessment tool is currently recommended for clinical use by the ESC, its limited discriminative ability and poor calibration present challenges for effective decision making. Until further evidence substantiates its performance, caution is warranted when applying this model in routine practice. High quality prospective studies with adequate sample sizes are needed to validate and recalibrate the models included in the HFA-ICOS tool. These studies should ideally use standardised outcome definitions, define prediction horizons tailored to specific malignancies and cancer treatments, and appropriately weight predictors based on their relative contribution to CTRCD risk. Moreover, after these improvements, implementation studies are crucial to evaluate whether the use of this tool improves clinical outcomes, decision making, and cost effectiveness in real world settings.

Certain subgroups of patients with cancer remain substantially underrepresented in the development and validation of CTRCD prediction models, including children, adolescents, and young adults with active cancer, adults with malignancies other than breast cancer, and populations from non-Western regions. While conducting external validation studies across all cancers and cardiotoxic treatments may be infeasible, leveraging large, linked, or pooled datasets from different sources can enable direct comparisons of multiple models simultaneously,^73^ ^74^ and assessment of model transportability and generalisability across different populations and clinical settings. This approach can ultimately optimise research efforts and provide a solid foundation for model updating or recalibration tailored to specific patient groups. Moreover, individual participant data meta-analyses with internal-external cross validation are emerging as robust strategies for both developing and validating prediction models while simultaneously assessing their generalisability.^73^ ^75^ The growing availability of cardio-oncology registries makes such approaches increasingly feasible.^76^ ^77^ ^78^ However, it is of importance that a diverse case mix is included in such registries, encompassing patients of all risk levels rather than focusing solely on groups of patients who are perceived as high risk based on current clinical knowledge.

Lastly, the integration of machine learning approaches offers a promising avenue to enhance CTRCD risk prediction, as these methods have the potential to capture complex relations among many clinical, biochemical, and imaging variables, particularly relevant in oncology patients receiving multiple treatment regimens. However, despite their potential, machine learning models involve important challenges. Their complexity and occasional lack of interpretability can hinder both external validation efforts and clinical uptake. Ensuring transparency, prospective validation, and alignment with clinical workflows will be key steps toward their successful implementation.^79^ Additionally, while these models are often developed by engineers and data scientists, their utility in healthcare will be enhanced when developed in close collaboration with clinicians who can help define outcomes rigorously, understand the underlying biases of the data used, and ensure that clinical reasoning remains central to the decision making process.

### Conclusion

Over the past decades, the prognosis of many malignancies has steadily improved, and cancer is increasingly regarded as a chronic disease. However, the cardiovascular toxicity associated with many antineoplastic treatments is a growing concern. This systematic review provides a comprehensive overview of available CTRCD risk prediction models. Despite recent efforts to improve CTRCD risk stratification in patients with cancer and cancer survivors, no prediction model has consistently shown reliable performance. The HFA-ICOS risk assessment tool currently proposed by the ESC guidelines,^7^ ^9^ is the most extensively validated tool, but we conclude that its prognostic accuracy is suboptimal. To advance the discipline, using standardised outcome definitions and prediction horizons is essential to ensure consistency in external validation studies. Additionally, more rigorous research is needed to validate and refine existing models across various cancer types and to evaluate their impact when implemented in routine clinical practice.

**What is already known on this topic**

Cancer therapy related cardiac dysfunction (CTRCD) is a major complication of cancer treatment, driven by both patient specific and treatment related factors
Given the high global burden of cancer and the widespread use of antineoplastic agents associated with CTRCD, effective risk stratification models are urgently needed
It remains uncertain which CTRCD risk prediction models show the strongest predictive performance and are best suited for clinical application

**What this study adds**

Almost all existing CTRCD risk prediction models show a high risk of bias, raising concerns about the reliability of their predictions when applied in routine clinical practice
This study identified underrepresentation of data from patients with malignancies other than breast cancer and a scarcity of externally validated models
The findings of this review highlight the need for greater methodological rigour, standardised outcome definitions and prediction horizons, and external validation of models across diverse cancer types

## Data Availability

Additional supporting data are available in the supplementary file.

## Web extra

Extra material supplied by authors

Supplementary information: Tables 1-15 and figures 1-5

Supplementary information: Additional data to share

## Notes

Contributors: CG and ML conceived the study and developed the protocol, and together with AM, they defined the query for the electronic database search. CG and ML screened studies for eligibility, consulting TPAD in cases of disagreement. CG and JG extracted the data. CG analysed the data. CG and ML wrote the first draft of the manuscript, which was thereafter critically revised by all authors. All authors approved the final version of the submitted manuscript. CG and ML are the guarantors, had full access to the study data, and are responsible for its integrity and the accuracy of the analysis. The corresponding author attests that all listed authors meet authorship criteria and that no others meeting the criteria have been omitted.

Transparency: The lead author affirms that the manuscript is an honest, accurate, and transparent account of the study being reported; that no important aspects of the study have been omitted; and that any discrepancies from the study as planned have been explained.

Dissemination to participants and related patient and public communities: The results of this study will be disseminated through presentations at national and international conferences. Results will also be shared through social media channels to reach a broader audience.

Provenance and peer review: Not commissioned; externally peer reviewed.
