# Supplementary material for: Risk prediction models for cancer therapy related cardiac dysfunction in patients with cancer and cancer survivors: systematic review and meta-analysis
Source: BMJ. 2025 Sep 23;390:e084062. doi: 10.1136/bmj-2025-084062 (PMC12492976; doi:10.1136/bmj-2025-084062)
Supplement: Supplementary file 1 — Supplementary information: Tables 1-15 and figures 1-5 [file gomc084062.ww1.pdf]

SUPPLEMENTAL APPENDIX

TABLE OF CONTENTS

Supplemental Table 1. TRIPOD-SRMA checklist...2

Supplemental Table 2. PRISMA 2020 checklist. 4

Supplemental Table 3. PRISMA 2020 for Abstracts Checklist. 7

Supplemental Table 4. PRISMA-S checklist for Reporting Literature Searches. 8

Supplemental Table 5. Search strategies used for MEDLINE, EMBASE, and the Cochrane Central Register of Controlled Trials 10

Supplemental Table 6. Categorization of predictors..13

Supplemental Table 7. Outcome definitions across studies and their categorization 13

Supplemental Table 8. Observed outcomes across HFA-ICOS external validation studies, categorized by clinical relevance. 17

Supplemental Table 9. List of articles that were excluded after full-text assessment despite presenting a prediction model or performing multivariable modeling 19

Supplemental Table 10. Detailed characteristics of included development and external validation studies.21

Supplemental Table 11. Models that were identified from external validation studies, but of which the original development study did not fulfill the inclusion criteria of this systematic review 29

Supplemental Table 12. Overview of predictor groups included in each model...30

Supplemental Table 13. Performance and characteristics of included models in development and external validation settings. 33

Supplemental Table 14. Risk of bias and applicability assessment using the PROBAST tool for each model development and external validation...40

Supplemental Table 15. PROBAST signaling question scores per study/model 45

Supplemental Figure 1. Distribution of C-statistics in the development (with or without internal validation) and the external validation samples. 51

Supplemental Figure 2. Calibration of the HFA-ICOS risk assessment tool across external validation studies targeting CTRCD. 52

Supplemental Figure 3. Forest plots of the meta-analyses illustrating observed risks across all external validation studies in patients treated with Her2-targeted therapies per HFA-ICOS risk strata. 53

Supplemental Figure 4. Forest plots of the meta-analyses of observed risks across HFA-ICOS risk strata, after excluding studies with high applicability concerns..54

Supplemental Figure 5. Forest plot of the meta-analysis of C-statistic measures across external validation studies of the HFA-ICOS risk assessment tool in patients receiving Her2-targeted therapies, after excluding studies with high applicability concerns. 55

Supplemental Table 1. TRIPOD-SRMA checklist

Section/topic	No.	Checklist item	Location where item is reported
**Title**			
Title	1	Identify the report as a systematic review or meta-analysis (or both) of diagnostic or prognostic model studies. Specify the target population and outcome(s) predicted as relevant to the review question.	Page 1
**Abstract**			
Abstract	2	See the TRIPOD-SRMA Checklist for Abstracts	Page 1
**Introduction**			
Rationale	3	Describe the rationale for the review in the context of existing knowledge.	Page 2
Objectives	4	Provide an explicit statement of the objective(s) being addressed with reference to: target population, index and comparator models (as relevant), outcome(s), time (prediction horizon and intended moment of using the model), and setting.	Page 2
**Methods**			
Study eligibility criteria	5	Specify study characteristics used as eligibility criteria, including any prediction models of specific interest, and whether development or validation studies (or both) were eligible.	Page 2
Information sources	6	Specify all databases, registers, websites, organizations, reference lists and other sources searched or consulted to identify studies. Specify the date when each source was last searched or consulted.	Page 2
Search strategy	7	Present the full search strategies for all databases, registers and websites, including any filters and limits used.	Table S5
Study selection process	8	Specify the methods used to decide whether a study met the inclusion criteria of the review, including how many reviewers screened each record and each report retrieved, whether they worked independently, and if applicable, details of automation tools used in the process.	Page 2
Data collection process	9	Specify the methods used to collect data from study reports, including how many reviewers collected data from each report, whether they worked independently, any processes for obtaining or confirming data from study investigators, and if applicable, details of automation tools used in the process.	Pages 2-3
Data items	10a	List and define all items for which data were sought from each study.	Page 2
	10b	State the model performance measures that were sought (e.g., measures of calibration, discrimination, overall model fit, clinical utility).	Page 2
	10c	Describe how any desired but unreported data items (items 10a, 10b) were handled (e.g., contacted authors, calculated from other reported information).	Page 3

Risk of bias and applicability assessment	11	Specify the methods used to assess risk of bias in the included studies and their applicability to the review question. This should be done separately for each model development and validation. Include details of any tool(s) used, how many reviewers assessed each study and whether they worked independently.	Page 3
Synthesis methods	12a	Describe any methods for synthesizing estimates of performance measures for each model. If meta-analysis was carried out, describe the methods used, including any transformations of data prior to pooling, how any heterogeneity in model performance was quantified and handled, and software package(s) used.	Page 3
	12b	Describe any methods used to explore possible causes of heterogeneity in model performance (e.g., subgroup analysis, meta-regression), including whether or not they were planned.	Page 3
	12c	Describe any sensitivity analyses conducted to assess robustness of the synthesized results.	Page 3
Certainty assessment	13	Describe any methods used to assess certainty (or confidence) in the body of evidence for a prediction model.	Not performed
**Results**			
Study selection	14	Describe the results of the search and selection process, from the number of records identified in the search to the number of studies and models included in the review, ideally using a flow diagram.	Page 3, Figure 1
Study and model characteristics	15	Present study characteristics and model details extracted (as per Item 10a), and cite the study reports.	Table S10
Risk of bias and applicability	16	Present results of risk of bias and applicability assessment. This should be done separately for each model development and validation in each included study.	Tables S14 and S15
Results of model performance in individual studies	17	Present performance estimates and confidence intervals for each model and all evaluations, including whether they relate to the internal or external validation performance. If internal, give details of the method.	Table S13, Figure S1
Results of syntheses	18a	Present the results of any synthesis of model performance, together with details of which study estimates contributed. If meta-analysis was carried out, then for each model and performance measure, present summary results, confidence/credible intervals and measures of heterogeneity. Forest plots may be useful.	Page 12, Figures 6 and S3
	18b	For each model, present results of all investigations of possible causes of heterogeneity in model performance.	Page 11-12, Figure S2
	18c	Present results of all sensitivity analyses conducted to assess the robustness of the synthesized results.	Figures S4 and S5
Certainty of evidence	19	Present any assessments of certainty (or confidence) in the body of evidence for each prediction model of interest.	Not performed
**Discussion**			
Summary of evidence	20	Summarize the main findings including the strengths and limitations of the evidence.	Page 13
Limitations	21	Discuss the strengths and limitations of the review process.	Page 15
Implications	22	Discuss implications of the results in the context of other evidence and for practice, policy, and future research.	Page 15

Other information			
Registration and protocol	23a	Provide registration information for the review, including register name and registration number, or state that the review was not registered.	Page 1
	23b	Indicate where the review protocol can be accessed, or state that a protocol was not prepared.	Page 2
	23c	Describe and explain any amendments to information provided at registration or in the protocol.	Page 2
Support	24	Describe sources of financial or non-financial support for the review, and the role of the funders or sponsors in the review.	Page 16
Competing interests	25	Declare any competing interests of review authors.	Page 16
Availability of data, code, and other materials	26	Report which of the following are publicly available and where they can be found: template data collection forms; data extracted from included studies; data used for all analyses; analytic code; any other materials used in the review.	Page 16

This checklist appears in appendix 2 of Snell KIE, Levis B, Damen JAA, et al. Transparent reporting of multivariable prediction models for individual prognosis or diagnosis: checklist for systematic reviews and meta-analyses (TRIPOD-SRMA). *BMJ* 2023;381:e073538. doi:10.1136/bmj-2022-073538.

S, supplemental materials.

Supplemental Table 2. PRISMA 2020 checklist.

Section and Topic	Item #	Checklist item	Location where item is reported
**TITLE**			
Title	1	Identify the report as a systematic review.	Page 1
**ABSTRACT**			
Abstract	2	See the PRISMA 2020 for Abstracts checklist.	Page 1
**INTRODUCTION**			
Rationale	3	Describe the rationale for the review in the context of existing knowledge.	Pages 1-2
Objectives	4	Provide an explicit statement of the objective(s) or question(s) the review addresses.	Page 2
**METHODS**			
Eligibility criteria	5	Specify the inclusion and exclusion criteria for the review and how studies were grouped for the syntheses.	Page 2
Information sources	6	Specify all databases, registers, websites, organisations, reference lists and other sources searched or consulted to identify studies. Specify the date when each source was last searched or consulted.	Page 2
Search strategy	7	Present the full search strategies for all databases, registers and websites, including any filters and limits used.	Table S5
Selection process	8	Specify the methods used to decide whether a study met the inclusion criteria of the review, including how many reviewers screened each record and each report retrieved, whether they worked independently, and if applicable, details of automation tools used in the process.	Page 2

Section and Topic	Item #	Checklist item	Location where item is reported
Data collection process	9	Specify the methods used to collect data from reports, including how many reviewers collected data from each report, whether they worked independently, any processes for obtaining or confirming data from study investigators, and if applicable, details of automation tools used in the process.	Pages 2-3
Data items	10a	List and define all outcomes for which data were sought. Specify whether all results that were compatible with each outcome domain in each study were sought (e.g. for all measures, time points, analyses), and if not, the methods used to decide which results to collect.	Pages 2-3
	10b	List and define all other variables for which data were sought (e.g. participant and intervention characteristics, funding sources). Describe any assumptions made about any missing or unclear information.	Page 2
Study risk of bias assessment	11	Specify the methods used to assess risk of bias in the included studies, including details of the tool(s) used, how many reviewers assessed each study and whether they worked independently, and if applicable, details of automation tools used in the process.	Page 3
Effect measures	12	Specify for each outcome the effect measure(s) (e.g. risk ratio, mean difference) used in the synthesis or presentation of results.	Page 3
Synthesis methods	13a	Describe the processes used to decide which studies were eligible for each synthesis (e.g. tabulating the study intervention characteristics and comparing against the planned groups for each synthesis (item #5)).	Pages 3
	13b	Describe any methods required to prepare the data for presentation or synthesis, such as handling of missing summary statistics, or data conversions.	Pages 3
	13c	Describe any methods used to tabulate or visually display results of individual studies and syntheses.	Page 3
	13d	Describe any methods used to synthesize results and provide a rationale for the choice(s). If meta-analysis was performed, describe the model(s), method(s) to identify the presence and extent of statistical heterogeneity, and software package(s) used.	Page 3
	13e	Describe any methods used to explore possible causes of heterogeneity among study results (e.g. subgroup analysis, meta-regression).	Page 3
	13f	Describe any sensitivity analyses conducted to assess robustness of the synthesized results.	Page 3
Reporting bias assessment	14	Describe any methods used to assess risk of bias due to missing results in a synthesis (arising from reporting biases).	Not performed
Certainty assessment	15	Describe any methods used to assess certainty (or confidence) in the body of evidence for an outcome.	Not performed
**RESULTS**			
Study selection	16a	Describe the results of the search and selection process, from the number of records identified in the search to the number of studies included in the review, ideally using a flow diagram.	Page 3, Figure 1
	16b	Cite studies that might appear to meet the inclusion criteria, but which were excluded, and explain why they were excluded.	Table S9, Page 3
Study characteristics	17	Cite each included study and present its characteristics.	Table S10

Section and Topic	Item #	Checklist item	Location where item is reported
Risk of bias in studies	18	Present assessments of risk of bias for each included study.	Tables S14, S15
Results of individual studies	19	For all outcomes, present, for each study: (a) summary statistics for each group (where appropriate) and (b) an effect estimate and its precision (e.g. confidence/credible interval), ideally using structured tables or plots.	Figure S1, Table S13
Results of syntheses	20a	For each synthesis, briefly summarise the characteristics and risk of bias among contributing studies.	Table 3
	20b	Present results of all statistical syntheses conducted. If meta-analysis was done, present for each the summary estimate and its precision (e.g. confidence/credible interval) and measures of statistical heterogeneity. If comparing groups, describe the direction of the effect.	Page 12, Figures 6, S3, S4, S5
	20c	Present results of all investigations of possible causes of heterogeneity among study results.	Figure S2, Page 11
	20d	Present results of all sensitivity analyses conducted to assess the robustness of the synthesized results.	Page 12, Figures S4, S5
Reporting biases	21	Present assessments of risk of bias due to missing results (arising from reporting biases) for each synthesis assessed.	Not performed
Certainty of evidence	22	Present assessments of certainty (or confidence) in the body of evidence for each outcome assessed.	Not performed
**DISCUSSION**			
Discussion	23a	Provide a general interpretation of the results in the context of other evidence.	Pages 13-15
	23b	Discuss any limitations of the evidence included in the review.	Pages 13-15
	23c	Discuss any limitations of the review processes used.	Page 15
	23d	Discuss implications of the results for practice, policy, and future research.	Page 15
**OTHER INFORMATION**			
Registration and protocol	24a	Provide registration information for the review, including register name and registration number, or state that the review was not registered.	Page 1
	24b	Indicate where the review protocol can be accessed, or state that a protocol was not prepared.	Page 2
	24c	Describe and explain any amendments to information provided at registration or in the protocol.	Page 2
Support	25	Describe sources of financial or non-financial support for the review, and the role of the funders or sponsors in the review.	Page 16
Competing interests	26	Declare any competing interests of review authors.	Page 16

Section and Topic	Item #	Checklist item	Location where item is reported
Availability of data, code and other materials	27	Report which of the following are publicly available and where they can be found: template data collection forms; data extracted from included studies; data used for all analyses; analytic code; any other materials used in the review.	Page 16

From: Page MJ, McKenzie JE, Bossuyt PM, Boutron I, Hoffmann TC, Mulrow CD, et al. The PRISMA 2020 statement: an updated guideline for reporting systematic reviews. BMJ 2021;372:n71. doi: 10.1136/bmj.n71.
This work is licensed under CC BY 4.0. To view a copy of this license, visit https://creativecommons.org/licenses/by/4.0/

Supplemental Table 3. PRISMA 2020 for Abstracts Checklist.

Section and Topic	Item #	Checklist item	Location where item is reported
**TITLE**			
Title	1	Identify the report as a systematic review.	Page 1
**BACKGROUND**			
Objectives	2	Provide an explicit statement of the main objective(s) or question(s) the review addresses.	Page 1
**METHODS**			
Eligibility criteria	3	Specify the inclusion and exclusion criteria for the review.	Page 1
Information sources	4	Specify the information sources (e.g. databases, registers) used to identify studies and the date when each was last searched.	Page 1
Risk of bias	5	Specify the methods used to assess risk of bias in the included studies.	Page 1
Synthesis of results	6	Specify the methods used to present and synthesise results.	Page 1
**RESULTS**			
Included studies	7	Give the total number of included studies and participants and summarise relevant characteristics of studies.	Page 1
Synthesis of results	8	Present results for main outcomes, preferably indicating the number of included studies and participants for each. If meta-analysis was done, report the summary estimate and confidence/credible interval. If comparing groups, indicate the direction of the effect (i.e. which group is favoured).	Page 1
**DISCUSSION**			
Limitations of evidence	9	Provide a brief summary of the limitations of the evidence included in the review (e.g. study risk of bias, inconsistency and imprecision).	Page 1
Interpretation	10	Provide a general interpretation of the results and important implications.	Page 1

OTHER			
Funding	11	Specify the primary source of funding for the review.	Page 16
Registration	12	Provide the register name and registration number.	Page 1

From: Page MJ, McKenzie JE, Bossuyt PM, Boutron I, Hoffmann TC, Mulrow CD, et al. The PRISMA 2020 statement: an updated guideline for reporting systematic reviews. BMJ 2021;372:n71. doi: 10.1136/bmj.n71
S, supplemental materials.

Supplemental Table 4. PRISMA-S checklist for Reporting Literature Searches.

Section/topic	#	Checklist item	Location where item is reported
**INFORMATION SOURCES AND METHODS**			
Database name	1	Name each individual database searched, stating the platform for each.	Page 2
Multi-database searching	2	If databases were searched simultaneously on a single platform, state the name of the platform, listing all of the databases searched.	Not performed
Study registries	3	List any study registries searched.	Not performed
Online resources and browsing	4	Describe any online or print source purposefully searched or browsed (e.g., tables of contents, print conference proceedings, web sites), and how this was done.	Not performed
Citation searching	5	Indicate whether cited references or citing references were examined, and describe any methods used for locating cited/citing references (e.g., browsing reference lists, using a citation index, setting up email alerts for references citing included studies).	Page 2
Contacts	6	Indicate whether additional studies or data were sought by contacting authors, experts, manufacturers, or others.	Not performed
Other methods	7	Describe any additional information sources or search methods used.	Not performed
**SEARCH STRATEGIES**			
Full search strategies	8	Include the search strategies for each database and information source, copied and pasted exactly as run.	Table S5
Limits and restrictions	9	Specify that no limits were used, or describe any limits or restrictions applied to a search (e.g., date or time period, language, study design) and provide justification for their use.	Page 2

Search filters	10	Indicate whether published search filters were used (as originally designed or modified), and if so, cite the filter(s) used.	Page 2
Prior work	11	Indicate when search strategies from other literature reviews were adapted or reused for a substantive part or all of the search, citing the previous review(s).	Not performed
Updates	12	Report the methods used to update the search(es) (e.g., rerunning searches, email alerts).	Page 2
Dates of searches	13	For each search strategy, provide the date when the last search occurred.	Page 2
**PEER REVIEW**			
Peer review	14	Describe any search peer review process.	Not performed
**MANAGING RECORDS**			
Total Records	15	Document the total number of records identified from each database and other information sources.	Table S5
Deduplication	16	Describe the processes and any software used to deduplicate records from multiple database searches and other information sources.	Page 2

PRISMA-S: An Extension to the PRISMA Statement for Reporting Literature Searches in Systematic Reviews

Rethlefsen ML, Kirtley S, Waffenschmidt S, Ayala AP, Moher D, Page MJ, Koffel JB, PRISMA-S Group.

Last updated February 27,
2020.
S, supplemental materials.

Supplemental Table 5. Search strategies used for MEDLINE, EMBASE, and the Cochrane Central Register of Controlled Trials

#	Searches
**MEDLINE (Ovid)**	
**1**	*Medical Oncology/ or *Neoplasms/
**2**	(tumo?r* or neoplas* or cancer* or malignanc* or metasta* or oncolog*).ti,ab,kf.
**3**	(carcinoma* or lymphoma* or macroglobulinemia or leukemia* or sarcoma* or Hodgkin or glioma* or melanoma* or myeloma* or blastoma*).ti,ab,kf.
**4**	(h?ematopoietic adj3 "cell transplant*").ti,ab,kf.
**5**	or/1-4
**6**	exp ROC Curve/ or exp "Predictive Value of Tests"/ or exp Area Under Curve/ or exp Risk Assessment/ or exp "Sensitivity and Specificity"/ or exp Logistic Models/
**7**	(risk adj3 (assess* or factor* or stratif* or evaluat* or analys* or model* or value* or score* or indices or index* or equation* or function* or chart* or tool* or calculat*)).ti,ab,kf.
**8**	(predict* or sensitivity or specificity or calibrat* or discriminat*).ti,ab,kf.
**9**	(AUC or ("area under" adj3 curve)).ti,ab,kf.
**10**	(("receiver operating" or ROC) adj3 (characteristic* or curve* or analys*)).ti,ab,kf.
**11**	(c adj2 statistic*).ti,ab,kf.
**12**	(logistic adj3 (regression or analysis or model)).ti,ab,kf.
**13**	(regression adj3 (multivaria* or multiple)).ti,ab,kf.
**14**	(prognostic adj3 (history or variable* or criteria or scor* or characteristic* or finding* or factor* or model*)).ti,ab,kf.
**15**	(decision* adj5 (model* or clinical* or history or variable* or criteria or scor* or characteristic* or finding* or factor*)).ti,ab,kf.
**16**	or/6-15
**17**	*Cardiotoxicity/ or *Heart Failure/ or *Cardiomyopathies/
**18**	((heart or cardiac or myocardial) adj3 (fail* or decompensation or dysfunction*)).ti,ab,kf.
**19**	(cardiotoxic* or cardiomyopath* or ((heart or cardi* or myocardial) adj3 toxic*)).ti,ab,kf.
**20**	(fraction* adj3 (shortening or ejection)).ti,ab,kf.
**21**	(GLS or (global adj3 "longitudinal strain")).ti,ab,kf.
**22**	(systolic adj3 (dysfunction or fail*)).ti,ab,kf.
**23**	or/17-22
**24**	exp Controlled Clinical Trial/ or exp Intention to Treat Analysis/ or exp cross-over studies/ or exp double-blind method/ or exp random allocation/ or exp single-blind method/ or exp Equivalence Trial/ or exp Clinical Trial/ or exp Cohort Studies/ or exp follow-up studies/ or exp longitudinal studies/ or exp prospective studies/ or exp retrospective studies/ or *clinical study/ or exp Registries/
**25**	((cohort* or longitudinal* or follow up or followup or prospective or retrospective) adj2 (stud* or design* or analy* or survey* or trial*)).ti,ab,kf.
**26**	(randomized or randomly or placebo* or allocat* or retrospective* or prospective*).ti,ab,kf.
**27**	((control* or multicentre* or multicentr* or crossover or "cross over" or clinical* or "intention to treat" or equivalence) adj2 (study or studies or trial* or group* or cohort*)).ti,ab,kf.
**28**	((singl* or doubl* or tripl*) adj2 (blind* or dumm* or mask*)).ti,ab,kf.
**29**	((clinical or pragmatic) adj2 (trial* or study)).ti,ab,kf.
**30**	(patient* adj2 Registr*).ti,ab,kf.
**31**	or/24-30
**32**	((exp animals/ or exp veterinary medicine/ or animal*.jw.) not exp humans/) or (experiment* model* or in vitro or animal* or monkey* or sheep or ?ovine or lamb* or goat* or pig* or swine or porcine or pup* or dog* or canine or bitch* or beagle* or feline or rodent* or rabbit* or rat or rats or mouse or murine or mice).ti,kf.
**33**	exp Case Reports/ or exp "Review"/ or exp Editorial/ or exp Letter/
**34**	or/32-33
**35**	5 and 16 and 23 and 31
**36**	35 not 34
**37**	(International adj2 (Cardio adj1 Oncology) adj2 Society).ti,ab,kf.
**38**	("HFA-ICOS" or HFAICOS or "HFA ICOS").ti,ab,kf.

**39**	or/37-38
**40**	36 or 39

**EMBASE (Ovid)**	
**1**	*oncology/ or *malignant neoplasm/
**2**	(tumo?r* or neoplas* or cancer* or malignanc* or metasta* or oncolog*).ti,ab,kf.
**3**	(carcinoma* or lymphoma* or macroglobulinemia or leukemia* or sarcoma* or Hodgkin or glioma* or melanoma* or myeloma* or blastoma*).ti,ab,kf.
**4**	(h*ematopoietic adj3 "cell transplant*").ti,ab,kf.
**5**	or/1-4
**6**	exp prediction/ or exp receiver operating characteristic/ or exp area under the curve/ or exp risk assessment/ or exp "sensitivity and specificity"/ or exp logistic regression analysis/ or multiple linear regression analysis/ or exp multivariate logistic regression analysis/
**7**	(risk adj3 (assess* or factor* or stratif* or evaluat* or analys* or model* or value* or score* or indices or index* or equation* or function* or chart* or tool* or calculat*)).ti,ab,kf.
**8**	(predict* or sensitivity or specificity or calibrat* or discriminat*).ti,ab,kf.
**9**	(AUC or ("area under" adj3 curve)).ti,ab,kf.
**10**	(("receiver operating" or ROC) adj3 (characteristic* or curve* or analys*)).ti,ab,kf.
**11**	(c adj2 statistic*).ti,ab,kf.
**12**	(logistic adj3 (regression or analysis or model)).ti,ab,kf.
**13**	(regression adj3 (multivaria* or multiple)).ti,ab,kf.
**14**	(prognostic adj3 (history or variable* or criteria or scor* or characteristic* or finding* or factor* or model*)).ti,ab,kf.
**15**	(decision* adj5 (model* or clinical* or history or variable* or criteria or scor* or characteristic* or finding* or factor*)).ti,ab,kf.
**16**	or/6-15
**17**	*cardiotoxicity/ or *heart failure/ or *cardiomyopathy/
**18**	((heart or cardiac or myocardial) adj3 (fail* or incompetenc* or insufficien* or decompensation or dysfunction*)).ti,ab,kf.
**19**	(cardiotoxic* or cardiomyopath* or HFrEF or ((heart or cardi* or myocardial) adj3 toxic*)).ti,ab,kf.
**20**	(fraction* adj3 (shortening or ejection)).ti,ab,kf.
**21**	(GLS or (global adj3 "longitudinal strain")).ti,ab,kf.
**22**	((systolic or diastolic) adj3 (dysfunction or fail*)).ti,ab,kf.
**23**	or/17-22
**24**	exp controlled clinical trial/ or exp crossover procedure/ or exp double blind procedure/ or exp intention to treat analysis/ or exp single blind procedure/ or exp triple blind procedure/ or exp randomization/ or exp equivalence trial/ or exp clinical trial/ or exp "clinical trial (topic)"/ or exp cohort analysis/ or longitudinal study/ or prospective study/ or retrospective study/ or exp follow up/ or "major clinical study"/ or exp patient registry/
**25**	((cohort* or longitudinal* or follow up or followup or prospective or retrospective) adj2 (stud* or design* or analy* or survey* or trial*)).ti,ab,kf.
**26**	(randomized or randomly or sham or placebo* or allocat* or retrospective* or prospective*).ti,ab,kf.
**27**	((control* or multicentre* or multicentr* or crossover or "cross over" or clinical* or "intention to treat" or equivalence) adj2 (study or studies or trial* or group* or cohort*)).ti,ab,kf.
**28**	((singl* or doubl* or tripl*) adj2 (blind* or dumm* or mask*)).ti,ab,kf.
**29**	((clinical or pragmatic) adj2 (trial* or study)).ti,ab,kf.
**30**	(patient* adj2 Registr*).ti,ab,kf.
**31**	or/24-30
**32**	((exp animals/ or exp veterinary medicine/ or animal*.jw.) not exp humans/) or (experiment* model* or in vitro or animal* or monkey* or sheep or ?ovine or lamb* or goat* or pig* or swine or porcine or pup* or dog* or canine or bitch* or beagle* or feline or rodent* or rabbit* or rat or rats or mouse or murine or mice).ti,kf.
**33**	exp case report/ or exp "review"/ or exp conference abstract/ or exp editorial/ or exp letter/ or exp note/
**34**	or/32-33
**35**	(International adj2 (Cardio adj1 Oncology) adj2 Society).ti,ab,kf.
**36**	("HFA-ICOS" or HFAICOS or "HFA ICOS").ti,ab,kf.
**37**	or/35-36
**38**	5 and 16 and 23 and 31

**39**	38 not 34
**40**	37 or 39

**Cochrane Central Register of Controlled Trials (CENTRAL)**	
**1**	MeSH descriptor: [Medical Oncology] this term only
**2**	MeSH descriptor: [Neoplasms] this term only
**3**	(tumo?r* or neoplas* or cancer* or malignanc* or metasta* or oncolog*)
**4**	(carcinoma* or lymphoma* or macroglobulinemia or leukemia* or sarcoma* or Hodgkin or glioma* or melanoma* or myeloma or blastoma*)
**5**	(h?ematopoietic near/3 "cell transplant")
**6**	{or #1-#5}
**7**	MeSH descriptor: [ROC Curve] explode all trees
**8**	MeSH descriptor: [Predictive Value of Tests] explode all trees
**9**	MeSH descriptor: [Area Under Curve] explode all trees
**10**	MeSH descriptor: [Risk Assessment] explode all trees
**11**	MeSH descriptor: [Sensitivity and Specificity] explode all trees
**12**	MeSH descriptor: [Logistic Models] explode all trees
**13**	(risk near/3 (assess* or factor* or stratif* or evaluat* or analys* or model* or value* or score* or indices or index* or equation* or function* or chart* or tool* or calculat*))
**14**	(predict* or sensitivity or specificity or calibrat* or discriminat*)
**15**	(AUC or ("area under" near/3 curve))
**16**	(("receiver operating" or ROC) near/3 (characteristic* or curve* or analys*))
**17**	(c near/2 statistic*)
**18**	(logistic near/3 (regression or analysis or model))
**19**	(regression near/3 (multivaria* or multiple))
**20**	(prognostic near/3 (history or variable* or criteria or scor* or characteristic* or finding* or factor* or model*))
**21**	(decision* near/5 (model* or clinical* or history or variable* or criteria or scor* or characteristic* or finding* or factor*))
**22**	{or #7-#21}
**23**	MeSH descriptor: [Cardiotoxicity] this term only
**24**	MeSH descriptor: [Heart Failure] this term only
**25**	MeSH descriptor: [Cardiomyopathies] this term only
**26**	((heart or cardiac or myocardial) near/3 (fail* or decompensation or dysfunction*))
**27**	(cardiotoxic* or cardiomyopath* or ((heart or cardi* or myocardial) near/3 toxic*))
**28**	(fraction* near/3 (shortening or ejection))
**29**	(GLS or (global near/3 "longitudinal strain"))
**30**	((systolic) near/3 (dysfunction or fail*))
**31**	{or #23-#30}
**32**	#6 AND #22 AND #31
**33**	((experiment* model*) or "in vitro" or animal* or monkey* or sheep or ?ovine or lamb* or goat* or pig* or swine or porcine or pup* or dog* or canine or bitch* or beagle* or feline or rodent* or rabbit* or rat or rats or mouse or murine or mice)
**34**	#32 not #33

The search was conducted on September 21, 2023, and updated August 23, 2024. Number of records identified from each database: MEDLINE - 4,429 records; EMBASE - 8,840 records, Cochrane Central Register of Controlled Trials (CENTRAL) - 1,649 records.

Supplemental Table 6. Categorization of predictors

Predictors included across developed models	Categorized as
(1) Two principal components for ethnicity; (2) race.	Ethnicity
(1) Hypertension; (2) systolic BP; (3) diastolic BP; (4) BP ≥ 140/90 mmHg; (5) BP variability at ABPM; (6) pulse wave velocity at arterial stiffness estimation; (7) treatment with ACEi/ARB.	Hypertension
(1) Dyslipidemia; (2) hypercholesterolemia; (3) triglycerides ≥ 150 mg/dL.	Dyslipidemia
(1) Obesity; (2) weight ≥ 70 Kg; (3) body surface area; (4) body mass index.	Obesity
(1) Coronary artery disease; (2) ischemic heart disease; (3) prior myocardial infarction.	Coronary artery disease
(1) Arrhythmia; (2) atrial fibrillation/flutter.	Arrhythmia
(1) Cerebrovascular disease; (2) stroke; (3) transient ischemic attack.	CV disease
(1) Chronic obstructive pulmonary disease; (2) WHO performance status.	Other comorbidities
(1) Pre-existing CV disease; (2) endocarditis; (3) anticoagulant therapy	Other CV diseases
Using radionuclide ventriculography: (1) approximate entropy; (2) synchrony; (3) entropy; and (4) standard deviation from the phase histogram. Using echocardiography: (5) GLS; (6) mitral peak E-wave velocity; (7) LVESD; (8) diastolic dysfunction.	Other LV parameters
(1) Lymphocyte count; (2) neutrophils count; (3) creatinine; (4) hemoglobin.	Other blood variables*
(1) Anthracycline (dose); (2) anthracycline (yes/no); (3) baseline anthracycline exposure (dose); (4) doxorubicin vs pegylated liposomal doxorubicin; (5) epirubicin (dose); (6) number of cycles.	Anthracyclines
(1) Chest/mediastinal radiation (yes/no); (2) chest/mediastinal radiation (dose); (3) radiation to left breast; (4) mean heart dose.	Chest radiation
(1) Non-anthracycline based chemotherapy; (2) docetaxel/paclitaxel; (3) capecitabine; (4) gemcitabine; (5) bevacizumab; (6) cardiotoxic medication±; (7) trastuzumab; (8) 5-fluorouracil; (9) cyclophosphamide.	Other systemic drugs
(1) Cancer stage; (2) AML (vs ALL); (3) cancer metastasis; (4) tumor size; (5) pathology (invasive/non-invasive); (6) cancer type.	Cancer type and/or stage
(1) Follow-up duration; (2) use of dexrazoxane; (3) respiratory rate; (4) surgery.	Other§

ABPM, ambulatory blood pressure monitoring; ACEi/ARB, angiotensin-converting enzyme inhibitors/angiotensin receptor blockers; ALL, acute lymphoblastic leukemia; AML; acute myeloid leukemia; BP, blood pressure; CV, cardiovascular; ECG, electrocardiogram; GLS, global longitudinal strain; LV, left ventricle; LVEF, left ventricular ejection fraction; LVESD, left ventricular end-systolic diameter; WHO, World Health Organization.

*Most from machine learning modules containing > 300 variables[1], including multiple blood variables. ±Including anthracyclines, alkylating agents, Her2 inhibitors, microtubule binding agents, VEGF inhibitors, and BCR-ABL inhibitors. §Multiple other predictors included by machine learning models[1 2].

Supplemental Table 7. Outcome definitions across studies and their categorization

Author, year	Definition
	**COMPOSITE OUTCOME**
Abdel-Qadir H, 2019 [3]	Hospitalization for acute myocardial infarction, unstable angina, transient ischemic attack, stroke, peripheral vascular disease, HF, cardiovascular death (ICD-9/10 codes)
Abiodun A, 2024 [4]	New onset angina, acute coronary syndrome, new onset arrhythmia, new onset of symptoms or signs of HF, cardiac arrest (alternative cause unlikely), and cardiovascular death.
Advani PP, 2016 [5]	Symptomatic congestive HF, definite cardiac death (as a result of myocardial infarction, HF, or arrhythmia), or probable cardiac death (patient death without documented etiology).
Armenian S, 2018 [6]	HF, per established guidelines (ACC/AHA 2005), or coronary artery disease (myocardial infarction, symptomatic coronary artery stenosis requiring intervention).
Astarita A, 2021 [7]	All the events having a cardiovascular origin, including acute coronary syndrome, HF, arrhythmias, syncope, and chest pain, among others (graded according to CTCAE 5.0)

Battisti NML, 2021 [8]	Cardiac death, LVEF decline of ≥ 10%, LVEF decline to < 50%, congestive HF (NYHA class II-IV), and trastuzumab discontinuation due to cardiac toxicity.
Carballo-Folgoso L, 2021 [9]	HF, acute coronary syndrome, cerebrovascular accidents or peripheral ischemia.
Cronin M, 2023 [10]	Cardiovascular death, LVEF decline of ≥ 10%, LVEF decline to < 50%, congestive HF (NYHA class II-IV), and trastuzumab discontinuation due to cardiotoxicity.
Diamond A, 2022 [11]	Myocardial infarction, coronary artery disease, new congestive HF, asymptomatic decreased EF below 50%, stroke, peripheral artery disease, atrial fibrillation, other arrhythmias.
Fernando F, 2024 [12]	Any acute disease affecting the cardiovascular system, including acute coronary syndrome, cerebrovascular accidents, HF, and acute peripheral vascular disease, among others.
Heilbroner SP, 2021 [1]	First documentation of arrhythmia (atrial fibrillation, unspecified arrhythmia, premature depolarization, atrioventricular block, sick sinus syndrome, ventricular arrhythmia, cardiac arrest), HF, myocarditis, or pericardial disease (as defined by the MedDRA and ICD-9/10 codes).
Kim DY, 2021 [13]	Cardiovascular death, myocardial infarction, stroke/transient ischemic attack, and HF.
Law W, 2017 [14]	Cardiovascular events (HF- and cardiovascular disease-related hospitalizations and deaths) and cardiotoxicity (LVEF drop of > 10% to LVEF ≤ 50%).
Li C, 2022 [2]	Ischemic heart diseases, HF, cardiomyopathy, arrhythmia, acute myocarditis, stroke, cardiogenic shock, and sudden cardiac arrest (ICD-9-CM codes).
Liu B, 2022 [15]	Abnormal ECG (sinus arrhythmia, ST-T changes, low-voltage), abnormal echocardiography (reduction of LVEF or fractional shortening), increased cardiac biomarkers (NT-proBNP and troponin T).
McCracken C, 2024 [16]	Outcome 1: myocardial infarction, stroke, cardiovascular mortality, incident HF, atrial fibrillation, non-ischemic cardiomyopathies, and valvular heart disease. Outcome 2: HF alone.
Mery B, 2022 [17]	Cardiovascular adverse events (scored according to CTCAE 3.30), including atrial fibrillation, HF, myocardial infarction, stroke/transitory ischemic attack, and venous thromboembolism, among others.
Nguyen QTN, 2024 [18]	Myocardial infarction, arrhythmia, conduction disorders, HF, and coronary artery diseases (ICD-9/10 codes).
Romond EH, 2012 [19]	Definite or probable cardiac death or congestive HF manifested by dyspnea with normal activity or at rest and associated with an absolute decrease in LVEF of > 10% from baseline to < 55% or a decrease of > 5% to a value below the lower limit of normal.
Shibata T, 2023 [20]	HF/left ventricular systolic dysfunction, acute coronary syndrome, venous thromboembolism, new arterial hypertension, atrial fibrillation, bradycardia, QT corrected interval prolongation, and pericardial effusion (according to CTCAE 4.0).
Stefanini B, 2024 [21]	HF, acute coronary syndrome, cerebrovascular accidents, or peripheral ischemia.
Tini G, 2022 [22]	Left ventricular dysfunction (according to the American Society of Echocardiography), other cardiovascular events (pulmonary embolism, uncontrolled arterial hypertension, HF with preserved EF, cardiac tamponade, fatal ischemic stroke, and symptomatic ectopic ventricular beats).
Vasbinder A, 2024 [23]	Cardiovascular death, nonfatal myocardial infarction, stroke, new-onset HF, a new diagnosis of atrial fibrillation or flutter, and sustained ventricular tachycardia.
Yuan S, 2023 [24]	Cardiovascular events (CTCAE v5.0 any grade), including HF, arrhythmia (premature beats, atrial fibrillation, paroxysmal supraventricular tachycardia), hypertension, acute coronary syndrome, pulmonary hypertension, pericardial effusion.
**CTRCD**	
Caro-Codón J, 2022 [25]	Cardiotoxicity, defined as a new or worsening myocardial damage/dysfunction (symptomatic or asymptomatic).
Chaix MA, 2020 [26]	Decrease in LVEF to an absolute value < 50% or decrease in LVEF of > 10% to an absolute value < 55%.
Chang WT, 2022 [27]	Outcome 1: CTRCD, defined as a decrease in LVEF of > 10% to an absolute value <50%. Outcome 2: HF with reduced ejection fraction (LVEF < 40%)
Chen Y, 2020 [28]	HF, according to the CTCAE (version 4.03) grades 3-5: cardiomyopathy requiring medication; HF requiring transplant; or fatal HF.

Chow E, 2015 [29]	HF, according to the CTCAE (version 4.03) grades 3-5: cardiomyopathy requiring medication; HF requiring transplant; or fatal HF.
De Vries S, 2023 [30]	The first HF event, according to the CTCAE (version 4.0) grades 3-5, without other prior cardiovascular events.
Di Lisi D, 2024 [31]	Symptomatic or asymptomatic CTRCD, according to the 2022 ESC Cardio-Oncology guidelines.
Doukas PG, 2022 [32]	The definition of HF was based on the 2017 Cardiovascular and Stroke Endpoint Definitions for Clinical Trials, including both symptoms and physical exam findings.<br>HF events were tracked when patients presented to a hospital setting and required inpatient admission for treatment of their symptoms. Outpatient evaluations were not considered.
Dranitsaris G, 2008 [33]	Cardiac toxicity, defined as: (1) decrease in LVEF of ≥ 20% but remaining in the normal range; (2) decrease in LVEF of ≥ 10% to below the lower limits of normal; or (3) clinical signs and/or symptoms of HF.
Ezaz G, 2014 [34]	HF or cardiomyopathy, defined by ICD-9-CM codes that appeared in at least one inpatient claim or 2 outpatient claims at least 30 days apart.
Fogarassy G, 2019 [35]	HF following the start of chemotherapy, defined as: (1) hospital discharge from specific departments following the diagnosis of the ICD code I50; (2) hospitalization that ended in death and an I50 code issued as a primary or secondary diagnosis or as the underlying (not immediate) cause of death; and (3) autopsy report with the I50 code (excluding immediate cause of death).
Gomez-Vecino A, 2023 [36]	HF, according to the CTCAE (version 4.0) grades 3-5 or asymptomatic decrease in LVEF of > 10% [37].
Gunturkun F, 2021 [38]	LVEF < 50% or decrease in LVEF of ≥ 10%.
Jacobs J, 2022 [39]	Cardiotoxicity, according to the CREC criteria: (1) decrease in LVEF of ≥ 10% in asymptomatic patients to an absolute value < 55%; or (2) decrease in LVEF of ≥ 5% in symptomatic patients to an absolute value < 55%.
Jones KA, 2020 [40]	Decrease in LVEF of > 10% to an absolute value < 50%.
Kang Y, 2019 [41]	HF was confirmed if the patient had at least one of the new-onset or worsening HF symptoms as well as one of: (1) at least two physical examination findings; and (2) at least one relevant HF therapy.<br>The definition of HF was based on the 2017 Cardiovascular and Stroke Endpoint Definitions for Clinical Trials.
Kotwinski P, 2016 [42]	Cardiotoxicity, defined as: (1) decrease in LVEF ≥ 5% from baseline (subclinical cardiotoxicity); (2) diagnosis of HF by the clinical team, or a subclinical fall in LVEF ≥ 10% to below normal (overt cardiotoxicity).
Leerink JM, 2021 [43]	Left ventricular dysfunction with ejection fraction < 40%.
Liu X, 2022 [44]	Decrease in LVEF of ≥ 10% from baseline.
Liu Z, 2022 [45]	Cardiac toxicity, defined as: (1) decrease in LVEF of ≥ 20% but remaining in the normal range; (2) decrease in LVEF of ≥10% to below the lower limits of normal; or (3) clinical signs and/or symptoms of HF.
Moey MYY, 2019 [46]	Decrease in LVEF of ≥ 15%.
Oikonomou EK, 2024 [47]	CTRCD, defined as: combination of diagnosis codes for cardiomyopathy/heart failure and/or documentation of left ventricular systolic dysfunction (< 50%).
Otchere P, 2023 [48]	Decrease in LVEF of > 10% to an absolute value < 53%.
Ozturk C, 2021 [49]	Decrease in LVEF of ≥ 10% to an absolute value < 50% or decrease in GLS ≥ 15% from baseline.
Pohl J, 2021 [50]	Decrease in LVEF of ≥ 10% to an absolute value < 50% or decrease in GLS ≥ 15% from baseline.
Rivero-Santana B, 2024 [51]	Symptomatic or moderate to severe asymptomatic CTRCD, according to the 2022 ESC guidelines on cardio-oncology.
Rushton M, 2017 [52]	Primary endpoint: permanent HF or cardiomyopathy. A cardiac event was defined as: decrease in LVEF ≥ 10% to an absolute value < 50%.
Sun Y, 2022 [53]	Cardiotoxicity, defined as: (1) decrease in LVEF of ≥ 5% to an absolute value < 55% accompanied by symptoms of HF; or (2) decrease in LVEF of ≥ 10% to an absolute value < 55%, without HF symptoms or signs; or (3) definite clinical diagnosis of HF.

Suntheralingham S, 2022 [54]	CTRCD, defined as: (1) decrease in LVEF of ≥ 10% to an absolute value < 55% without symptoms; or (2) decrease in LVEF of ≥ 5% to an absolute value of < 55% with HF symptoms.
Upshaw JN, 2019 [55]	Cardiotoxicity, defined as a decrease in LVEF of ≥ 10% to an absolute value < 50% and/or clinical diagnosis of HF.
Yagi R, 2024 [56]	Decrease in LVEF of > 10% to an absolute value < 53%.
Yu AF, 2023 [57]	Decrease in LVEF of ≥ 10% to an absolute value < 53% or decrease in LVEF of ≥ 16% from baseline.

ACC/AHA, American College of Cardiology/American Heart Association; CREC, Cardiac Review and Evaluation Committee; CTCAE, common terminology criteria for adverse events; CTRCD, cancer therapy-related cardiac dysfunction; ECG, electrocardiogram; EF, ejection fraction; ESC, European Society of Cardiology; GLS, global longitudinal strain; HF, heart failure; ICD, international classification of diseases; LVEF, left ventricular ejection fraction; MedDRA, Medical Dictionary for Regulatory Activities; NT-proBNP, N-terminal pro-B-type natriuretic peptide; NYHA, New York Heart Association.

Supplemental Table 8. Observed outcomes across HFA-ICOS external validation studies, categorized by clinical relevance.

Author (PMID)	Model	Outcome definition	Total sample	Events (n)*	Observed CTRCD (n)	Other CV events± (n)
Studies targeting CTRCD, where moderate to severe (or symptomatic) CTRCD† events were predominant						
Cronin M, 2023[10]	HFA-ICOS [anti-Her2]	1) Decline in LVEF ≥10%; 2) Decline in LVEF to < 50%; 3) Discontinuation of trastuzumab therapy due to cardiotoxicity; 4) Development of congestive HF (NYHA class II-IV); 5) Death due to CV event.	507	23§	LVEF decline to < 50% (n=14), HF (n=9), and death due to cardiotoxicity (n=2) ¶	-
Rivero-Santana B, 2024[51]	HFA-ICOS [anthracyclines]	Symptomatic CTRCD or severe/moderate asymptomatic CTRCD, according to the 2022 ESC definition[58] †	1,066	69	LVEF decline to < 50% (n=69)	-
Studies targeting CTRCD, where mild asymptomatic CTRCD† events were predominant						
Battisti NML, 2021[8]	HFA-ICOS [anti-Her2]	1) Death due to cardiac reasons; 2) LVEF decline ≥ 10%; 3) LVEF decline to < 50%; 4) HF (NYHA II-IV); 5) Trastuzumab discontinuation due to cardiac toxicity	931	155*	LVEF decline ≥10% (n=141); LVEF decline to < 50% (n=55); HF (n=47); discontinuation due to cardiac toxicity (n=35) ¶	-
Di Lisi D, 2024[31]	HFA-ICOS [anthracyclines]	CTRCD defined according to the 2022 ESC criteria[58] on the basis of changes in LVEF and GLS†	109	26	Not clear ¶ ‡	-
Liu X, 2022[44]	HFA-ICOS [anti-Her2]	LVEF decline of ≥ 10% from baseline	212	72	LVEF decline ≥10% (n=72); LVEF decline to < 50% (n=3) ¶	-
Suntheralingam S, 2022[54]	HFA-ICOS [anti-Her2]	CTRCD based on CREC criteria: LVEF decline ≥ 10% to < 55% without symptoms or LVEF decline ≥5% to <55% with symptoms ±±	629	151	LVEF decline to < 55% (n=151); LVEF decline to < 50% (n=35) ¶	-
Studies including other CV events, beyond CTRCD, in a composite endpoint						
Fernando F, 2024[12]	HFA-ICOS [BCR-ABL]	Cardiovascular events including: ACS, cerebrovascular accidents, HF, and acute peripheral vascular disease, among others **	229	48	HF (n=2)	ACS (n=14), peripheral vascular disease (n=12), hypertension (n=9), arrhythmias (n=5), stroke (n=4), VTE (n=1), and syncope (n=1)

Shibata T, 2022[20]	HFA-ICOS [non specified]	CV adverse events, according to the CTCAE version 4, grouped as: HF/LVSD; ACS; VTE; new arterial hypertension; AF; bradycardia; QTc interval prolongation; and pericardial effusion **	486	97	HF/LVSD (n=41)	QT prolongation (n=28), VTE (n=13), hypertension (n=10), atrial fibrillation (n=8), bradicardia (n=8), pericardial effusion (n=7), and ACS (n=1).
Stefanini B, 2024[21]	HFA-ICOS [VEGF inhibitors]	MACE (HF, ACS, cerebrovascular accidents, or peripheral ischemia)	843	34	HF (n=7)	Cerebrovascular accidents (n=15), and ACS (n=12).
Tini G, 2022[22]	HFA-ICOS [anti-Her2]	1) Left ventricular dysfunction, according to the 2014 ASE statement (LVEF decline > 10% to <53%)[59]; 2) other CV events (not specified)	171	21	LVEF decline > 10% to <53% (n=16)	Hypertension (n=2), cardiac tamponade (n=1), stroke (n=1), and ectopic ventricular beats (n=1).
Tini G, 2022[22]	HFA-ICOS [anthracyclines]	1) Left ventricular dysfunction, according to the 2014 ASE statement (LVEF decline > 10% to <53%)[59] 2) other CV events (not specified)	202	4	HF (n=1)	Pulmonary embolism (n=2), and hypertension (n=1)

ACS, acute coronary syndrome; CREC, Cardiac Review and Evaluation Committee; CTCAE, Common Terminology Criteria for Adverse Events; CV, cardiovascular; ESC, European Society of Cardiology; HF, heart failure; HFA-ICOS, Heart Failure Association – International Cardio-Oncology Society; LVEF, left ventricular ejection fraction; LVSD, left ventricular systolic dysfunction.

*Number of participants with the event; may not correspond to the number of events described under ("Observed CTRCD" and "Other CV events"), as some studies report more than one outcome per participant. Often the reporting was poor and outcomes not clearly defined to allow perfect classification. ±Other CV events apply to studies using composite outcomes, including heart failure and other CV events. We have classified them into major or minor according to clinical relevance. §Number of events considered to be related to cancer treatments. ¶More than one reported event per patient. †ESC definition of asymptomatic CTRCD: mild (LVEF ≥ 50% and new decline in GLS > 15% or new rise in cardiac biomarkers), moderate (LVEF decline ≥ 10% to 40-49% or LVEF decline < 10% to 40-49% and new decline in GLS > 15% or new rise in cardiac biomarkers), and severe (LVEF decline to < 40%) CTRCD[58]. ‡The number of participants experiencing each type of event (mild CTRCD, ≥ moderate CTRCD, or heart failure) is not clear, but mild events seem to be predominant. ** Acute coronary syndrome defined according to the 2023 ESC guidelines; cerebrovascular accidents defined according to the AHA/ASA 2021 guidelines; acute peripheral vascular disease defined as new onset of intermittent claudication or acute vascular occlusion with the need for interventional treatment. ±±Sensitivity analyses were conducted using the 2016 ESC definition[60] and the 2014 ASE statement[59].

Supplemental Table 9. List of articles that were excluded after full-text assessment despite presenting a prediction model or performing multivariable modeling

Author, year	Reasons for exclusion
Alenezi A, 2024[61]	Exploratory analyses, no prediction model proposed for use in clinical practice.
Ali MT, 2016[62]	Prognostic factor study: baseline GLS.
Araujo-Gutierrez R, 2021[63]	Prognostic factor study: baseline GLS.
Armenian SH, 2013[64]	Prognostic factor study: genetic polymorphisms.
Bergamini C, 2024[65]	Prognostic factor study: early left atrial functional decline.
Bottinor WJ, 2023[66]	Longitudinal predictors: longitudinal strain and circumferential strain.
Cai G, 2022[67]	Focus on radiation-induced cardiotoxicity.
Calvillo-Arguëlles O, 2022 [68]	Prognostic factor study: changes in GLS and myocardial work indices.
Choe JC, 2018[69]	Prognostic factor study: prolonged electromechanical delay.
De Baat EC, 2024[70]	Diagnostic prediction model.
De Barros MVL, 2019[71]	Prognostic factor study: left ventricular regional wall motion abnormality.
Demissei BG, 2020[72]	Prognostic factor study: change in biomarkers (e.g., NT-proBNP, myeloperoxidase).
Demissei BG, 2021[73]	Prognostic factor study: left ventricular segmental strain.
Ehrhardt MJ, 2024[74]	Prognostic factor study: GLS and NT-proBNP.
Fawzy A, 2024[75]	Prognostic factor study: right and left ventricular strain measurements.
Goldberg JF, 2021[76]	Prognostic factor study: family history of cardiovascular disease.
Hahn E, 2017[77]	Focus on radiation-induced cardiotoxicity.
Hathaway QA, 2024[78]	Prognostic factor study: ultrasonic features.
Hochstadt A, 2020[79]	Prognostic factor study: longitudinal diastolic strain slope.
Hou Y, 2021[80]	Longitudinal predictors: machine learning model using data from up to 20 years' follow-up.
Houbois CP, 2021[81]	Prognostic factor study: changes in cardiac magnetic resonance and echocardiographic parameters.
Inoue K, 2024[82]	Longitudinal predictors: cardiac biomarkers and echocardiographic parameters.
Jacobs J, 2024[83]	Diagnostic prediction model.
Ladbury C, 2023[84]	Focus on radiation-induced cardiotoxicity.
Leerink JM, 2024[85]	Diagnostic prediction model.
Leger KJ, 2016[86]	Prognostic factor study: genetic markers.
Ma Y, 2013[87]	Prognostic factor study: ischemia-modified albumin.
Milks MW, 2018[88]	Prognostic factor study : change in GLS and LVEF.
Mousavi N, 2015[89]	Prognostic factor study: echocardiographic parameters.
Narayan HK, 2016[90]	Prognostic factor study: strain measures.
Oikawa M, 2021[91]	Prognostic factor study: D-dimer.
Posch F, 2022[92]	Two predictors, including one longitudinal predictor.
Terluk A, 2024[93]	Prognostic factor study: baseline GLS.
Tian C, 2024[94]	Prognostic factor study: GDF-15.
Tjong MC, 2022[95]	Focus on radiation-induced cardiotoxicity.

Tlegenova Z, 2023[96]	Predictors measured 9 months after start of treatment: 6 min walk test, B-type natriuretic peptide, and LVEF.
Tu C, 2024[97]	Prognostic factor study: coronary computed tomography parameters.
Usendia C, 2023[98]	Prognostic factor study: left ventricle strain measurements.
Visscher H, 2012[99]	Exploratory study with focus on genetic markers, case-control design.
Visscher H, 2015[100]	Exploratory study with focus on genetic markers, case-control design.
Yaegashi D, 2020[101]	Prognostic factor study: red blood cell distribution width.
Zhou Y, 2020[102]	Exploratory study, use of multiple machine learning techniques to develop multiple different models. The authors don't propose any model and the main focus of the study is on evaluating different machine learning methods rather than the development of a prediction model for use in clinical practice.

GDF-15, growth differentiation factor-15; GLS, global longitudinal strain; LVEF, left ventricular ejection fraction; NT-proBNP, N-terminal pro-B-type natriuretic peptide.

Supplemental Table 10. Detailed characteristics of included development and external validation studies

Author, year	Model	Study design (data source)	Location	Sample size	Age*	Female	Cancer type	Main treatment	T0	Prediction horizon	Outcome±	Events	EPV ratio§	Modelling method	Model presentation
**CHILDREN, ADOLESCENTS AND YOUNG ADULTS**															
Development studies															
Chaix MA, 2020	a) [clinical]<br>b) [genetic]<br>c) [both]	Nested case-control	USA, multicentre	289	Median ~ 5	51%	Leukemia 46%, neuroblastoma 11%, other¶.	Anthracycline-based chemotherapy	≥ 3 yrs from their last anthracycline	Not specified	LVEF decline	183	a) 26.1<br>b) 5.9<br>c) 4.8	Random forest	None
Chen Y, 2020	a) [age 20]<br>b) [age 25]<br>c) [age 30]<br>d) [age 35]	Cohort (CCSS)	USA + Canada	41,689	Median 5 - 15	47%	Leukemia, lymphoma, CNS tumor, bone tumor, other.	Chemotherapy †	20 yrs (age)<br>25 yrs (age)<br>30 yrs (age)<br>35 yrs (age)	50 yrs (age)	HF	a) 169<br>b) 194<br>c) 128<br>d) 84	a) 16.9<br>b) 17.6<br>c) 14.2<br>d) 10.5	Weilbull regression	Sum score + online calculator
De Vries S, 2023	a) [simple]<br>b) [dose]	Cohort	The Netherlands	1,433	30 (24-38)	60%	Hodgkin lymphoma	Chemotherapy	5 yrs after initial HL treatment	20 yrs + 30 yrs (after treatment)	HF	102	a) 20.4<br>b) 14.6	Cox regression	Risk score table + online calculator + full regression formula
Gunturkun F, 2021	a) [clinical]<br>b) [ECG]<br>c) [both]	Cohort (SJLIFE)	USA	1,217	Median 8.4	49%	Leukemia, sarcomas, lymphomas, CNS tumors, other.	Anthracycline-based chemotherapy	22 yrs after cancer diagnosis	Not specified	LVEF decline	117	a) 16.7<br>b) 1.4<br>c) 1.3	XGboost‡	None
Liu Z, 2022	Liu Z 2022	Cohort	China	796	Median ≤ 5	39%	ALL, AML, NHL.	Anthracycline-based chemotherapy	Start of treatment	1 year	HF / LVEF decline	64	16	Logistic regression	Nomogram
Development + External validation															
Chow E, 2015	a) [simple]<br>b) [standard]<br>c) [heart dose]	Cohort (CCSS) [D]	USA + Canada	13,060	Median 12	47%	ALL, Hodgkin lymphoma, brain tumors, other.	Chemotherapy	5 yrs after diagnosis	40 yrs (age)	HF	285	a) 71.2<br>b) 35.6<br>c) 31.7	Poisson + Cox regression	Sum score

	a) [simple]<br>b) [standard]<br>c) [heart dose]	Cohort (EKZ/AMC) [V]	The Netherlands	1,362	Median 5 - 10	45%	ALL, NHL, brain tumors, kidney tumors, other.	Chemotherapy	5 yrs after diagnosis	40 yrs (age)	HF	26	a) 6.5<br>b) 3.2<br>c) 2.9		
	a) [simple]<br>b) [standard]	Case-cohort (NWTS) [V]	USA + Canada	364	Median < 5	55%	Kidney tumors	Chemotherapy	5 yrs after diagnosis	40 yrs (age)	HF	48	a) 12<br>b) 6		
	a) [simple]<br>b) [standard]	Cohort (SJLIFE) [V]	USA	1,695	Median 5 - 10	48%	ALL, Hodgkin lymphomas, brain tumors, other.	Chemotherapy	10 yrs after diagnosis	40 yrs (age)	HF	19	a) 4.7<br>b) 2.4		
Leerink JM, 2021	Leerink 2021	Cohort (EKZ/AMC) [D]	The Netherlands	299	7 (4-12)	56%	NHL, ALL, nephroblastoma, soft-tissue sarcoma, other.	Anthracycline-based chemotherapy	≥5 yrs after diagnosis	10 yrs	LVEF decline	11	3.7	Cox regression	Online calculator
		Cohort [V]	The Netherlands	218	7 (4-12)	50%	Leukemias, lymphomas, nephroblastoma, other.	Anthracycline-based chemotherapy	≥5 yrs after diagnosis	10 yrs	LVEF decline	7	2.3		
**ADULTS**															
Development studies															
Abdel-Qadir H, 2019	Abdel-Qadir 2019	Cohort (administrative + registry data)	Canada	90,104	61 (51-71)	100%	Breast cancer	Chemotherapy	Cancer diagnosis	5 yrs + 10 yrs	Composite outcome	6,772	376.2	Fine-Gray method	Sum score
Astarita A, 2021	Astarita 2021	Cohort	Italy	116	Median 64 ± 8	44%	Multiple myeloma	Carfilzomib	Start of treatment	Not specified	Composite outcome	52	10.4	Logistic regression	Full regression formula
Carballo-Folgoso L, 2021	CARDIOSOR scale	Cohort	Spain	299	66 (59-72)	14%	Hepatocellular carcinoma	Sorafenib	Start of treatment	Not specified	Composite outcome	33	6.6	Cox regression	Sum score
Chang WT, 2022	a) [CTRCD]<br>b) [HFrEF]	Cohort	Taiwan	211	56 ± 10	100%	Breast cancer	Anthracycline-based chemotherapy	Start of treatment	3 yrs	HF / LVEF decline	a) 23<br>b) 8	a) 1.5<br>b) 0.5	Logistic regression‡	None
Diamond A, 2022	Diamond 2021	Cohort	USA	463	63	45%	Diffuse large B cell lymphoma	Anthracycline-based chemotherapy	Cancer diagnosis	Not specified	Composite outcome	92	23	Logistic regression	Sum score + online calculator

Dranitsaris G, 2008	Dranitsaris 2008	Clinical trial	International	509	Mean 58	100%	Breast cancer	Anthracycline-based chemotherapy	Start of treatment	Not specified	HF / LVEF decline	58	8.3	Cox regression	Sum score
Ezaz G, 2014	Ezaz 2014	Cohort (SEER-Medicare)	USA	1,664	74 ± 5	100%	Breast cancer	Chemotherapy	Cancer diagnosis	3 yrs	HF	318	35.3	Logistic regression	Sum score
Fogarassy G, 2019	Fogarassy 2019	Cohort (national registries)	Hungary	8,068	50 - 60	99%	Breast cancer	Anthracycline-based chemotherapy	Start of treatment	Not specified	HF	557	29.3	Logistic regression	Sum score
Gomez-Vecino A, 2023	Gomez-Vecino 2023	Cohort	Belgium	420	50 ± 9	100%	Breast cancer	Anthracycline-based chemotherapy	Cancer diagnosis	Not specified	HF / LVEF decline	NI	NI	Cox regression	None
Heilbroner SP, 2021	a) [full]<br>b) [simplified]	Cohort (CancerLinQ)	USA	3,229	64 ± 11	46%	Lung cancer, melanoma, renal cell carcinoma.	Immune checkpoint inhibitors	Start of treatment	Not specified	Composite outcome	418	a) 1.2<br>b) 20.9	XGboost	None
Jones KA, 2020	a) Jones 2020 [1]<br>b) Jones 2020 [2]<br>c) Jones 2020 [RF]	Cohort	UK	177	Mean 54	100%	Breast cancer	Unspecified	Start of treatment	Not specified	LVEF decline	11	a) 1.8<br>b) 3.7<br>c) 1.8	a/b) Logistic regression<br>c) Random forest‡	a/b) Coefficients but no intercept<br><br>c) None
Kang Y, 2019	Kang 2019	Cohort	USA	450	51 ± 15	52%	Acute leukemia	Anthracycline-based chemotherapy	Start of treatment	Not specified	HF	40	6.7	Fine-Gray method	Sum score
Kim DY, 2021	CHEMO-RADIAT	Cohort	South Korea	1256	51 ± 11	100%	Breast cancer	Anthracycline-based chemotherapy	Cancer diagnosis	1-, 3- and 7-yrs	Composite outcome	21	1.9	Cox regression	Sum score
Kotwinski P, 2016	Kotwinski 2016	Cohort	UK	165	48 ± 9	100%	Breast cancer	Anthracycline-based chemotherapy	Start of treatment	Not specified	HF / LVEF decline	34	8.5	Logistic regression	Full regression formula
Li C, 2022	Li 2022 [XGBoost]	Cohort (SEER-Medicare)	USA	36,030	NI	NI	Colorectal	Fluoropyrimidine-based chemotherapy	Start of treatment	30 days	Composite outcome	NI	NI	XGboost‡	None
Liu B, 2022	Liu B 2022	Cohort	China	388	50 ± 10	100%	Breast cancer	Anthracycline-based chemotherapy	Start of treatment	Not specified	Composite outcome	180	60	Logistic regression	Sum score

Otchere P, 2023	Otchere 2023	Cohort	USA	415	58 ± 13	100%	Breast cancer	Her2 inhibitors	Start of treatment	1 yr	LVEF decline	39	4.9	Logistic regression	Coefficients but no intercept
Ozturk C, 2021	Ozturk 2021	Cohort	Germany	225	Median 58 ± 6	53%	Breast cancer, hematological malignancies, gastrointestinal cancer, other.	Chemotherapy and targeted therapies	Start of treatment	Not specified	LVEF / GLS decline	36	5.1	Logistic regression	Sum score
Romond EH, 2012	Romond 2012	Clinical trial	USA	944	Mean 49	100%	Breast cancer	Her2 inhibitors	Start of treatment	5 yrs	Composite outcome	47	23.5	Cause-specific hazard model	Full regression formula
Sun Y, 2022	Sun 2022	Cohort	China	257	< 65	100%	Breast cancer	Anthracycline-based chemotherapy	Start of treatment	Not specified	HF / LVEF decline	32	8	Logistic regression	Sum score + full regression formula
Upshaw JN, 2019	Upshaw 2019	Clinical trial	USA	967	51 ± 10	100%	Breast cancer	Anthracycline-based chemotherapy	Start of treatment	1 yr	HF / LVEF decline	51	17	Logistic regression	Full regression formula
Yagi R, 2024	Yagi 2024 [Model 1]	Cohort	USA	Unclear	Unclear	Unclear	Hematological malignancies, breast cancer, other.	Anthracycline-based chemotherapy	Start of treatment	0.5, 1, 1.5 and 2 yrs	LVEF decline	Unclear	Unclear	Cox regression	Coefficients but no intercept
Yu AF, 2023	Yu 2024	Cohort	USA	1,377	52 ± 11	100%	Breast cancer	Her2 inhibitors	Start of treatment	1 yr	LVEF decline	177	19.7	Cox regression	Nomogram
Yuan S, 2023	Yuan 2023	Cohort	China	253	63 (56-68)	60%	Multiple myeloma	Proteasome inhibitors, IMiDs	Start of treatment	1 yr + 2 yrs	Composite outcome	74	24.7	Cox regression	Sum score
Development + External validation															
Armenian SH, 2018	Armenian 2018 [full model]	Cohort [D]	USA	1,828	45 (2-80)	42%	HCT survivors: leukemias, lymphomas, other.	Anthracycline-based chemotherapy	1 yr after HCT	10 yrs	Composite outcome	135	19.3	Fine-Gray method	Sum score
		Case-cohort (FHCRC) [V]	USA	580	45 (2-74)	45%	HCT survivors: leukemias, lymphomas, other.	Chemotherapy	1 yr after HCT	10 yrs	Composite outcome	155	22.1		

Jacobs J, 2022	a) Jacobs 2022 [1]<br>b) Jacobs 2022 [2]	Cohort [D]	Belgium	324	63 ± 16	68%	Breast cancer, hematological malignancies, other.	Chemotherapy	Start of treatment	Not specified	HF / LVEF decline	104	a) 17.3<br>b) 14.9	Logistic regression	None
	c) ASCO score<br>d) CRS (Herrmann, 2014)	Cohort [V]	Belgium	324	63 ± 16	68%	Breast cancer, hematological malignancies, other.	Chemotherapy	Start of treatment	Not specified	HF / LVEF decline	104	c) 14.9<br>d) 9.4		
Liu X, 2022	ABSDELL model	Cohort [D]	China	212	53 ± 12	100%	Breast	Her2 inhibitors	Start of treatment	1 yr + 3 yrs	LVEF decline	72	10.3	Cox regression	Nomogram + online calculator
	HFA-ICOS [anti-Her2]	Cohort [V]	China	212	53 ± 12	100%	Breast	Her2 inhibitors	Start of treatment	3 yrs	LVEF decline	72	3.6		
Nguyen QT, 2024	Nguyen 2024	Cohort [D]	Taiwan	1,321	56 ± 11	100%	Breast	Anthracyclines +/- Her2 inhibitors	Start of treatment	1 yr	Composite outcome	112	NI	Artificial neural network (ANN)	None
		Cohort [V]	Taiwan	NI	NI	100%	Breast	Anthracyclines +/- Her2 inhibitors	Start of treatment	1 yr	Composite outcome	NI	NI		
Vasbinder A, 2024	CARE-BMT risk score	Cohort [D]	USA	2,435	55 ± 13	40%	HCT for malignant and non-malignant disorders	Unspecified	Before or at the time of HCT	100 days, 1 yr, 5 yrs, 10 yrs	Composite outcome	333	30.3	Random survival forest	Sum score
		Cohort [V]	USA	770	54 ± 13	42%	HCT for malignant and non-malignant disorders	Unspecified	Before or at the time of HCT	100 days, 1 yr, 5 yrs, 10 yrs	Composite outcome	112	10.2		
	Armenian 2018 [full model]	Cohort [V]	USA	2,435	55 ± 13	40%	HCT for malignant and non-malignant disorders	Unspecified	Before or at the time of HCT	10 yrs	HF + myocardial infarction	204	29.1		
	HCT-CI	Cohort [V]	USA	2,435	55 ± 13	40%	HCT for malignant and non-malignant disorders	Unspecified	Before or at the time of HCT	10 yrs	Composite outcome	333	19.6		

External validation													
Abiodun A, 2024	QRISK3	Cohort	UK	1,898	64 (54-72)	55%	Colorectal cancer 49%, breast cancer 22%, other.	Fluoropyrimidine-based chemotherapy	Start of treatment	3 months after last chemo	Composite outcome	59	1.8
Advani P, 2016	Romond 2012	Clinical trial	USA	1944	Median 49	100%	Breast cancer	Anthracyclines +/- Her2 inhibitors	During treatment	Not specified	Composite outcome	49	24.5
Battisti NML, 2021	HFA-ICOS [anti-Her2]	Cohort	UK	931	54 (46-63)	100%	Breast cancer	Her2 inhibitors	Cancer diagnosis	Not specified	Composite outcome	155	7.7
Caro-Codón J, 2022	SCORE	Cohort (CARDIOTOX registry)	Spain	1,287	55 ± 14	80.5%	Breast cancer 60%, NHL 15%, other.	Anthracycline-based chemotherapy	Start of treatment	2 yrs	HF / LVEF decline	341	26.2
Cronin M, 2023	HFA-ICOS [anti-Her2]	Cohort	Ireland	507	< 65	100%	Breast cancer	Her2 inhibitors	Start of treatment	Not specified	Composite outcome	23	1.1
Di Lisi D, 2024	HFA-ICOS [anthracycline]	Cohort	Italy	109	56 ± 11	100%	Breast cancer	Anthracycline-based chemotherapy	Cancer diagnosis	Not specified	Composite outcome	26	1.4
Doukas PG, 2022	Kang 2019	Cohort	USA	193	54 ± 14	44%	AML	Anthracycline-based chemotherapy	Start of treatment	1 yr	HF	34	5.7
Fernando F, 2024	HFA-ICOS [BCR-ABL]	Cohort	UK	229	Median 49	50%	Chronic myeloid leukemia	Nilotinib	Start of treatment	Not specified	Composite outcome	48	2.3
Law Q, 2017	Framingham 2008	Cohort	Canada	152	Median 57	97%	Breast cancer	Her2 inhibitors	Start of treatment	10 yrs	Composite outcome	22	3.1
Mccracken C, 2024	a) QRISK3<br>b) Framingham 2008 (BMI)<br>c) Framingham 2008<br>d) SCORE2 / SCORE-OP<br>e) PCP-HF	Cohort (UK Biobank)	UK	31,534	59 ± 7	66%	Breast cancer, prostate cancer, hematological malignancies, lung cancer, brain cancer, other.	NI	Cancer diagnosis	10 yrs	a/b/c/d) Composite outcome<br>e) HF	3636<br>789	a) 114<br>b) 606<br>c) 519<br>d) 330<br>e) 65.7

Mery B, 2022	Abdel-Qadir 2019	Cohort	France	943	Mean 57	99%	Breast cancer	Anthracycline-based chemotherapy	Cancer diagnosis	5 yrs + 10 yrs	Composite outcome	83	4.6
Moey MYY, 2019	Ezaz 2014	Cohort	USA	127	57 ± 11	100%	Breast cancer	Her2 inhibitors	Start of treatment	Not specified	LVEF decline	13	1.4
Oikonomou EK, 2024	AI-ECG (Sangha, 2023)	Cohort	USA	1,308	59 (49-67)	76%	Breast cancer, NHL.	Anthracycline-based chemotherapy	Start of treatment	24 months	HF / LVEF decline	404	NI
Pohl J, 2021	ECG score (Chatterjee, 2020)	Cohort	Germany	134	62 ± 15	68%	Skin cancer 48%, breast cancer 22%, other.	Chemotherapy and targeted therapies	Start of treatment	Not specified	LVEF / GLS decline	28	5.6
Rivero-Santana B, 2024	HFA-ICOS [anthracycline]	Cohort (CARDIOTOX registry)	Spain	1,066	54 ± 14	82%	Breast cancer 64%, NHL 17%, other.	Anthracycline-based chemotherapy	Start of treatment	12 months	HF / LVEF decline	69	3.8
Rushton M, 2017	Ezaz 2014	Cohort	Canada	138	56 ± 12	97%	Breast cancer	Her2 inhibitors	During treatment	3 yrs	HF / LVEF decline	62	6.9
Shibata T, 2023	HFA-ICOS [unspecified]	Cohort	Japan	486	64 ± 13	53%	Hematological malignancies 82%, breast cancer 18%.	Chemotherapy and targeted therapies	Start of treatment	Not specified	Composite outcome	97	4.8
Stefanini B, 2024	a) CARDIOSOR<br>b) HFA-ICOS [VEGF inhibitors]	Cohort (national registries)	Italy	843	68 (60-74)	17%	Hepatocellular carcinoma	Sorafenib	Start of treatment	Not specified	Composite outcome	34	6.8<br>1.5
Suntheralingam S, 2022	a) HFA-ICOS [anti-Her2]<br>b) Ezaz 2014<br>c) Romond 2012	Cohort	Canada	629	52 ± 11	100%	Breast cancer	Her2 inhibitors	Start of treatment	Not specified	HF / LVEF decline	151	a) 7.5<br>b) 16.8<br>c) 75.5
Tini G, 2022	HFA-ICOS [anti-Her2]	Cohort	Italy	171	61 ± 13	100	Breast cancer	Anthracycline-based chemotherapy	Start of treatment	Not specified	Composite outcome	21	1.0
	HFA-ICOS [anthracycline]	Cohort	Italy	202	58 ± 11	100	Breast cancer	Her2 inhibitors	Start of treatment	Not specified	Composite outcome	4	0.2

AI-ECG, Artificial Intelligence-enhanced Electrocardiography; ALL, acute lymphoblastic leukemia; AML, acute myeloid leukemia; ASCO, American Society of Clinical Oncology; BMI, body mass index; CARE-BMT, Cardiovascular Registry in Bone Marrow Transplantation; CCSS, Childhood Cancer Survivors Study; CRS, cardiotoxicity risk score; D, development; ECG, electrocardiogram; EKZ/AMC, Emma Children's Hospital/Academic Medical Center; EV, external validation; FHCRC, Fred Hutchinson Cancer Research Center; GLS, global longitudinal strain; HCT, hematopoietic stem cell transplantation; HCT-CI, Hematopoietic Cell Transplantation-specific Comorbidity Index; HF, heart failure; HFA-ICOS, Heart Failure Association - International Cardio-Oncology Society; HFrEF, heart failure with reduced ejection fraction; IMiDs, immunomodulatory drugs; LVEF, left ventricular ejection fraction; NHL, non-Hodgkin lymphoma; NI, no information; NWTS, National Wilms Tumor Study; PCP-HF, Pooled Cohort equations to Prevent HF; QRISK3, Cardiovascular Disease Risk Score; SCORE-(OP), Systematic COronary Risk Evaluation – (Older Persons); SEER, Surveillance, Epidemiology, and End Results; SJLIFE, St. Jude Lifetime Cohort Study; UK, United Kingdom; USA, United States of America; yr(s), year(s).

*Age at cancer diagnosis, in years. Results will be presented according to the information available in the primary studies and rounded to integer values: interval containing the median age, median age (interquartile range), or mean age ± standard deviation. [±]For further details on outcome definition, please see **Supplemental Table 7**. [§]EPV values were calculated according to the number of coefficients (categories/levels of predictors) and not only the number of predictors. [¶]Other childhood cancers may include: CNS tumors, Wilms tumor, neuroblastoma, bone tumor, rhabdomyosarcoma, soft tissue sarcoma. [†]Patients treated with chemotherapy, with anthracycline used in a minority of cases. Reported agents: antimetabolites, fluoropyrimidines, alkylating agents, and anti-microtubule agents. [‡]Other machine learning modelling methods were used but with inferior results.

Supplemental Table 11. Models that were identified from external validation studies, but of which the original development study did not fulfill the inclusion criteria of this systematic review

Model	Original paper	Target population	Target outcome	Validation studies included in this review
**AI-ECG**	Sangha et al, 2023 [103]	Hospital ECG records	Left ventricular systolic dysfunction (diagnosis)	Oikonomou EK, 2024 [47]
**ECG score**	Chatterjee et al, 2020 [104]	Patients with coronary artery disease	Sudden and/or arrhythmic death	Pohl J, 2021 [50]
**Framingham risk score** (Blood lipids)	Wilson et al, 1998 [105]	General population, 30-74 yrs	Coronary heart disease	McCracken C, 2024 [16]; Law Q, 2017 [14].
**Framingham risk score** (BMI)	D'Agostino et al, 2008 [106]	General population, 30-74 yrs	Cardiovascular disease	McCracken C, 2024 [16]
**HCT-CI**	Sorror et al, 2005 [107]	Patients proposed for HCT	Non-relapse mortality and overall survival	Vasbinder A, 2024 [23]
**PCP-HF**	Khan et al, 2019 [108]	General population, 30-79 yrs	Heart failure	McCracken C, 2024 [16]
**QRISK3**	Hippisley-Cox et al, 2017 [109]	General population, 25-84 yrs	Cardiovascular disease	McCracken C, 2024 [16]; Abiodun A, 2024 [4].
**SCORE**	Conroy et al, 2003 [110]	General population	Fatal cardiovascular disease	Caro-Codón J, 2022 [25]
**SCORE2 / SCORE-OP**	Hageman et al, 2021 [111]<br>De Vries et al, 2021 [112]	General population, 40-69 yrs<br>General population, >70 yrs	Cardiovascular disease	McCracken C, 2024 [16]
Developed from consensus / expert opinion				
**ASCO risk score**	Armenian et al, 2017 [113]	Cancer patients	-	Jacobs J, 2022 [39]
**HFA-ICOS risk tool**	Lyon et al, 2020 [114]	Cancer patients	-	Liu X, 2022 [44]; Battisti NML, 2021 [8]; Cronin M, 2023 [10]; Tini G, 2022 [22]; Shibata T, 2023 [20]; Suntheralingam S, 2022 [54]; Di Lisi D, 2024 [31]; Rivero-Santana B, 2024 [51]; Fernando F, 2024 [12]; Stefanini B, 2024 [21].
**Cardiotoxicity risk score (CRS)**	Herrmann et al, 2014 [115]	Cancer patients	-	Jacobs J, 2022 [39]

AI-ECG, Artificial Intelligence-enhanced electrocardiography; ASCO, American Society of Clinical Oncology; BMI, body mass index; ECG, electrocardiogram; HCT-CI, Hematopoietic Cell Transplantation-specific Comorbidity Index; HFA-ICOS, Heart Failure Association - International Cardio-Oncology Society; PCP-HF, Pooled Cohort equations to Prevent HF; QRISK3, Cardiovascular Disease Risk Score; SCORE-(OP), Systematic COronary Risk Evaluation – (Older Persons).

Supplemental Table 12. Overview of predictor groups included in each model.

Model	Predictors (n.)	Age, sex	CVRF	Prior comorbidities	ECG	Imaging parameters	Lab.	Cancer type/stage	Cancer treatment	Genetic markers	Other	IV c-stat*	EV c-stat
CHILDREN, ADOLESCENTS AND YOUNG ADULTS (AYA)													
**Childhood Cancers**													
Chaix 2020 [clinical]	7	X							X		X	< 0.6	-
Chaix 2020 [genetic]	31									X		> 0.7	-
Chaix 2020 [both]	38	X							X	X	X	> 0.7	-
Chen 2020 [age 20]	7	X	X						X			> 0.7	-
Chen 2020 [age 25]	7	X	X						X			> 0.7	-
Chen 2020 [age 30]	7	X	X						X			> 0.7	-
Chen 2020 [age 35]	7	X	X						X			0.6-0.7	-
Chow 2015 [simple]	4	X							X			> 0.7	> 0.7$^{\pm}$
Chow 2015 [standard]	4	X							X			> 0.7	> 0.7$^{\pm}$
Chow 2015 [heart dose]	4	X							X			> 0.7	> 0.7
Gunturkun 2021 [clinical]	7		X						X		X	0.6-0.7	-
Gunturkun 2021 [ECG]	86				X							> 0.7	-
Gunturkun 2021 [both]	93		X		X				X		X	> 0.7	-
Leerink 2021	3					X			X			> 0.7	> 0.7
Liu Z 2022	4					X	X		X			> 0.7	-
**Hodgkin lymphoma (AYA)**													
De Vries 2022 [simple]	4	X							X			0.6-0.7	-
De Vries 2022 [dose]	4	X							X			0.6-0.7	-
ADULTS													
**Breast Cancer**													
Abdel-Qadir 2019	10	X	X	X								> 0.7	0.6-0.7
AI-ECG (Sangha, 2023)	Unclear				X							-	0.6-0.7
Chang 2022	15	X	X	X		X		X	X			> 0.7	-
Dranitsaris 2008	7	X	X	X					X			> 0.7	-
Ezaz 2014	7	X	X	X					X			-	< 0.6
Fogarassy 2019	13	X	X	X				X	X			> 0.7	-
Framingham 2008	7	X	X				X					-	-
Gomez-Vecino 2023	5									X		0.6-0.7	-
HFA-ICOS [anti-Her2]	18	X	X	X		X	X		X			-	0.6-0.7§
HFA-ICOS [anthracyclines]	16	X	X	X		X	X		X			-	> 0.7
Jones 2020 [1]	6					X						-	-
Jones 2020 [2]	3					X						-	-
Jones 2020 [RF]	6					X						> 0.7	-
Kim 2021 [CHEMO-RADIAT]	11	X	X	X					X			0.7-0.8	
Kotwinski 2016	4		X						X			> 0.7	

Liu B 2022	3							X	X	X		-	
Liu X 2022 [ABSDELL]	7	X	X			X						> 0.7	
Nguyen 2024	Unclear	X		X				X				-	> 0.7
Otchere 2023	8	X	X	X					X			0.6-0-7	
Romond 2012	2	X				X						0.6-0-7	0.6-0.7
Sun 2021	4	X	X					X	X			-	
Upshaw 2019	3	X	X			X						0.6-0.7	
Yu 2024	9	X	X	X		X			X		X	0.6-0.7	
**Hematopoietic Stem Cell Transplantation**													
Armenian 2018 [full model]	6	X	X						X			-	0.6-0.7¶
Vasbinder 2024 [CARE-BMT]	11	X		X			X		X		X	> 0.7	> 0.7
HCT-CI												-	0.6-0.7
**Multiple Myeloma**													
Astarita 2021 [CFZ CVAE]	5		X			X						> 0.7	-
Yuan 2023	3	X	X			X						0.6-0.7	
**Other Hematological Malignancies**													
Diamond 2022	4	X	X				X	X				-	-
HFA-ICOS [BCR-ABL]	19	X	X	X	X	X	X		X			-	0.6-0.7
Kang 2019	6	X		X		X		X	X				0.6-0.7
Yagi 2024 [model 1]	13	X	X	X	X	X		X	X		X	-	-
**Hepatocellular Carcinoma**													
Carballo-Folgoso 2021 [CARDIOSOR]	5	X	X	X								-	< 0.6
HFA-ICOS [VEGF inhibitors]	18	X	X	X	X	X	X		X			-	0.6-0.7
**Lung Cancer**													
Heilbroner 2021 [full]	356	X	X				X		X			0.6-0.7	-
Heilbroner 2021 [simplified]	20	X	X				X		X			0.6-0.7	-
**Colorectal Cancer**													
Li 2022 [XGBoost]	Unclear	X	X	X							X	> 0.7	
**Multiple Cancers**													
ASCO risk score	Unclear	X	X	X		X			X			-	-
CRS (Herrmann, 2014)	Unclear	X	X	X					X			-	< 0.6
ECG score (Chatterjee, 2020)	4				X							-	> 0.7
Framingham 2008	7	X	X				X					-	0.6-0.7
Framingham 2008 (BMI)	6	X	X									-	0.6-0.7
Jacobs 2022 [1]	6		X									< 0.6	-
Jacobs 2022 [2]	7		X						X			0.6-0.7	-
Ozturk 2021	7	X	X	X		X	X					> 0.7	
PCP-HF	12	X	X		X		X				X	-	> 0.7
QRISK3	21	X	X	X			X				X	-	0.6-0.7
SCORE	5	X	X				X					-	-

SCORE2/SCORE-OP	6	X	X				X					-	0.6-0.7

AI-ECG, Artificial Intelligence-enhanced Electrocardiography; ASCO, American Society of Clinical Oncology; BMI, body mass index; CARE-BMT, Cardiovascular Registry in Bone Marrow Transplantation; CRS, cardiotoxicity risk score; CVRF, cardiovascular risk factors; ECG, electrocardiogram; EV, external validation; HCT-CI, Hematopoietic Cell Transplantation-specific Comorbidity Index; HFA-ICOS, Heart Failure Association – International Cardio-Oncology Society; IV, internal validation; Lab., laboratory parameters; PCP-HF, Pooled Cohort equations to Prevent HF; QRISK3, Cardiovascular Disease Risk Score; SCORE-(OP), Systematic Coronary Risk Evaluation – (Older Persons).

*Models who did not undergo internal validation were not reported here, even when reporting apparent performance measures. ±These models were validated three times, with C-statistic ≥0.8 in two of the external validations. §C-statistic from four external validation studies, two <0.6 and two between 0.6-0.7. ¶Two external validations: C-statistic 1 = 0.6, C-statistic 2 = 0.72.

Supplemental Table 13. Performance and characteristics of included models in development and external validation settings.

Model	Study		Outcome		Competing risks accounted for	Internal validation method	Type of external validation	Prediction Horizon	Discrimination		Calibration	
	Aim	Author, year (cohort)	Outcome	Type					C-stat*	95% CI	Plot±	Other measure
CHILDREN, ADOLESCENTS AND YOUNG ADULTS												
**Childhood Cancers**												
Chaix 2020 [clinical]	D	Chaix MA, 2020	LVEF decline	Binary	No	Bootstrap		-	0.59	0.51-0.67	-	-
Chaix 2020 [genetic]	D	Chaix MA, 2020	LVEF decline	Binary	No	Bootstrap		-	0.71	0.63-0.80	-	-
Chaix 2020 [both]	D	Chaix MA, 2020	LVEF decline	Binary	No	Bootstrap		-	0.72	0.63-0.80	-	-
Chen 2020 [age 20]	D	Chen Y, 2020	HF	TTE	Yes	Split-sample		50 yrs (age)	0.78	-	-	-
Chen 2020 [age 25]	D	Chen Y, 2020	HF	TTE	Yes	Split-sample		50 yrs (age)	0.71	-	-	-
Chen 2020 [age 30]	D	Chen Y, 2020	HF	TTE	Yes	Split-sample		50 yrs (age)	0.75	-	-	-
Chen 2020 [age 35]	D	Chen Y, 2020	HF	TTE	Yes	Split-sample		50 yrs (age)	0.69	-	-	-
Chow 2015 [simple]	D	Chow E, 2015 (*CCSS*)	HF	TTE	No	Cross-validation		40 yrs (age)	0.72	-	-	-
	EV	Chow E, 2015 (*EKZ/AMC*)	HF	TTE	Yes		Geographic	40 yrs (age)	0.75	-	-	-
	EV	Chow E, 2015 (*NWTS*)	HF	TTE	Yes		Domain	40 yrs (age)	0.79	-	-	-
	EV	Chow E, 2015 (*SJLIFE*)	HF	TTE	Yes		Geographic	40 yrs (age)	0.63	-	-	-
Chow 2015 [standard]	D	Chow E, 2015 (*CCSS*)	HF	TTE	No	Cross-validation		40 yrs (age)	0.76	-	-	-
	EV	Chow E, 2015 (*EKZ/AMC*)	HF	TTE	Yes		Geographic	40 yrs (age)	0.80	-	-	-
	EV	Chow E, 2015 (*NWTS*)	HF	TTE	Yes		Domain	40 yrs (age)	0.82	-	-	-
	EV	Chow E, 2015 (*SJLIFE*)	HF	TTE	Yes		Geographic	40 yrs (age)	0.68	-	-	-

Chow 2015 [heart dose]	D	Chow E, 2015 (*CCSS*)	HF	TTE	No	Cross-validation		40 yrs (age)	0.77	-	-	-
	EV	Chow E, 2015 (*EKZ/AMC)*	HF	TTE	Yes		Geographic	40 yrs (age)	0.78	-	-	-
Gunturkun 2021 [clinical]	D	Gunturkun F, 2021	LVEF decline	Binary	No	Cross-validation		-	0.69	0.64-0.74	-	-
Gunturkun 2021 [ECG]	D	Gunturkun F, 2021	LVEF decline	Binary	No	Cross-validation		-	0.87	0.83-0-90	-	-
Gunturkun 2021 [both]	D	Gunturkun F, 2021	LVEF decline	Binary	No	Cross-validation		-	0.89	0.86-0.91	-	-
Leerink 2021	D	Leerink JM, 2021	LVEF decline	TTE	Not fully§	Bootstrap		10 years	0.87	0.71-0.98	“good”	-
	EV	Leerink JM, 2021	LVEF decline	TTE	Not fully§		Geographic	10 years	0.86	0.83-0.89	“good”	
Liu Z. 2022	D	Liu Z., 2022	HF/ LVEF decline	Binary	No	Bootstrap		1 year	0.773	-	“good”	HL test p=0.92
**Hodgkin Lymphoma**												
De Vries 2022 [simple]	D	De Vries S, 2022	HF	TTE	Yes	Bootstrap + cross-validation		20 years	0.71	0.64-0.79	-	O/E 0.81
								30 years	0.69	0.64-0.75	-	O/E 0.87
De Vries 2022 [dose]	D	De Vries S, 2022	HF	TTE	Yes	Bootstrap + cross-validation		20 years	0.70	0.64-0.72	-	O/E 0.85
								30 years	0.68	0.62-0.74	-	O/E 0.92
ADULTS												
**Breast Cancer**												
Abdel-Qadir 2019	D	Abdel-Qadir H, 2019	Composite	TTE	Yes	Split-sample		5 years	0.819	0.81-0.83	“good”	-
								10 years	0.798	0.79-0-81	“good”	-
	EV	Mery B, 2022	Composite	TTE	No		Geographic	5 years	0.62	‘	“poor”	-
								10 years	0.66	‘	“poor”	-
AI-ECG	EV	Oikonomou EK, 2024	HF / LVEF decline <50%	TTE	Yes		Domain	24 months	0.64	0.59-0.70	-	-

			LVEF <40%						0.82	0.74-0.89	-	-
Chang 2022 [CTRCD]	D	Chang WT, 2022	LVEF decline	Binary	No	Split-sample		3 years	0.664	-	-	-
Change 2022 [HFrEF]	D	Chang WT, 2022	HF	Binary	No	Split-sample		3 years	0.79	-	-	-
Dranitsaris 2008	D	Dranitsaris G, 2008	HF / LVEF decline	Binary	No	Bootstrap		-	0.84	0.79-0.89	-	-
Ezaz 2014	D	Ezaz G, 2014	HF	TTE	No	Split-sample		3 years	-	-	-	HL test p=0.76
	EV	Moey MYY, 2019	LVEF decline	Binary	No		Geographic	-	-	-	-	-
	EV	Rushton M, 2017	HF / LVEF decline	Binary	No		Geographic	3 years	-	-	-	-
	EV	Suntheralingam S, 2022	HF / LVEF decline	Binary	No		Geographic	-	0.51	0.48-0.55	"poor"	-
Fogarassy 2019	D	Fogarassy G, 2019	HF	Binary	No	Split-sample		-	0.79	-	-	HL test p=0.78
Framingham 2008 (blood lipids)	EV	Law Q, 2017	Composite	TTE	No		Domain	10 years	-	-	-	-
Gomez-Vecino 2023	D	Gomez-Vecino A, 2023	HF / LVEF decline	Binary	No	Bootstrap		-	0.681	-	-	-
HFA-ICOS [anti-Her2]	EV	Liu X, 2022	LVEF decline	Binary	No		N/A	3 years	0.67	-	-	-
	EV	Battisti NML, 2021	Composite	Binary	No		N/A	-	0.56	-	-	-
	EV	Cronin M, 2023	Composite	Binary	No		N/A	-	0.64	0.51-0.76	-	-
	EV	Tini G, 2022	Composite	Binary	No		N/A	-	-	-	-	-
	EV	Suntheralingam S, 2022	HF / LVEF decline	Binary	No		N/A	-	0.58	0.54-0.62	"poor"	-
HFA-ICOS [anthracyclines]	EV	Tini G, 2022	Composite	Binary	No		N/A	-	-	-	-	-
	EV	Di Lisi D, 2024	Composite	Binary	No		N/A	-	-	-	-	-
	EV	Rivero-Santana B, 2024	HF / LVEF decline	TTE	No		N/A	12 months	0.78	0.70-0.82	"good"	Brier 0.04
Jones 2020 [1]	D	Jones KA, 2020	LVEF decline	Binary	No	-		-	0.81	-	-	-
Jones 2020 [2]	D	Jones KA, 2020	LVEF decline	Binary	No	-		-	0.88	-	-	-

Jones 2020 [RF]	D	Jones KA, 2020	LVEF decline	Binary	No	Cross-validation		-	0.87	-	-	-
Kim 2021 [CHEMO-RADIAT]	D	Kim DY, 2021	Composite	TTE	No	Split-sample		1 year	0.811	0.701-0.915	-	HL test p=0.18
								3 years	0.876	0.786-0.966	-	-
								7 years	0.842	0.725-0.959	-	-
Kotwinski 2016	D	Kotwinski P, 2016	HF / LVEF decline	Binary	No	Bootstrap		-	0.78	0.70-0.86	-	-
Liu B 2022	D	Liu B, 2022	Composite	Binary	No	-		-	0.604	0.548-0.660	“good”	-
Liu X 2022 [ABSDELL]	D	Liu X, 2022	LVEF decline	TTE	No	Bootstrap		1 year	0.801	-	“good”	Brier 0.118
								3 years	0.881	-	“good”	Brier 0.091
Nguyen 2024	D	Nguyen QT, 2024	Composite	Binary	No	Split-sample		1 year	-	-	-	-
	EV	Nguyen QT, 2024	Composite	Binary	No		Geographic	1 year	0.897	-	-	-
Otchere 2023	D	Otchere P, 2023	LVEF decline	Binary	No	Split-sample		1 year	0.639	-	-	-
Romond 2012	D	Romond EH, 2012	Composite	TTE	Yes	Bootstrap		5 years	0.70	-	plot[¶]	-
	EV	Advani P, 2016	Composite	TTE	No		Geographic	-	0.665	-	-	-
	EV	Suntheralingam S, 2022	HF / LVEF decline	Binary	No		Geographic	-	0.60	0.55-0.65	“poor”	-
SCORE	EV	Caro-Codón J, 2022	HF / LVEF decline	TTE	No		Domain	2 years	-	-	-	-
Sun 2021	D	Sun Y, 2021	HF / LVEF decline	Binary	No	-		-	0.781	0.693-0.869	“good”	HL test p=2,70
Upshaw 2019	D	Upshaw JN, 2019	HF / LVEF decline	Binary	No	Bootstrap		1 year	0.68	0.62-0.75	-	-
Yu 2024	D	Yu AF, 2024	LVEF decline	TTE	No	Bootstrap		1 year	0.68	-	“good”	-
**Hematopoietic Stem Cell Transplantation**												
Armenian 2018 [full model]	D	Armenian S, 2018 (COH)	Composite (HF+CAD)	TTE	Yes	-	-	10 years	0.72	-	-	-
			HF						0.70	-	-	-

	EV	Armenian S, 2018 (FHCRC)	Composite (HF+CAD)	TTE	Yes	-	Geographic	10 years	0.72	-	-	-
			HF						0.66	-	-	-
	EV	Vasbinder A, 2024	Composite (HF+AMI)	TTE	Yes	-	Geographic	10 years	0.61	0.55-0.69	-	-
HCT-CI	EV	Vasbinder A, 2024	Composite (HF+AMI)	TTE	Yes	-	Domain	10 years	0.62	0.56-0.68	-	-
Vasbinder 2024 [CARE-BMT]	D	Vasbinder A, 2024	Composite†	TTE	Yes	Cross-validation	-	100 days	0.71	0.66-0.76	-	-
								1 year	0.71	0.66-0.77	-	-
								5 years	0.74	0.70-0.78	-	-
								10 years	0.77	0.72-0.83	-	-
	EV	Vasbinder A, 2024	Composite	TTE	Yes	-	Geographic	100 days	0.73	0.65-0.81	-	-
								1 year	0.73	0.66-0.80	-	-
								5 years	0.76	0.68-0.82	-	-
								10 years	0.80	0.71-0.89	-	-
			Composite (HF+AMI)	TTE				10 years	0.77	0.72-0.83	-	-
**Multiple Myeloma**												
Astarita 2021 [CFZ CVAE score]	D	Astarita A, 2021	Composite	Binary	No	Bootstrap	-	-	0.76	-	"perfect"	-
Yuan 2023	D	Yuan S, 2023	Composite	TTE	No	Bootstrap	-	-	0.66	0.51-0.81	"good"	-
**Other Hematological Malignancies**												
Diamond 2022	D	Diamond A, 2022	Composite	TTE	Yes	-		-	-	-	-	-
HFA-ICOS [BCR-ABL]	EV	Fernando F, 2024	Composite	TTE	No		N/A	-	0.65	-	-	-
HFA-ICOS [unspecified]‡	EV	Shibata T, 2023	Composite	TTE	No		N/A	-	-	-	-	-
Kang 2019	D	Kang Y, 2019	HF	TTE	Yes	Cross-validation		-	**		-	-

	EV	Doukas PG, 2022	HF	TTE	Not fully[§]		Geographic	1 year	0.64	0.55-0.74	-	-
Yagi 2024 [model 1]	D	Yagi R, 2024	LVEF decline	TTE	Yes	-		0.5 year	0.780	0.724-0.836	-	-
								1 year	0.793	0.738-0.848	-	-
								1.5 years	0.781	0.726-0.837	-	-
								2 years	0.781	0.722-0.840	-	-
**Hepatocellular Carcinoma**												
Carballo-Folgoso 2021 [CARDIOSOR scale]	D	Carballo-Folgoso L, 2021	Composite	TTE	Yes	-		-	0.663	-	-	-
	EV	Stefanini B, 2024	Composite	TTE	Yes		Geographic	-	0.562	0.501-0.634	-	-
HFA-ICOS [VEGF inhibitors]	EV	Stefanini B, 2024	Composite	TTE	Yes		N/A	-	0.671	0.583-0.758	-	-
**Lung Cancer**												
Heilbroner 2021 [full]	D	Heilbroner SP, 2021	Composite	TTE	No	Split-sample		-	0.66	0.57-0.71	-	-
Heilbroner 2021 [simplified]	D	Heilbroner SP, 2021	Composite	TTE	No	Split-sample		-	0.65	-	-	-
**Colorectal Cancer**												
Li 2022 [XGBoost]	D	Li C, 2022	Composite	Binary	No	Cross-validation		30 days	0.816	0.806-0.826	-	-
**Multiple Cancers[±±]**												
ASCO risk score	EV	Jacobs J, 2022	LVEF decline	Binary	No		N/A	-	-	-	-	-
CRS (Herrmann, 2014)	EV	Jacobs J, 2022	LVEF decline	Binary	No		N/A	-	0.593	0.550-0.676	-	-
CRS (Herrmann, 2014) – updated[§§]	Up	Jacobs J, 2022	LVEF decline	Binary	No		N/A	-	0.654	0.601-0.715	-	-
ECG score (Chatterjee, 2020)	EV	Pohl J, 2021	LVEF / GLS decline	Binary	No		Domain	-	0.84	0.77-0.92	-	-
Framingham 2008 (blood lipids)	EV	McCracken C, 2024	Composite	TTE	Yes		Domain	10 years	0.67	0.66-0.68	plot[¶]	-
Framingham 2008 (BMI)	EV	McCracken C, 2024	Composite	TTE	Yes		Domain	10 years	0.68	0.67-0.69	plot[¶]	-

Jacobs 2022 [1]	D	Jacobs J, 2022	LVEF decline	Binary	No	Bootstrap		-	0.580	0.525-0.642	-	-
Jacobs 2022 [2]	D	Jacobs J, 2022	LVEF decline	Binary	No	Bootstrap		-	0.613	0.550-0.676	-	-
Ozturk 2021	D	Ozturk C, 2021	LVEF / GLS decline	Binary	No	Split-sample		-	0.983	0.854-1.000	-	-
PCP-HF	EV	McCracken C, 2024	HF	TTE	Yes		Domain	10 years	0.71	0.69-0.72	plot¶	-
QRISK3	EV	McCracken C, 2024	Composite	TTE	Yes		Domain	10 years	0.68	0.67-0.69	plot¶	-
	EV	Abiodun A, 2024	Composite	TTE	No		Domain	3m after last treatment	-	-	-	-
SCORE2/SCORE-OP	EV	McCracken C, 2024	Composite	TTE	Yes		Domain	10 years	0.67	0.67-0.68	"best"	-

(-) values equal to "not reported". AI-ECG, Artificial Intelligence-enhanced Electrocardiography; ASCO, American Society of Clinical Oncology; BMI, body mass index; CARE-BMT, Cardiovascular Registry in Bone Marrow Transplantation; CCSS, Childhood Cancer Survivors Study; CRS, cardiotoxicity risk score; C-stat, C-statistic; D, development; ECG, electrocardiogram; EKZ/AMC, Emma Children's Hospital/Academic Medical Center; EV, external validation; FHCRC, Fred Hutchinson Cancer Research Center; GLS, global longitudinal strain; HCT-CI, Hematopoietic Cell Transplantation-specific Comorbidity Index; HF, heart failure; HFA-ICOS, Heart Failure Association – International Cardio-Oncology Society; HL, Hosmer-Lemeshow test; LVEF, left ventricular ejection fraction; N/A, not applicable; NWTS, National Wilms Tumor Study; O/E, observed-expected ratio; PCP-HF, Pooled Cohort equations to Prevent HF; QRISK3, Cardiovascular Disease Risk Score; SCORE-(OP), Systematic Coronary Risk Evaluation – (Older Persons); SJLIFE, St. Jude Lifetime Cohort Study; TTE, time-to-event; Up, model updated.

*When both C-statistic and the area under the receiver operating curve (AUC) were reported, C-statistic was extracted preferably. ±Calibration as assessed by the authors of the study. §Competing risks taken into account only for cumulative incidence estimates but not to calculate performance measures. ¶Calibration plot presented but calibration not judged by the authors. †The authors present performance measures when predicting: 1) all cardiovascular events and 2) severe cardiovascular events. We report severe cardiovascular events, as this definition still includes heart failure and excludes less relevant outcomes. ‡HFA-ICOS tool validated in patients with leukemia, malignant lymphomas, multiple myeloma, and breast cancer. It is unclear which of the HFA-ICOS models were used. **The authors present a time-dependent receiver operating curve, but do not report a C-statistic for any time-point. ±±Most of the models included in this category were developed or validated in studies where different cancers were combined and none comprised the majority of the study population. §§The authors updated the CRS (Herrmann, 2014) recalculating the coefficients of the tool using their own population. Since they did not assess the performance of the recalibrated model on an independent external population, neither used internal validation techniques, the reported performance measure is a measure of apparent performance.

Supplemental Table 14. Risk of bias and applicability assessment using the PROBAST tool for each model development and external validation

Study	Model	Aim	*RoB* Participants	*RoB* Predictors	*RoB* Outcome	*RoB* Analysis	Overall RoB	*Applicability* Participants	*Applicability* Predictors	*Applicability* Outcomes	Overall Applicability
Abdel-Qadir H, 2019	Abdel-Qadir 2019	D	High	High	High	Low	High	Low	Low	High	High
Abiodun A, 2024	QRISK3	EV	High	High	Low	High	High	Low	Low	High	High
Advani P, 2016	Romond 2012	EV	Low	High	High	High	High	High	Low	Low	High
Armenian S, 2018	Armenian 2018 [full model]	D	High	Low	Unclear	High	High	Low	Low	Low	Low
		EV	High	Low	Unclear	High	High	High	Low	Low	High
Astarita A, 2021	CFZ CVAE score	D	High	Low	High	High	High	Low	High	High	High
Battisti, 2021	HFA-ICOS [anti-Her2]	EV	Low	High	High	High	High	Low	Low	Low	Low
Carballo-Folgoso L, 2021	CARDIOSOR SCALE	D	High	High	High	High	High	Low	Low	High	High
Caro-Codón J, 2022	SCORE	EV	High	Low	Unclear	High	High	Low	Low	Low	Low
Chaix MA, 2020	Chaix 2020 [clinical]	D	High	High	High	High	High	Low	Low	Low	Low
	Chaix 2020 [genetic]	D	High	High	High	High	High	Low	Low	Low	Low
	Chaix 2020 [both]	D	High	High	High	High	High	Low	Low	Low	Low
Chang WT, 2022	Chang 2022 **[CTRCD]**	D	Low	Low	Low	High	High	Low	Low	Low	Low
	Chang 2022 **[HFrEF]**	D	Low	Low	Low	High	High	Low	Low	Low	Low
Chen Y, 2020	Chen 2020 [age 20]	D	Low	High	High	High	High	Low	Low	Low	Low
	Chen 2020 [age 25]	D	Low	High	High	High	High	Low	Low	Low	Low
	Chen 2020 [age 30]	D	Low	High	High	High	High	Low	Low	Low	Low
	Chen 2020 [age 35]	D	Low	High	High	High	High	Low	Low	Low	Low

Chow E, 2015	Chow 2015 [simple]	D	Low	High	High	High	High	Low	Low	Low	Low
		EV (EKZ/AMC)	Low	High	Low	High	High	Low	Low	Low	Low
		EV (NWTS)	High	Low	High	High	High	Low	Low	High	High
		EV (SJLIFE)	Low	Low	High	High	High	Low	Low	Low	Low
	Chow 2015 [standard]	D	Low	High	High	High	High	Low	Low	Low	Low
		EV (EKZ/AMC)	Low	High	Low	High	High	Low	Low	Low	Low
		EV (NWTS)	High	Low	High	High	High	Low	Low	High	High
		EV (SJLIFE)	Low	Low	High	High	High	Low	Low	Low	Low
	Chow 2015 [heart dose]	D	Low	High	High	High	High	Low	Low	Low	Low
		EV (EKZ/AMC)	Low	High	Low	High	High	Low	Low	Low	Low
Cronin M, 2023	HFA-ICOS [anti-Her2]	EV	Low	High	Unclear	High	High	Low	Low	Low	Low
De Vries S, 2022	De Vries 2022 [simple]	D	Low	Low	High	High	High	Low	Low	Low	Low
	De Vries 2022 [dose]	D	Low	Low	High	High	High	Low	Low	Low	Low
Di Lisi D, 2024	HFA-ICOS [anthracyclines]	EV	Low	High	High	High	High	Low	Low	Unclear	Unclear
Diamond A, 2021	Diamond 2021	D	High	High	High	High	High	Low	Low	Low	Low
Doukas PG, 2022	Kang 2019	EV	High	Low	Low	High	High	Low	Low	Low	Low
Dranitsaris G, 2008	Dranitsaris 2008	D	High	Low	Low	High	High	High	High	Low	High
Ezaz G, 2014	Ezaz 2014	D	High	High	High	High	High	Low	Low	Low	Low
Fernando F, 2024	HFA-ICOS [BCR-ABL]	EV	High	High	High	High	High	Low	Low	High	High
Fogarassy G, 2019	Fogarassy 2019	D	Low	High	High	High	High	Low	Low	Low	Low

Gomez-Vecino A, 2023	Gomez-Vecino 2023	D	Unclear	Low	High	High	High	Low	Low	Low	Low
Gunturkun F, 2021	Gunturkun 2021 [clinical]	D	Low	Low	Unclear	High	High	Low	Low	Low	Low
	Gunturkun 2021 [ECG]	D	Low	Low	Unclear	High	High	Low	Low	Low	Low
	Gunturkun 2021 [both]	D	Low	Low	Unclear	High	High	Low	Low	Low	Low
Heilbroner SP, 2021	Heilbroner 2021 [full]	D	Unclear	Low	High	High	High	Low	Unclear	High	High
	Heilbroner 2021 [simplified]	D	Unclear	Low	High	High	High	Low	Low	High	High
Jacobs J, 2022	Jacobs 2022 [1]	D	High	Unclear	Unclear	High	High	High	Low	Low	High
	Jacobs 2022 [1]	D	High	Unclear	Unclear	High	High	High	Low	Low	High
	ASCO risk score	EV	High	Unclear	Unclear	High	High	High	Low	Low	High
	CRS (Herrmann, 2014)	EV	High	Unclear	Unclear	High	High	High	Low	Low	High
Jones KA, 2020	Jones 2020 [1]	D	Low	Low	Low	High	High	Low	Low	Low	Low
	Jones 2020 [2]	D	Low	Low	Low	High	High	Low	Low	Low	Low
	Jones 2020 [RF]	D	Low	Low	Low	High	High	Low	Low	Low	Low
Kang Y, 2019	Kang 2019	D	High	High	Low	High	High	Low	Low	Low	Low
Kim DY, 2021	CHEMO-RADIAT	D	High	High	High	High	High	Low	Low	Low	Low
Kotwinski P, 2016	Kotwinski 2016	D	High	Low	Low	High	High	High	Low	Low	High
Law Q, 2017	Framingham	EV	High	Low	High	High	High	High	Low	High	High
Leerink JM, 2021	Leerink 2021	D	Low	Low	Low	High	High	Low	Low	Low	Low
		EV	Low	Low	Low	Low	Low	Low	Low	Low	Low
Li C, 2022	Li 2022 [XGBoost]	D	Low	Unclear	High	High	High	Low	Unclear	Unclear	Unclear
Liu B, 2022	Liu B 2022	D	Low	Low	Low	High	High	High	Low	High	High
Liu X, 2022	ABSDELL	D	High	Unclear	High	High	High	High	Low	Low	High
	HFA-ICOS	EV	High	Unclear	High	High	High	High	Low	Low	High
Liu Z, 2022	Liu Z 2022	D	Low	Low	Unclear	High	High	Low	Low	Low	Low
Mccracken C, 2024	QRISK3	EV	Low	Low	High	Low	High	Low	Low	High	High
	Framingham (BMI)	EV	Low	Low	High	Low	High	Low	Low	High	High

	Framingham (blood lipids)	EV	Low	Low	High	Low	High	Low	Low	High	High
	SCORE2 / SCORE-OP	EV	Low	Low	High	Low	High	Low	Low	High	High
	PCP-HF	EV	Low	Low	High	Low	High	Low	Low	Low	Low
Mery B, 2022	Abdel-Qadir 2019	EV	High	High	High	Unclear	High	Low	Low	High	High
Moey MYY, 2019	Ezaz 2014	EV	Low	High	High	High	High	Low	Low	Low	Low
Nguyen QT, 2024	Nguyen 2024	D	High	Low	High	High	High	Low	Low	Unclear	Unclear
		EV	High	Low	High	High	High	Low	Low	Unclear	Unclear
Oikonomou E. K., 2024	AI-ECG (Sangha, 2023)	EV	High	Low	High	High	High	High	Low	Low	High
Otchere P, 2023	Otchere 2023	D	High	Low	High	High	High	Low	Low	Low	Low
Ozturk C, 2021	Ozturk 2021	D	Low	Low	Low	High	High	Low	Low	Low	Low
Pohl J, 2021	ECG score (Chatterjee, 2020)	EV	High	Low	High	High	High	High	Low	Low	High
Rivero-Santana B, 2024	HFA-ICOS [anthracyclines]	EV	Low	Low	Unclear	High	High	Low	Low	Low	Low
Romond EH, 2012	Romond 2012	D	Low	Low	Unclear	High	High	High	Low	Low	High
Rushton M, 2017	Ezaz 2014	EV	High	Low	High	High	High	High	Low	Low	High
Shibata T, 2023	HFA-ICOS [unspecified]	EV	High	Low	Unclear	High	High	High	Low	High	High
Stefanini B, 2024	Carballo-Folgoso 2021 [CARDIOSOR]	EV	High	Low	High	High	High	Low	Low	High	High
	HFA-ICOS [VEGF inhibitors]	EV	High	High	High	High	High	Low	Low	High	High
Sun Y, 2021	Sun 2021	D	High	Low	Unclear	High	High	High	Low	Low	High
Suntheralingam S, 2022	HFA-ICOS [anti-Her2]	EV	High	High	High	Unclear	High	Low	Low	Low	Low
	Romond 2012	EV	High	High	High	Unclear	High	Low	Low	Low	Low
	Ezaz 2014	EV	High	High	High	Unclear	High	Low	Low	Low	Low
Tini G, 2021	HFA-ICOS [anthracyclines]	EV	High	High	High	High	High	High	Low	High	High

	HFA-ICOS [anti-Her2]	EV	High	High	High	High	High	High	Low	Low	High
Upshaw JN, 2019	Upshaw 2019	D	Low	Low	High	High	High	High	Low	Low	High
Vasbinder A, 2024	CARE-BMT	D	Low	Unclear	High	High	High	Low	Low	High	High
		EV	Low	Unclear	High	High	High	Low	Low	High	High
	Armenian 2018 [full model]	EV	Low	Unclear	Unclear	High	High	Low	Low	High	High
	HCT-CI	EV	Low	Unclear	High	High	High	Low	Low	High	High
Yagi R, 2024	Yagi 2024 [Model 1]	D	High	High	High	High	High	High	Low	Low	High
Yu AF, 2023	Yu 2024	D	Low	High	Unclear	High	High	Low	Low	Low	Low
Yuan S, 2023	Yuan 2023	D	Low	High	High	High	High	Low	Low	High	High

AI-ECG, Artificial Intelligence-enhanced Electrocardiography; ASCO, American Society of Clinical Oncology; BMI, body mass index; CARE-BMT, Cardiovascular Registry in Bone Marrow Transplantation; CCSS, Childhood Cancer Survivors Study; CRS, cardiotoxicity risk score; D, development; ECG, electrocardiogram; EKZ/AMC, Emma Children's Hospital/Academic Medical Center; EV, external validation; FHCRC, Fred Hutchinson Cancer Research Center; HCT-CI, Hematopoietic Cell Transplantation-specific Comorbidity Index; HFA-ICOS, Heart Failure Association - International Cardio-Oncology Society; NWTS, National Wilms Tumor Study; PCP-HF, Pooled Cohort equations to Prevent HF; QRISK3, Cardiovascular Disease Risk Score; SCORE-(OP), Systematic COronary Risk Evaluation – (Older Persons); SJLIFE, St. Jude Lifetime Cohort Study.

Supplemental Table 15. PROBAST signaling question scores per study/model: Y/PY — yes/probably yes; NI — no information; N/PN — no/probably not.

Study	Model	Aim	Participants		Predictors			Outcomes						Analysis								
			1.1	1.2	2.1	2.2	2.3	3.1	3.2	3.3	3.4	3.5	3.6	4.1	4.2	4.3	4.4	4.5*	4.6	4.7	4.8*	4.9*
Abdel-Qadir H, 2019	Abdel-Qadir 2019	D	PY	PN	PN	NI	Y	N	N	N	PN	NI	Y	Y	PY	Y	Y	Y	Y	Y	PY	Y
Abiodun A, 2024	QRISK3	EV	Y	PN	Y	PN	PY	Y	Y	N	Y	Y	Y	Y	PY	PN	N	NA	Y	N	NA	NA
Advani P, 2016	Romond 2012	EV	PY	PY	PN	NI	Y	PY	PN	Y	PN	NI	NI	Y	NI	Y	NI	NA	PN	N	NA	NA
Armenian S, 2018	Armenian 2018 [full model]	D	Y	PN	Y	NI	Y	NI	PY	Y	PN	NI	NI	Y	PN	N	N	N	Y	N	N	Y
		EV	PY	PN	Y	NI	Y	PY	NI	Y	N	NI	NI	Y	PN	N	N	NA	Y	N	NA	NA
Astarita A, 2021	CFZ CVAE score	D	Y	PN	PY	PY	Y	PN	PN	Y	PN	NI	Y	PN	NI	Y	Y	N	PN	PY	PY	Y
Battisti, 2021	HFA-ICOS [anti-Her2]	EV	Y	PY	PN	NI	Y	NI	NI	Y	N	NI	Y	Y	PN	Y	NI	NA	N	N	NA	NA
Carballo-Folgoso L, 2021	CARDIOSOR SCALE	D	Y	PN	PN	PY	Y	NI	NI	N	NI	NI	Y	N	PN	PY	NI	N	Y	N	N	NI
Caro-Codón J, 2022	SCORE	EV	Y	PN	PY	PY	PY	PY	Y	Y	PY	NI	Y	Y	N	Y	PN	NA	PN	N	NA	NA
Chaix MA, 2020	Chaix 2020 [clinical]	D	N	PN	PY	PN	Y	NI	Y	Y	PN	NI	PY	Y	Y	Y	NI	PY	N	PN	Y	PY
	Chaix 2020 [genetic]	D	N	PN	Y	PN	Y	NI	Y	Y	PN	NI	PY	N	Y	Y	NI	PY	N	PN	Y	PY
	Chaix 2020 [both]	D	N	PN	PY	PN	Y	NI	Y	Y	PN	NI	PY	N	Y	Y	NI	PY	N	PN	Y	PY
Chang WT, 2022	Chang 2022 [CTRCD]	D	Y	PY	Y	PY	Y	Y	Y	Y	Y	NI	Y	N	Y	PN	NI	PY	N	PN	N	PY
	Chang 2022 [HFrEF]	D	Y	PY	Y	PY	Y	Y	Y	Y	Y	NI	Y	N	Y	PN	NI	PY	N	PN	N	PY
Chen Y, 2020	Chen 2020 [age 20]	D	PY	PY	PN	PY	Y	PN	Y	Y	PY	PN	Y	Y	PN	N	N	PY	Y	N	N	N
	Chen 2020 [age 25]	D	PY	PY	PN	PY	Y	PN	Y	Y	PY	PN	Y	Y	PN	N	N	PY	Y	N	N	N

	Chen 2020 [age 30]	D	PY	PY	PN	PY	Y	PN	Y	Y	PY	PN	Y	PN	PN	N	N	PY	Y	N	N	N
	Chen 2020 [age 35]	D	PY	PY	PN	PY	Y	PN	Y	Y	PY	PN	Y	PN	PN	N	N	PY	Y	N	N	N
Chow E, 2015	Chow 2015 [simple]	D	PY	PY	PN	PY	Y	PN	Y	Y	PY	PN	Y	Y	PN	N	N	PY	PN	N	Y	Y
		EV<br>EKZ/AMC	PY	PY	PN	PY	Y	PN	Y	Y	PY	PN	Y	Y	PN	N	N	PY	PN	N	Y	Y
		EV<br>NWTS	PY	PY	PN	PY	Y	PN	Y	Y	PY	PN	Y	Y	PN	N	N	PY	PN	N	Y	Y
		EV<br>SJLIFE	PY	PY	N	PN	Y	Y	Y	Y	PN	NI	Y	Y	PY	N	N	NA	PY	N	NA	NA
	Chow 2015 [standard]	D	PY	PY	N	PN	Y	Y	Y	Y	PN	NI	Y	Y	PY	N	N	NA	PY	N	NA	NA
		EV<br>EKZ/AMC	PY	PY	N	PN	Y	Y	Y	Y	PN	NI	Y	Y	PY	N	N	NA	PY	N	NA	NA
		EV<br>NWTS	PY	PN	PY	PY	Y	PN	N	Y	PN	NI	Y	Y	PN	N	N	NA	PY	N	NA	NA
		EV<br>SJLIFE	PY	PN	PY	PY	Y	PN	N	Y	PN	NI	Y	Y	PN	N	N	NA	PY	N	NA	NA
	Chow 2015 [heart dose]	D	PY	PY	PY	PY	Y	PN	Y	Y	PN	NI	Y	Y	PY	N	N	NA	PY	N	NA	NA
		EV<br>EKZ/AMC	PY	PY	PY	PY	Y	PN	Y	Y	PN	NI	Y	Y	PY	N	N	NA	PY	N	NA	NA
Cronin M, 2023	HFA-ICOS [anti-Her2]	EV	Y	PY	NI	NI	N	PY	NI	Y	PN	NI	Y	Y	NI	Y	N	NA	N	N	NA	NA
De Vries S, 2022	De Vries 2022 [simple]	D	Y	PY	PY	PY	Y	PN	Y	Y	PN	NI	NI	Y	PN	N	N	Y	Y	Y	PN	NI
	De Vries 2022 [dose]	D	Y	PY	PY	PY	Y	PN	Y	Y	PN	NI	NI	Y	PN	N	N	Y	Y	Y	Y	NI
Di Lisi D, 2024	HFA-ICOS [anthracyclines]	EV	Y	PY	PY	PY	N	NI	Y	Y	NI	N	Y	PN	PN	PN	NI	NA	PN	N	NA	NA
Diamond A, 2021	Diamond 2021	D	Y	PN	PN	NI	Y	PN	N	Y	PN	NI	Y	Y	PN	PN	N	N	Y	N	N	Y
Doukas PG, 2022	Kang 2019	EV	Y	PN	Y	Y	Y	Y	Y	Y	Y	NI	Y	Y	PY	Y	NI	NA	N	N	NA	NA
Dranitsaris G, 2008	Dranitsaris 2008	D	PY	PN	PY	PY	Y	Y	Y	Y	Y	NI	NI	N	PN	Y	NI	N	N	N	PN	Y

Ezaz G, 2014	Ezaz 2014	D	PY	PN	PN	NI	Y	PN	PY	Y	PN	NI	Y	Y	PN	Y	Y	N	PN	N	N	Y
Fernando F, 2024	HFA-ICOS [BCR-ABL]	EV	Y	PN	Y	NI	N	PN	Y	PN	PN	N	Y	Y	PN	PN	N	NA	N	N	NA	NA
Fogarassy G, 2019	Fogarassy 2019	D	PY	PN	PN	NI	Y	PN	N	Y	PN	NI	NI	Y	PN	Y	N	PY	N	N	N	N
Gomez-Vecino A, 2023	Gomez-Vecino 2023	D	PY	PY	PY	PY	Y	PN	NI	Y	PN	NI	NI	NI	PY	NI	NI	NI	N	N	Y	NI
Gunturkun F, 2021	Gunturkun 2021 [clinical]	D	PY	PY	PY	PY	Y	NI	Y	Y	NI	NI	Y	Y	Y	PN	Y	PY	N	PN	Y	PY
	Gunturkun 2021 [ECG]	D	PY	PY	Y	PY	Y	NI	Y	Y	NI	NI	Y	N	Y	PN	Y	PY	N	PN	Y	PY
	Gunturkun 2021 [both]	D	PY	PY	PY	PY	Y	NI	Y	y	NI	NI	Y	N	Y	PN	Y	PY	N	PN	Y	PY
Heilbroner SP, 2021	Heilbroner 2021 [full]	D	PY	PN	Y	PY	PY	PN	Y	Y	PN	PY	Y	N	Y	Y	PY	PY	PN	N	N	PY
	Heilbroner 2021 [simplified]	D	PY	PY	Y	PY	PY	PN	Y	Y	PN	PY	Y	Y	Y	Y	PY	PY	PN	N	N	PY
Jacobs J, 2022	Jacobs 2022 [1]	D	Y	N	NI	NI	Y	NI	Y	Y	NI	NI	NI	PY	NI	Y	PY	PN	N	N	PN	NI
	Jacobs 2022 [1]	D	Y	N	NI	NI	Y	NI	Y	Y	NI	NI	NI	PY	NI	Y	PY	PN	N	N	PN	NI
	ASCO risk score	EV	Y	N	NI	NI	Y	NI	Y	Y	NI	NI	NI	Y	NI	Y	N	NA	N	N	NA	NA
	CRS (Herrmann, 2014)	EV	Y	N	NI	NI	Y	NI	Y	N	NI	NI	NI	Y	NI	Y	PY	NA	N	N	NA	NA
Jones KA, 2020	Jones 2020 [1]	D	Y	PY	Y	NI	Y	Y	Y	Y	Y	NI	Y	N	PY	PN	NI	PN	N	N	NI	NI
	Jones 2020 [2]	D	Y	PY	Y	NI	Y	Y	Y	Y	Y	NI	Y	N	PY	PN	NI	PN	N	N	NI	NI
	Jones 2020 [RF]	D	Y	PY	Y	NI	Y	Y	Y	Y	Y	NI	Y	N	PY	PN	NI	PN	N	N	PN	NI
Kang Y, 2019	Kang 2019	D	Y	PN	PN	Y	Y	Y	Y	Y	Y	NI	Y	N	PY	N	Y	N	PY	N	PN	Y
Kim DY, 2021	CHEMO-RADIAT	D	Y	PN	PY	NI	Y	PY	PN	N	PN	N	NI	N	N	N	N	NI	PN	N	PY	NI

Kotwinski P , 2016	Kotwinski 2016	D	Y	PN	PY	PY	Y	Y	N	Y	Y	Y	Y	N	NI	N	N	N	N	N	Y	NI
Law Q, 2017	Framingham	EV	Y	PN	PY	NI	Y	PN	PY	Y	PN	NI	NI	Y	PY	N	N	NA	PN	N	NA	NA
Leerink JM, 2021	Leerink 2021	D	Y	PY	PY	NI	Y	PY	PY	Y	Y	NI	Y	N	Y	Y	Y	NI	PN	PY	PN	NI
		EV	PY	PY	PY	NI	Y	PY	PY	Y	Y	Y	Y	Y	PY	Y	Y	NA	PN	PY	NA	NA
Li C, 2022	Li 2022 [XGBoost]	D	PY	Y	Y	Y	NI	PN	Y	NI	PN	NI	Y	NI	Y	Y	PY	PY	N	PN	Y	PY
Liu B, 2022	Liu B 2022	D	Y	PY	PY	NI	Y	Y	PY	Y	Y	NI	Y	Y	PY	N	N	N	N	PN	N	Y
Liu X, 2022	ABSDELL	D	Y	PN	NI	NI	Y	PN	Y	Y	PN	NI	NI	PY	PY	N	PN	Y	PN	Y	PY	PY
	HFA-ICOS	EV	Y	PN	NI	NI	Y	PN	Y	Y	PN	NI	NI	Y	NI	N	N	NA	PN	N	NA	NA
Liu Z, 2022	Liu Z 2022	D	Y	PY	PY	NI	Y	Y	NI	Y	PY	NI	Y	PY	N	N	PN	PY	N	PN	PN	NI
Mccracken C, 2024	QRISK3	EV	PY	Y	Y	PY	Y	PN	Y	N	N	NI	Y	Y	PY	PY	Y	NA	Y	Y	NA	NA
	Framingham (BMI)	EV	PY	Y	Y	PY	Y	PN	Y	Y	N	NI	Y	Y	PY	PY	Y	NA	Y	Y	NA	NA
	Framingham (blood lipids)	EV	PY	Y	Y	PY	Y	PN	Y	Y	N	NI	Y	Y	PY	PY	Y	NA	Y	Y	NA	NA
	SCORE2 / SCORE-OP	EV	PY	Y	Y	PY	Y	PN	Y	Y	N	NI	Y	Y	PY	PY	Y	NA	Y	Y	NA	NA
	PCP-HF	EV	PY	Y	Y	PY	Y	PN	Y	Y	N	NI	Y	Y	PY	PY	Y	NA	Y	Y	NA	NA
Mery B, 2022	Abdel-Qadir 2019	EV	Y	PN	PY	NI	Y	PN	NI	N	PN	NI	Y	Y	NI	Y	NI	NA	PN	Y	NA	NA
Moey MYY, 2019	Ezaz 2014	EV	Y	PY	PN	NI	Y	PN	PN	Y	N	NI	Y	Y	NI	Y	NI	NA	NI	N	NA	NA
Nguyen QT, 2024	Nguyen 2024	D	PY	PN	Y	PY	PY	PN	Y	N	PN	NI	Y	NI	Y	PY	NI	PY	N	N	Y	PY
		EV	PY	PN	Y	PY	PY	PN	Y	N	PN	NI	Y	NI	Y	PY	NI	NA	N	PN	NA	NA
Oikonomou E. K., 2024	AI-ECG (Sangha, 2023)	EV	Y	PN	Y	Y	Y	PN	NI	Y	PN	Y	y	PY	Y	PN	NI	NA	Y	N	NA	NA
Otchere P, 2023	Otchere 2023	D	Y	PN	PY	NI	Y	PN	Y	Y	PN	NI	Y	N	NI	Y	N	Y	N	N	N	NI
Ozturk C, 2021	Ozturk 2021	D	Y	PY	PY	NI	Y	Y	Y	Y	Y	Y	Y	N	PY	Y	NI	N	N	N	N	PN

Pohl J, 2021	ECG score (Chatterjee, 2020)	EV	Y	N	PY	NI	Y	PN	Y	Y	PN	NI	Y	Y	PY	Y	NI	NA	N	N	NA	NA
Rivero-Santana B, 2024	HFA-ICOS [anthracyclines]	EV	Y	PY	Y	PY	Y	Y	Y	Y	Y	NI	Y	Y	PN	PN	N	NA	N	Y	NA	NA
Romond EH, 2012	Romond 2012	D	PY	PY	PY	Y	Y	Y	NI	Y	Y	NI	Y	Y	NI	Y	NI	N	Y	PY	PN	PY
Rushton M, 2017	Ezaz 2014	EV	Y	N	PY	NI	Y	NI	PN	Y	PN	NI	Y	Y	NI	Y	NI	NA	NI	N	NA	NA
Shibata T, 2023	HFA-ICOS [unspecified]	EV	Y	N	PY	NI	Y	PY	PN	Y	PY	NI	Y	Y	NI	Y	NI	NA	PN	N	NA	NA
Stefanini B, 2024	Carballo-Folgoso 2021 [CARDIOSOR]	EV	PY	PN	Y	PY	Y	PN	Y	PY	PN	NI	Y	Y	PN	PY	NI	NA	Y	N	NA	NA
	HFA-ICOS [VEGF inhibitors]	EV	PY	PN	Y	PY	N	PN	Y	Y	PN	NI	Y	Y	PN	PY	NI	NA	Y	N	NA	NA
Sun Y, 2021	Sun 2021	D	Y	PN	PY	NI	Y	NI	Y	Y	NI	PY	Y	N	N	Y	Y	N	N	N	N	Y
Suntheralingam S, 2022	HFA-ICOS [anti-Her2]	EV	Y	PN	PN	NI	Y	PN	Y	Y	N	NI	Y	Y	NI	Y	NI	NA	N	Y	NA	NA
	Romond 2012	EV	Y	PY	PN	NI	Y	PN	Y	Y	N	NI	Y	Y	NI	Y	NI	NA	N	Y	NA	NA
	Ezaz 2014	EV	Y	PY	PN	NI	Y	PN	Y	Y	N	NI	Y	Y	NI	Y	NI	NA	N	Y	NA	NA
Tini G, 2021	HFA-ICOS [anthracyclines]	EV	Y	N	NI	NI	N	N	Y	Y	PN	NI	NI	Y	NI	Y	N	NA	PN	N	NA	NA
	HFA-ICOS [anti-Her2]	EV	Y	N	NI	NI	N	N	N	Y	PN	NI	NI	Y	NI	Y	N	NA	PN	N	NA	NA
Upshaw JN, 2019	Upshaw 2019	D	PY	PY	PY	NI	Y	Y	PY	Y	N	PN	Y	PN	Y	Y	Y	Y	N	N	PN	NI
Vasbinder A, 2024	CARE-BMT	D	Y	Y	Y	NI	Y	PY	Y	N	NI	NI	PY	Y	N	PY	N	N	Y	N	Y	N
		EV	Y	Y	Y	NI	Y	PY	Y	N	NI	NI	PY	Y	N	PY	N	NA	Y	N	NA	NA
	Armenian 2018 [full model]	EV	Y	Y	Y	NI	Y	PY	Y	Y	NI	NI	PY	Y	N	PY	N	NA	Y	N	NA	NA

	HCT-CI	EV	Y	Y	Y	NI	Y	PY	Y	N	NI	NI	PY	Y	N	PY	N	NA	Y	N	NA	NA
Yagi R, 2024	Yagi 2024 [Model 1]	D	PY	PN	PN	PY	Y	PN	Y	Y	N	PY	Y	NI	Y	Y	PY	PY	Y	N	N	NI
Yu AF, 2023	Yu 2024	D	Y	PY	PN	NI	Y	NI	Y	Y	PN	NI	Y	Y	N	PN	NI	N	PN	Y	Y	PN
Yuan S, 2023	Yuan 2023	D	Y	PY	PN	NI	Y	PN	PN	Y	PN	NI	Y	Y	N	Y	NI	N	PN	Y	N	PN

AI-ECG, Artificial Intelligence-enhanced Electrocardiography; ASCO, American Society of Clinical Oncology; BMI, body mass index; CARE-BMT, Cardiovascular Registry in Bone Marrow Transplantation; CCSS, Childhood Cancer Survivors Study; CRS, cardiotoxicity risk score; D, development; ECG, electrocardiogram; EKZ/AMC, Emma Children's Hospital/Academic Medical Center; EV, external validation; FHCRC, Fred Hutchinson Cancer Research Center; HCT-CI, Hematopoietic Cell Transplantation-specific Comorbidity Index; HFA-ICOS, Heart Failure Association - International Cardio-Oncology Society; NWTS, National Wilms Tumor Study; PCP-HF, Pooled Cohort equations to Prevent HF; QRISK3, Cardiovascular Disease Risk Score; SCORE-(OP), Systematic COronary Risk Evaluation – (Older Persons); SJLIFE, St. Jude Lifetime Cohort Study.

*Questions 4.5, 4.8, and 4.9 are not applicable (N/A) to external validation studies.

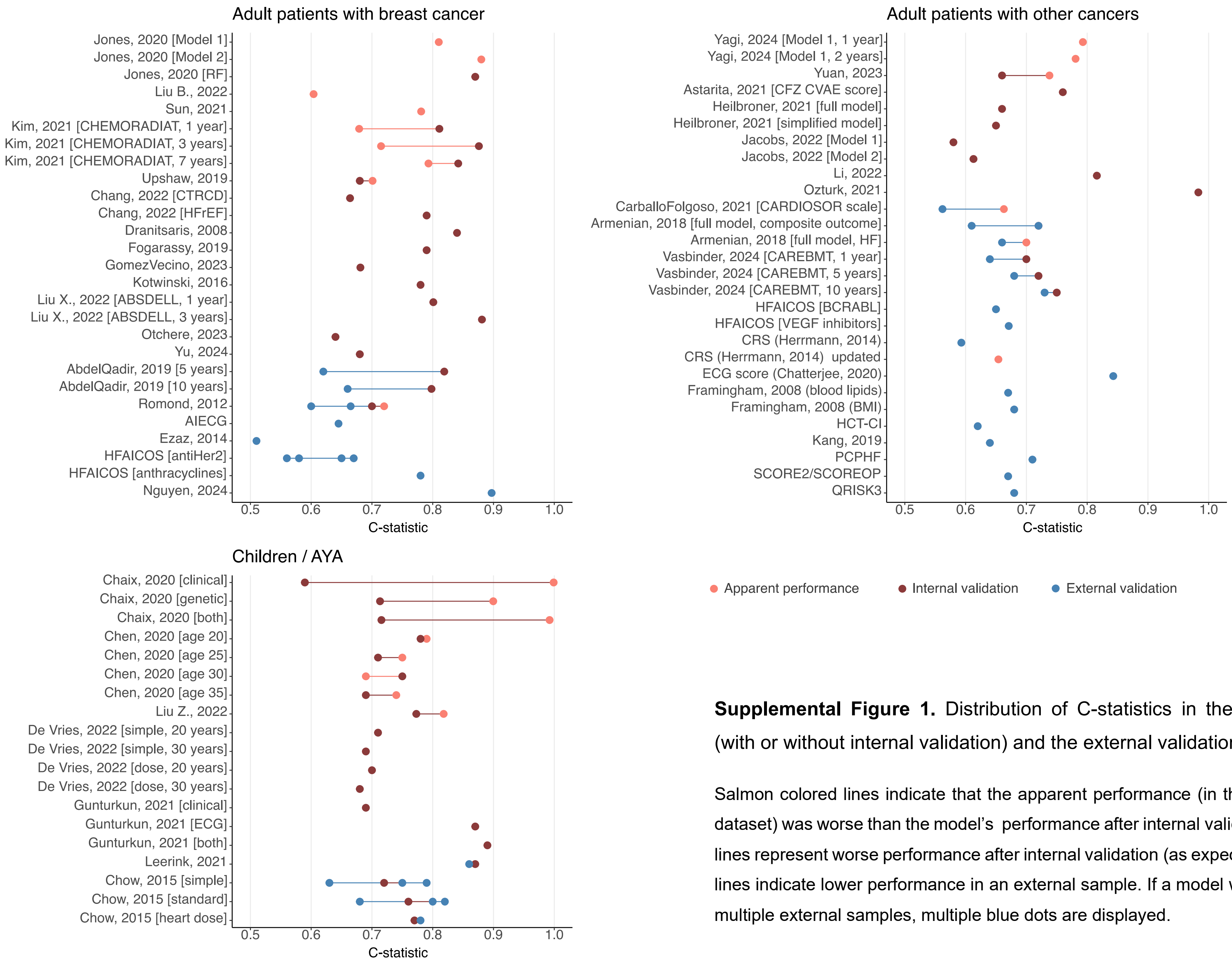

Supplemental Figure 1. Distribution of C-statistics in the development (with or without internal validation) and the external validation samples.

Salmon colored lines indicate that the apparent performance (in the development dataset) was worse than the model's performance after internal validation. Dark red lines represent worse performance after internal validation (as expected), while blue lines indicate lower performance in an external sample. If a model was validated in multiple external samples, multiple blue dots are displayed.

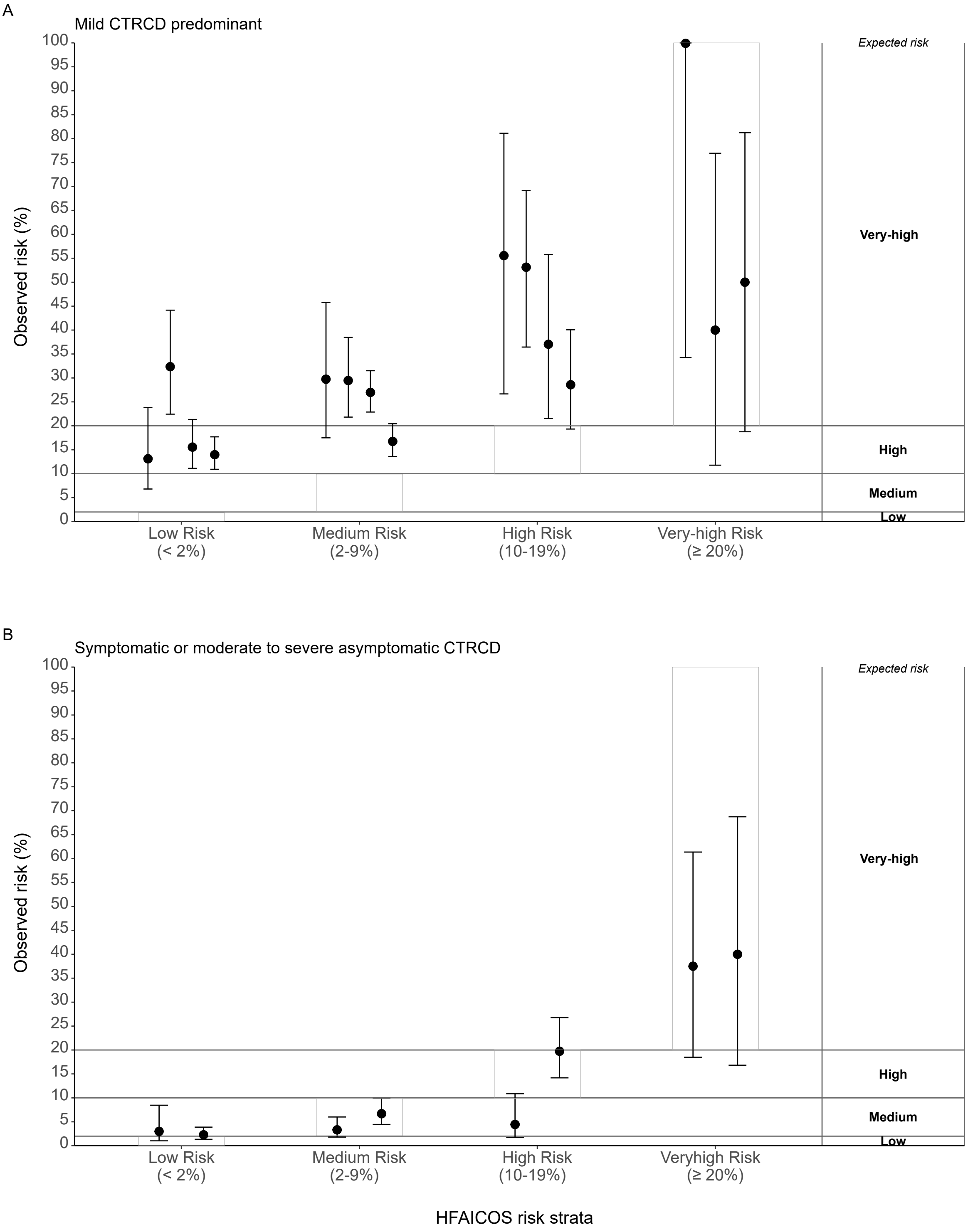

Supplemental Figure 2. Calibration of the HFA-ICOS risk assessment tool across external validation studies targeting CTRCD. A) studies where mild asymptomatic CTRCD events were predominant (n=4); B) studies where symptomatic or moderate to severe asymptomatic CTRCD events were predominant (n=2). Horizontal dashed lines mark the expected risk thresholds between HFA-ICOS risk group[114]: low (< 2%), medium (2-9%), high (10-19%), and very-high (≥ 20%). Grey areas show the expected risk range for each group. Black dots (with confidence intervals) represent the observed proportion of patients with the outcome (y axis) within each risk stratum (x axis) per study. Dots falling above the corresponding expected range (grey area) indicate underestimation of risk by the model; those below indicates an overestimation. Studies targeting other cardiovascular events (n=4) where excluded from this plot (see **Supplemental Table 8**).

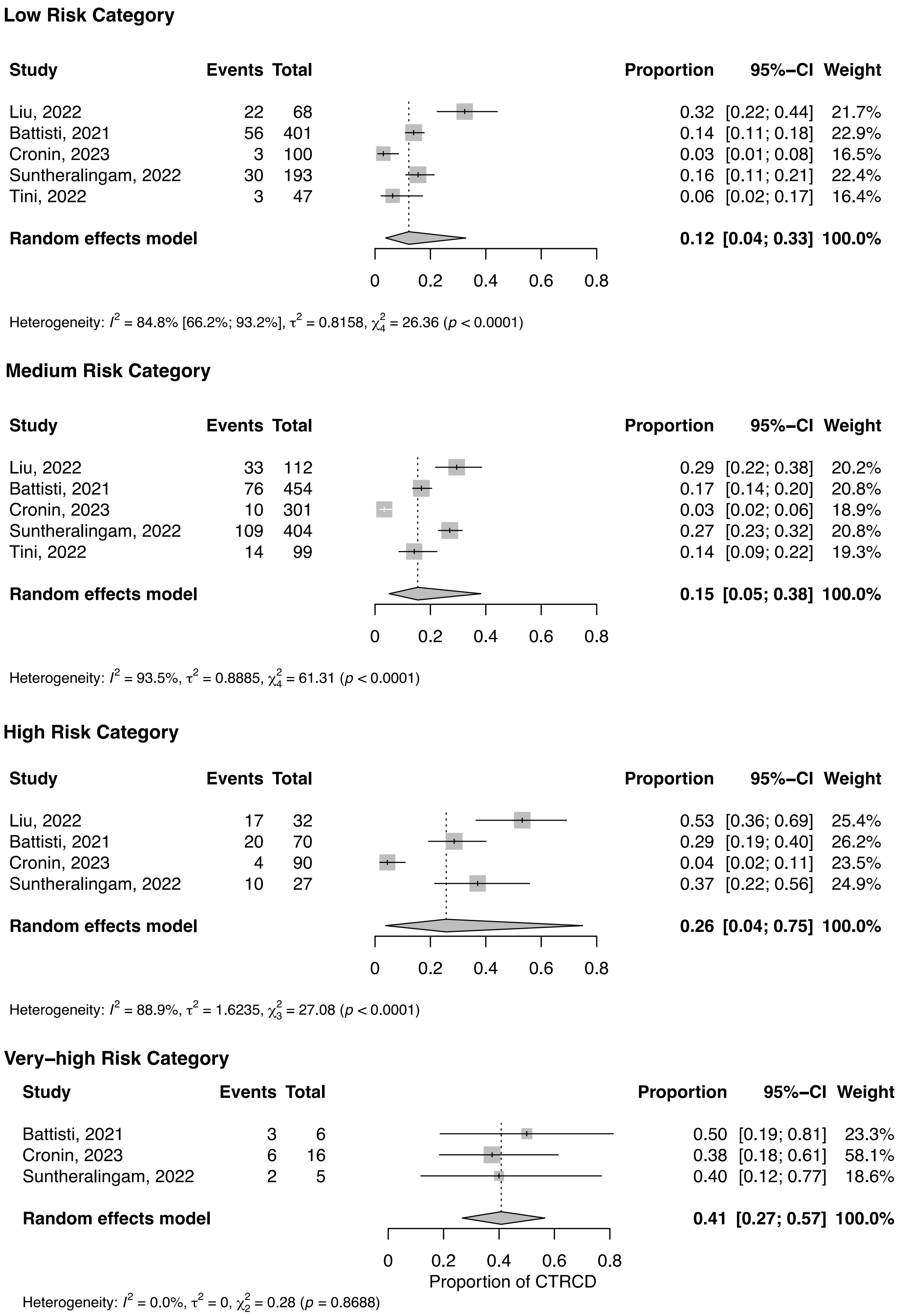

Supplemental Figure 3. Forest plots of the meta-analyses illustrating observed risks across all external validation studies in patients treated with Her2-targeted therapies per HFA-ICOS risk strata. CI, confidence interval.

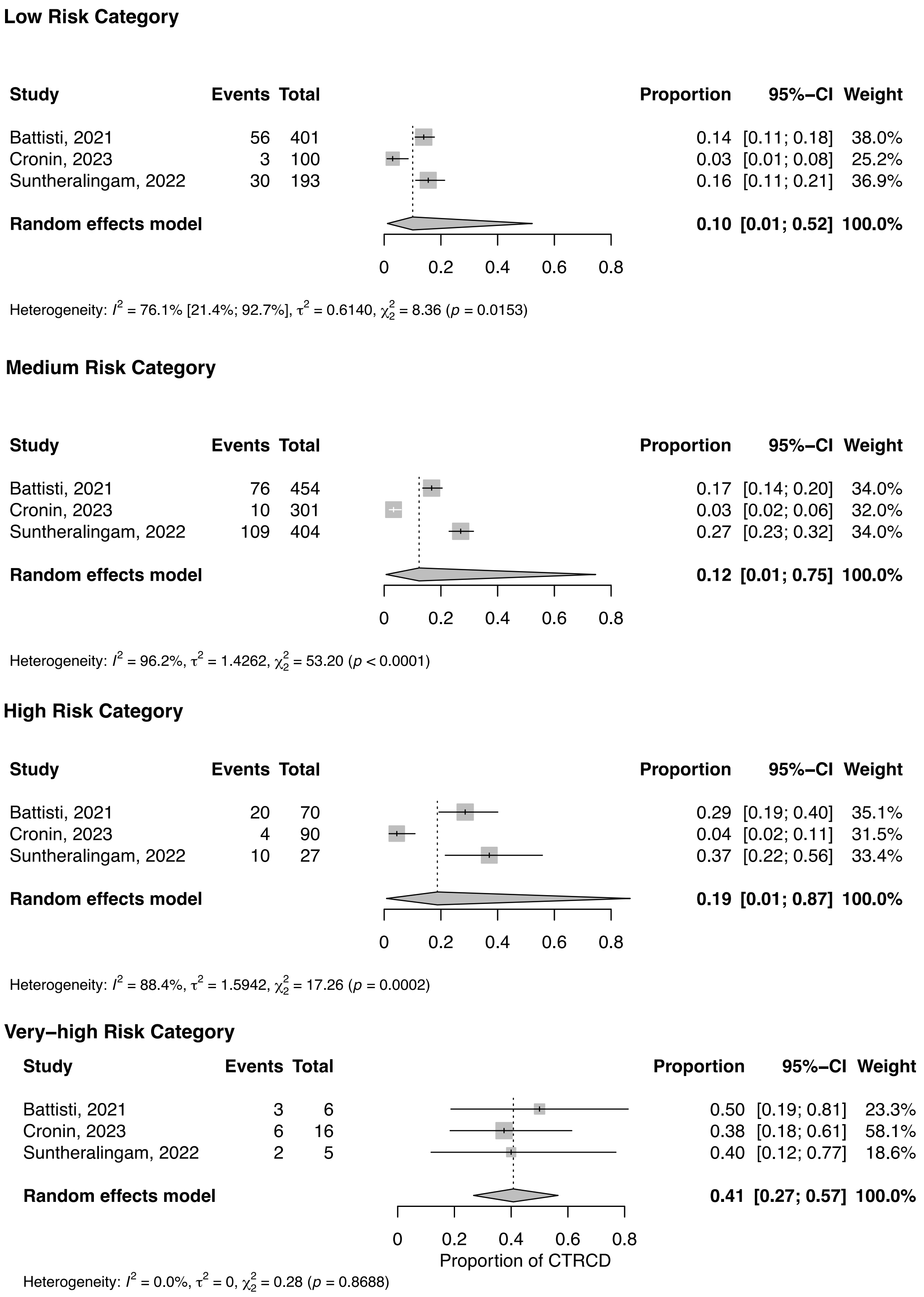

Supplemental Figure 4. Forest plots of the meta-analyses of observed risks across HFA-ICOS risk strata, after excluding studies with high applicability concerns. CI, confidence interval.

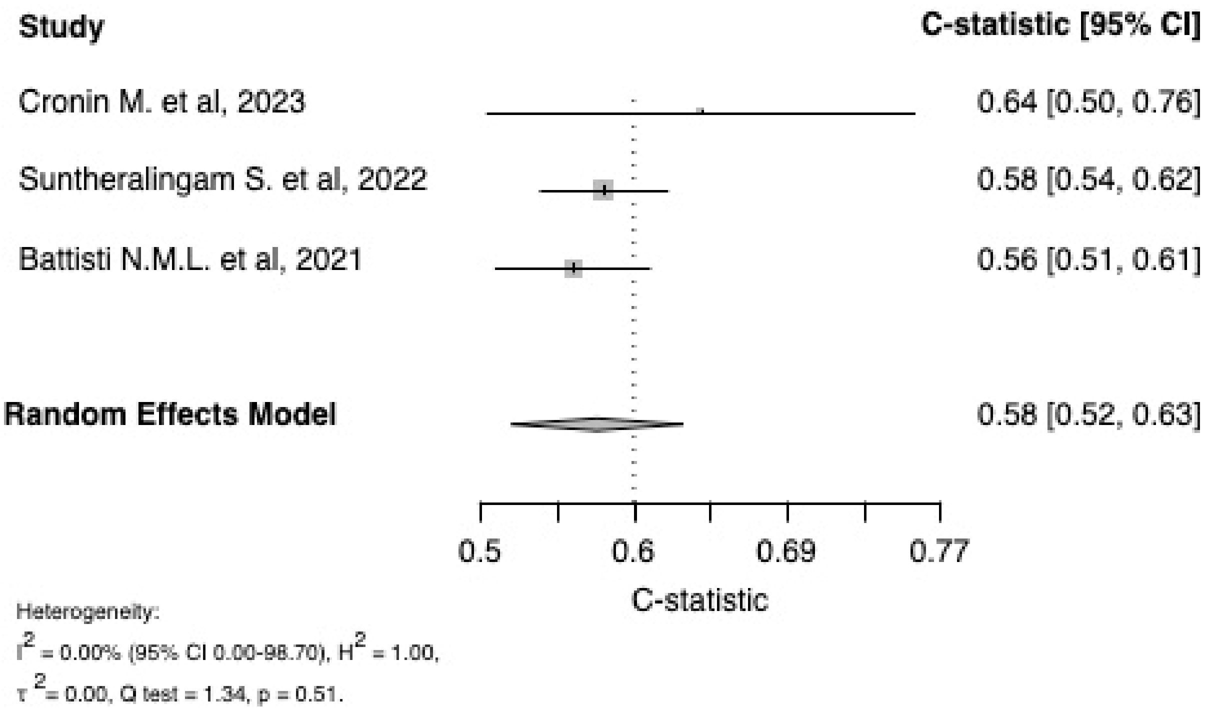

Supplemental Figure 5. Forest plot of the meta-analysis of C-statistic measures across external validation studies of the HFA-ICOS risk assessment tool in patients receiving Her2-targeted therapies, after excluding studies with high applicability concerns. CI, confidence interval.

References:

1.Heilbroner SP, Few R, Neilan TG, et al. Predicting cardiac adverse events in patients receiving immune checkpoint inhibitors: a machine learning approach. *J Immunother Cancer* 2021;9:e002545. doi: 10.1136/jitc-2021-002545

2.Li C, Chen L, Chou C, Ngorsuraches S, Qian J. Using Machine Learning Approaches to Predict Short-Term Risk of Cardiotoxicity Among Patients with Colorectal Cancer After Starting Fluoropyrimidine-Based Chemotherapy. *Cardiovascular Toxicology* 2022;22:130-140. doi: 10.1007/s12012-021-09708-4

3.Abdel-Qadir H, Thavendiranathan P, Austin PC, et al. Development and validation of a multivariable prediction model for major adverse cardiovascular events after early stage breast cancer: a population-based cohort study. *Eur Heart J* 2019;40:3913-3920. doi: 10.1093/eurheartj/ehz460

4.Abiodun A, Shawe-Taylor M, Tyebally S, et al. Predicting cardiovascular events with fluoropyrimidine chemotherapy using a standard cardiovascular risk calculator. *ESC Heart Fail* 2024;11:3041-3051. doi: https://doi.org/10.1002/ehf2.14879

5.Advani PP, Ballman KV, Dockter TJ, Colon-Otero G, Perez EA. Long-Term Cardiac Safety Analysis of NCCTG N9831 (Alliance) Adjuvant Trastuzumab Trial. *J Clin Oncol* 2016;34:581-587. doi: 10.1200/jco.2015.61.8413

6.Armenian SH, Yang D, Teh JB, et al. Prediction of cardiovascular disease among hematopoietic cell transplantation survivors. *Blood Adv* 2018;2:1756-1764. doi: 10.1182/bloodadvances.2018019117

7.Astarita A, Mingrone G, Airale L, et al. Multiple Myeloma Patients Undergoing Carfilzomib: Development and Validation of a Risk Score for Cardiovascular Adverse Events Prediction. *Cancers (Basel)* 2021;13 doi: 10.3390/cancers13071631

8.Battisti NML, Andres MS, Lee KA, et al. Incidence of cardiotoxicity and validation of the Heart Failure Association-International Cardio-Oncology Society risk stratification tool in patients treated with trastuzumab for HER2-positive early breast cancer. *Breast Cancer Res Treat* 2021;188:149-163. doi: 10.1007/s10549-021-06192-w

9.Carballo-Folgoso L, Álvarez-Velasco R, Lorca R, et al. Evaluation of cardiovascular events in patients with hepatocellular carcinoma treated with sorafenib in the clinical practice. The CARDIO-SOR study. *Liver Int* 2021;41:2200-2211. doi: 10.1111/liv.14941

10.Cronin M, Crowley A, Davey MG, et al. Heart Failure Association-International Cardio-Oncology Society Risk Score Validation in HER2-Positive Breast Cancer. *J Clin Med* 2023;12:1278.

11.Diamond A, Ayyappan S, Cao S, et al. Risk factors for cardiovascular events and mortality in patients diagnosed with diffuse large B-cell lymphoma and treated with anthracyclines. *Hematol Oncol* 2022;40:626-636. doi: 10.1002/hon.3034

12.Fernando F, Andres MS, Claudiani S, et al. Cardiovascular events in CML patients treated with Nilotinib: validation of the HFA-ICOS baseline risk score. *Cardiooncology* 2024;10:42. doi: 10.1186/s40959-024-00245-x

13.Kim DY, Park MS, Youn JC, et al. Development and Validation of a Risk Score Model for Predicting the Cardiovascular Outcomes After Breast Cancer Therapy: The CHEMO-RADIAT Score. *J Am Heart Assoc* 2021;10:e021931. doi: 10.1161/jaha.121.021931

14.Law W, Johnson C, Rushton M, Dent S. The Framingham Risk Score Underestimates the Risk of Cardiovascular Events in the HER2-Positive Breast Cancer Population. *Current Oncology* 2017;24:348-353.

15.Liu B, Guan X, Wang Y, et al. Clinical and genetic predictions of early-onset cardiac toxicity in adjuvant chemotherapy for breast cancer. *Future Oncol* 2022;18:2127-2139. doi: 10.2217/fon-2021-1021

16.McCracken C, Condurache D-G, Szabo L, et al. Predictive Performance of Cardiovascular Risk Scores in Cancer Survivors From the UK Biobank. *J Am Coll Cardiol CardioOnc* 2024;6:575-588. doi: 10.1016/j.jaccao.2024.05.015

17.Mery B, Rowinski E, Rivier C, et al. Cardiovascular Diseases Following Breast Cancer: Towards a Case-by-Case Assessment Through a Prediction Risk Score Model in 943 Patients. *Am J Clin Oncol* 2022;45:155-160. doi: 10.1097/coc.0000000000000904

18. Nguyen QTN, Phan PT, Lin SJ, et al. Machine-Learning Based Risk Assessment for Cancer Therapy-Related Cardiac Adverse Events Among Breast Cancer Patients. *Stud Health Technol Inform* 2024;310:1006-1010. doi: 10.3233/shti231116
19. Romond EH, Jeong JH, Rastogi P, et al. Seven-year follow-up assessment of cardiac function in NSABP B-31, a randomized trial comparing doxorubicin and cyclophosphamide followed by paclitaxel (ACP) with ACP plus trastuzumab as adjuvant therapy for patients with node-positive, human epidermal growth factor receptor 2-positive breast cancer. *J Clin Oncol* 2012;30:3792-3799. doi: 10.1200/jco.2011.40.0010
20. Shibata T, Nohara S, Morikawa N, et al. Cardiovascular adverse events and prognosis in patients with haematologic malignancies and breast cancer receiving anticancer agents: Kurume-CREO Registry insights. *Eur J Prev Cardiol* 2023;30:1941-1949. doi: 10.1093/eurjpc/zwad210
21. Stefanini B, Tovoli F, Trevisani F, et al. Prediction of cardiovascular risk in patients with hepatocellular carcinoma receiving anti-angiogenic drugs: lessons from sorafenib. *Intern Emerg Med* 2024;19:1151-1160. doi: 10.1007/s11739-024-03578-8
22. Tini G, Cuomo A, Battistoni A, et al. Baseline cardio-oncologic risk assessment in breast cancer women and occurrence of cardiovascular events: The HFA/ICOS risk tool in real-world practice. *Int J Cardiol* 2022;349:134-137. doi: 10.1016/j.ijcard.2021.11.059
23. Vasbinder A, Catalan T, Anderson E, et al. Cardiovascular Risk Stratification of Patients Undergoing Hematopoietic Stem Cell Transplantation: The CARE-BMT Risk Score. *J Am Heart Assoc* 2024;13:e033599. doi: doi:10.1161/JAHA.123.033599
24. Yuan S, Zhou JY, Yang BZ, et al. Prediction of cardiovascular adverse events in newly diagnosed multiple myeloma: Development and validation of a risk score prognostic model. *Front Oncol* 2023;13:1043869. doi: 10.3389/fonc.2023.1043869
25. Caro-Codón J, López-Fernández T, Álvarez-Ortega C, et al. Cardiovascular risk factors during cancer treatment. Prevalence and prognostic relevance: insights from the CARDIOTOX registry. *Eur J Prev Cardiol* 2022;29:859-868. doi: 10.1093/eurjpc/zwaa034
26. Chaix MA, Parmar N, Kinnear C, et al. Machine Learning Identifies Clinical and Genetic Factors Associated With Anthracycline Cardiotoxicity in Pediatric Cancer Survivors. *J Am Coll Cardiol CardioOnc* 2020;2:690-706. doi: 10.1016/j.jaccao.2020.11.004
27. Chang WT, Liu CF, Feng YH, et al. An artificial intelligence approach for predicting cardiotoxicity in breast cancer patients receiving anthracycline. *Arch Toxicol* 2022;96:2731-2737. doi: 10.1007/s00204-022-03341-y
28. Chen Y, Chow EJ, Oeffinger KC, et al. Traditional Cardiovascular Risk Factors and Individual Prediction of Cardiovascular Events in Childhood Cancer Survivors. *J Natl Cancer Inst* 2019;112:256-265. doi: 10.1093/jnci/djz108
29. Chow EJ, Chen Y, Kremer LC, et al. Individual prediction of heart failure among childhood cancer survivors. *J Clin Oncol* 2015;33:394-402. doi: 10.1200/jco.2014.56.1373
30. de Vries S, Haaksma ML, Jóźwiak K, et al. Development and Validation of Risk Prediction Models for Coronary Heart Disease and Heart Failure After Treatment for Hodgkin Lymphoma. *J Clin Oncol* 2023;41:86-95. doi: 10.1200/jco.21.02613

31.Di Lisi D, Madaudo C, Faro DC, et al. The added value of the HFA/ICOS score in the prediction of chemotherapy-related cardiac dysfunction in breast cancer. *J Cardiovasc Med (Hagerstown)* 2024;25:218-224. doi: 10.2459/jcm.0000000000001589
32.Doukas PG, Cascino GJ, Meng Z, et al. External validation of a heart failure risk score in patients with acute myeloid leukemia. *Leuk Lymphoma* 2023;64:445-453. doi: 10.1080/10428194.2022.2140289
33.Dranitsaris G, Rayson D, Vincent M, et al. The development of a predictive model to estimate cardiotoxic risk for patients with metastatic breast cancer receiving anthracyclines. *Breast Cancer Res Treat* 2008;107:443-450. doi: 10.1007/s10549-007-9803-5
34.Ezaz G, Long JB, Gross CP, Chen J. Risk prediction model for heart failure and cardiomyopathy after adjuvant trastuzumab therapy for breast cancer. *J Am Heart Assoc* 2014;3:e000472. doi: 10.1161/jaha.113.000472
35.Fogarassy G, Vathy-Fogarassy Á, Kenessey I, Kásler M, Forster T. Risk prediction model for long-term heart failure incidence after epirubicin chemotherapy for breast cancer - A real-world data-based, nationwide classification analysis. *Int J Cardiol* 2019;285:47-52. doi: 10.1016/j.ijcard.2019.03.013
36.Gómez-Vecino A, Corchado-Cobos R, Blanco-Gómez A, et al. Intermediate Molecular Phenotypes to Identify Genetic Markers of Anthracycline-Induced Cardiotoxicity Risk. *Cells* 2023;12 doi: 10.3390/cells12151956
37.Vulsteke C, Pfeil AM, Maggen C, et al. Clinical and genetic risk factors for epirubicin-induced cardiac toxicity in early breast cancer patients. *Breast Cancer Res Treat* 2015;152:67-76. doi: 10.1007/s10549-015-3437-9
38.Güntürkün F, Akbilgic O, Davis RL, et al. Artificial Intelligence–Assisted Prediction of Late-Onset Cardiomyopathy Among Childhood Cancer Survivors. *JCO Clinical Cancer Informatics* 2021:459-468. doi: 10.1200/cci.20.00176
39.Jacobs JEJ, Guler I, Duchenne J, Janssens S, Van Aelst LNL. Predictability of cardiotoxicity: Experience of a Belgian cardio-oncology clinic. *International Journal of Cardiology* 2022;363:119-122. doi: 10.1016/j.ijcard.2022.06.063
40.Jones KA, Small AD, Ray S, et al. Radionuclide ventriculography phase analysis for risk stratification of patients undergoing cardiotoxic cancer therapy. *J Nucl Cardiol* 2022;29:581-589. doi: 10.1007/s12350-020-02277-z
41.Kang Y, Assuncao BL, Denduluri S, et al. Symptomatic Heart Failure in Acute Leukemia Patients Treated With Anthracyclines. *J Am Coll Cardiol CardioOnc* 2019;1:208-217. doi: 10.1016/j.jaccao.2019.10.008
42.Kotwinski P, Smith G, Cooper J, et al. Body Surface Area and Baseline Blood Pressure Predict Subclinical Anthracycline Cardiotoxicity in Women Treated for Early Breast Cancer. *PLoS One* 2016;11:e0165262. doi: 10.1371/journal.pone.0165262
43.Leerink JM, van der Pal HJH, Kremer LCM, et al. Refining the 10-Year Prediction of Left Ventricular Systolic Dysfunction in Long-Term Survivors of Childhood Cancer. *J Am Coll Cardiol CardioOnc* 2021;3:62-72. doi: 10.1016/j.jaccao.2020.11.013

44.Liu X, Tao L, Wang M, Li H, Xu W. ABSDELL Model: Development and Internal Validation of a Risk Prediction Model of LVEF Decline in Breast Cancer Patients Treated With Trastuzumab. *Clin Breast Cancer* 2023;23:23-31. doi: 10.1016/j.clbc.2022.10.010
45.Liu ZY, Wang YG, Huang XB, Qi XH, Qian CP, Zhao S. Development and Validation of a Diagnostic Nomogram to Predict the Anthracycline-Induced Early Cardiotoxicity in Children with Hematological Tumors. *Cardiovasc Toxicol* 2022;22:802-812. doi: 10.1007/s12012-022-09755-5
46.Moey MYY, Liles DK, Carabello BA. Concomitant use of renin-angiotensin-aldosterone system inhibitors prevent trastuzumab-induced cardiotoxicity in HER2+ breast cancer patients: an institutional retrospective study. *Cardiooncology* 2019;5:9. doi: 10.1186/s40959-019-0043-8
47.Oikonomou EK, Sangha V, Dhingra LS, et al. Artificial Intelligence-Enhanced Risk Stratification of Cancer Therapeutics-Related Cardiac Dysfunction Using Electrocardiographic Images. *Circ Cardiovasc Qual Outcomes* 2024;18:e011504. doi: doi:10.1161/CIRCOUTCOMES.124.011504
48.Otchere P, Adekoya O, Governor SB, et al. Development of cardiac risk prediction model in patients with HER-2 positive breast cancer on trastuzumab therapy. *Cardiooncology* 2023;9:26. doi: 10.1186/s40959-023-00177-y
49.Öztürk C, Validyev D, Becher UM, Weber M, Nickenig G, Tiyerili V. A novel scoring system to estimate chemotherapy-induced myocardial toxicity: Risk assessment prior to non-anthracycline chemotherapy regimens. *Int J Cardiol Heart Vasc* 2021;33:100751. doi: 10.1016/j.ijcha.2021.100751
50.Pohl J, Mincu R-I, Mrotzek SM, et al. ECG Scoring for the Evaluation of Therapy-Naïve Cancer Patients to Predict Cardiotoxicity. *Cancers* 2021;13:1197.
51.Rivero-Santana B, Saldaña-García J, Caro-Codón J, et al. Anthracycline-induced cardiovascular toxicity: validation of the Heart Failure Association and International Cardio-Oncology Society risk score. *Eur Heart J* 2024;46:273-284. doi: 10.1093/eurheartj/ehae496
52.Rushton M, Johnson C, Dent S. Trastuzumab-induced cardiotoxicity: testing a clinical risk score in a real-world cardio-oncology population. *Curr Oncol* 2017;24:176-180. doi: 10.3747/co.24.3349
53.Sun Y, Ping Y, Miao S, et al. Development of a multivariable clinical prediction model for liposomal doxorubicin-induced cardiotoxicity in adult breast cancer patients: a retrospective multicenter study. *Ann Transl Med* 2022;10:605. doi: 10.21037/atm-22-1935
54.Suntheralingam S, Fan C-PS, Calvillo-Argüelles O, Abdel-Qadir H, Amir E, Thavendiranathan P. Evaluation of Risk Prediction Models to Identify Cancer Therapeutics Related Cardiac Dysfunction in Women with HER2+ Breast Cancer. *J Clin Med* 2022;11:847.
55.Upshaw JN, Ruthazer R, Miller KD, et al. Personalized Decision Making in Early Stage Breast Cancer: Applying Clinical Prediction Models for Anthracycline Cardiotoxicity and Breast Cancer Mortality Demonstrates Substantial Heterogeneity of Benefit-Harm Trade-off. *Clin Breast Cancer* 2019;19:259-267.e251. doi: 10.1016/j.clbc.2019.04.012

56.Yagi R, Goto S, Himeno Y, et al. Artificial intelligence-enabled prediction of chemotherapy-induced cardiotoxicity from baseline electrocardiograms. *Nature Communications* 2024;15:2536. doi: 10.1038/s41467-024-45733-x
57.Yu AF, Lin IH, Jorgensen J, et al. Nomogram for Predicting Risk of Cancer Therapy-Related Cardiac Dysfunction in Patients With Human Epidermal Growth Factor Receptor 2-Positive Breast Cancer. *J Am Heart Assoc* 2023;12:e029465. doi: 10.1161/jaha.123.029465
58.Lyon AR, López-Fernández T, Couch LS, et al. 2022 ESC Guidelines on cardio-oncology developed in collaboration with the European Hematology Association (EHA), the European Society for Therapeutic Radiology and Oncology (ESTRO) and the International Cardio-Oncology Society (IC-OS). *Eur Heart J* 2022;43:4229-4361. doi: 10.1093/eurheartj/ehac244
59.Plana JC, Galderisi M, Barac A, et al. Expert consensus for multimodality imaging evaluation of adult patients during and after cancer therapy: a report from the American Society of Echocardiography and the European Association of Cardiovascular Imaging. *J Am Soc Echocardiogr* 2014;27:911-939. doi: 10.1016/j.echo.2014.07.012
60.Zamorano JL, Lancellotti P, Rodriguez Muñoz D, et al. 2016 ESC Position Paper on cancer treatments and cardiovascular toxicity developed under the auspices of the ESC Committee for Practice Guidelines: The Task Force for cancer treatments and cardiovascular toxicity of the European Society of Cardiology (ESC). *Eur Heart J* 2016;37:2768-2801. doi: 10.1093/eurheartj/ehw211
61.Alenezi A, McKiddie F, Nath M, Mayya A, Welch A. Cardiotoxicity detection tool for breast cancer chemotherapy: a retrospective study. *PeerJ Comput Sci* 2024;12:e2230. doi: 10.7717/peerj-cs.2230
62.Ali MT, Yucel E, Bouras S, et al. Myocardial Strain Is Associated with Adverse Clinical Cardiac Events in Patients Treated with Anthracyclines. *Journal of the American Society of Echocardiography* 2016;29:522-527.e523. doi: 10.1016/j.echo.2016.02.018
63.Araujo-Gutierrez R, Chitturi KR, Xu J, et al. Baseline global longitudinal strain predictive of anthracycline-induced cardiotoxicity. *Cardio-Oncology* 2021;7:4. doi: 10.1186/s40959-021-00090-2
64.Armenian SH, Ding Y, Mills G, et al. Genetic susceptibility to anthracycline-related congestive heart failure in survivors of haematopoietic cell transplantation. *British Journal of Haematology* 2013;163:205-213. doi: https://doi.org/10.1111/bjh.12516
65.Bergamini C, Niro L, Springhetti P, et al. Role of Early Left Atrial Functional Decline in Predicting Cardiotoxicity in HER2 Positive Breast Cancer Patients Treated With Trastuzumab. *Cardiovascular Toxicology* 2024;24:550-562. doi: 10.1007/s12012-024-09861-6
66.Bottinor WJ, Deng X, Bandyopadhyay D, et al. Myocardial Strain during Surveillance Screening Is Associated with Future Cardiac Dysfunction among Survivors of Childhood, Adolescent and Young Adult-Onset Cancer. *Cancers* 2023;15:2349.
67.Cai G, Li C, Li J, et al. Cardiac Substructures Dosimetric Predictors for Cardiac Toxicity After Definitive Radiotherapy in Esophageal Cancer. *International Journal of Radiation Oncology, Biology, Physics* 2023;115:366-381. doi: 10.1016/j.ijrobp.2022.08.013

68.Calvillo-Argüelles O, Thampinathan B, Somerset E, et al. Diagnostic and Prognostic Value of Myocardial Work Indices for Identification of Cancer Therapy–Related Cardiotoxicity. *JACC: Cardiovascular Imaging* 2022;15:1361-1376. doi: doi:10.1016/j.jcmg.2022.02.027
69.Choe JC, Choi JH, Choi JH, et al. Prolonged electromechanical delay as an early predictor of trastuzumab-induced cardiotoxicity in patients undergoing treatment for breast cancer. *Clinical Cardiology* 2018;41:1308-1314. doi: https://doi.org/10.1002/clc.23022
70.de Baat EC, Merkx R, Leerink JM, et al. Presence and utility of electrocardiographic abnormalities in long-term childhood cancer survivors. *Heart* 2024;110:726-734. doi: 10.1136/heartjnl-2023-323474
71.de Barros MVL, Macedo AVS, Sarvari SI, et al. Left Ventricular Regional Wall Motion Abnormality is a Strong Predictor of Cardiotoxicity in Breast Cancer Patients Undergoing Chemotherapy. *Arq Bras Cardiol* 2019;112:50-56. doi: DOI: 10.5935/abc.20180220
72.Demissei BG, Hubbard RA, Zhang L, et al. Changes in Cardiovascular Biomarkers With Breast Cancer Therapy and Associations With Cardiac Dysfunction. *Journal of the American Heart Association* 2020;9:e014708. doi: doi:10.1161/JAHA.119.014708
73.Demissei BG, Fan Y, Qian Y, et al. Left ventricular segmental strain and the prediction of cancer therapy-related cardiac dysfunction. *Eur Heart J Cardiovasc Imaging* 2021;22:418-426. doi: 10.1093/ehjci/jeaa288
74.Ehrhardt MJ, Liu Q, Mulrooney DA, et al. Improved Cardiomyopathy Risk Prediction Using Global Longitudinal Strain and N-Terminal-Pro-B-Type Natriuretic Peptide in Survivors of Childhood Cancer Exposed to Cardiotoxic Therapy. *Journal of Clinical Oncology* 2024;42:1265-1277. doi: 10.1200/jco.23.01796
75.Fawzy AA, El-Menyawi KA, Sallam WM, Zahran ME. Two-dimensional speckle tracking echocardiography in chemotherapy-induced cardiotoxicity in females with breast cancer. *Cardio-Oncology* 2024;10:13. doi: 10.1186/s40959-024-00209-1
76.Goldberg JF, Ness KK, Chi X, et al. Cardiovascular Family History Increases Risk for Late-Onset Adverse Cardiovascular Outcomes in Childhood Cancer Survivors: A St. Jude Lifetime Cohort Report. *Cancer Epidemiology, Biomarkers & Prevention* 2021;30:123-132. doi: 10.1158/1055-9965.Epi-20-0809
77.Hahn E, Jiang H, Ng A, et al. Late Cardiac Toxicity After Mediastinal Radiation Therapy for Hodgkin Lymphoma: Contributions of Coronary Artery and Whole Heart Dose-Volume Variables to Risk Prediction. *International Journal of Radiation Oncology, Biology, Physics* 2017;98:1116-1123. doi: 10.1016/j.ijrobp.2017.03.026
78.Hathaway QA, Abdeen Y, Conte J, et al. Prediction of heart failure and all-cause mortality using cardiac ultrasomics in patients with breast cancer. *The International Journal of Cardiovascular Imaging* 2024;40:1305-1317. doi: 10.1007/s10554-024-03101-2
79.Hochstadt A, Arnold J, Rosen R, et al. Longitudinal diastolic strain slope as an early sign for systolic dysfunction among patients with active cancer. *Clinical Research in Cardiology* 2021;110:569-578. doi: 10.1007/s00392-020-01776-w

80.Hou Y, Zhou Y, Hussain M, et al. Cardiac risk stratification in cancer patients: A longitudinal patient–patient network analysis. *PLOS Medicine* 2021;18:e1003736. doi: 10.1371/journal.pmed.1003736
81.Houbois CP, Nolan M, Somerset E, et al. Serial Cardiovascular Magnetic Resonance Strain Measurements to Identify Cardiotoxicity in Breast Cancer. *JACC: Cardiovascular Imaging* 2021;14:962. doi: doi:10.1016/j.jcmg.2020.09.039
82.Inoue K, Machino-Ohtsuka T, Nakazawa Y, et al. Early Detection and Prediction of Anthracycline-Induced Cardiotoxicity — A Prospective Cohort Study —. *Circ J* 2024;88:751-759. doi: 10.1253/circj.CJ-24-0065
83.Jacobs JEJ, Greason G, Mangold KE, et al. Artificial intelligence electrocardiogram as a novel screening tool to detect a newly abnormal left ventricular ejection fraction after anthracycline-based cancer therapy. *European Journal of Preventive Cardiology* 2023;31:560-566. doi: 10.1093/eurjpc/zwad348
84.Ladbury C, Li R, Danesharasteh A, et al. Explainable Artificial Intelligence to Identify Dosimetric Predictors of Toxicity in Patients with Locally Advanced Non-Small Cell Lung Cancer: A Secondary Analysis of RTOG 0617. *International Journal of Radiation Oncology, Biology, Physics* 2023;117:1287-1296. doi: 10.1016/j.ijrobp.2023.06.019
85.Leerink JM, Feijen EAM, Baat ECd, et al. A Biomarker-Based Diagnostic Model for Cardiac Dysfunction in Childhood Cancer Survivors. *JACC CardioOncol* 2024;6:236-247. doi: doi:10.1016/j.jaccao.2024.02.008
86.Leger KJ, Cushing-Haugen K, Hansen JA, et al. Clinical and Genetic Determinants of Cardiomyopathy Risk among Hematopoietic Cell Transplantation Survivors. *Biology of Blood and Marrow Transplantation* 2016;22:1094-1101. doi: 10.1016/j.bbmt.2016.02.017
87.Ma Y, Kang W, Bao Y, Jiao F, Ma Y. Clinical Significance of Ischemia-Modified Albumin in the Diagnosis of Doxorubicin-Induced Myocardial Injury in Breast Cancer Patients. *PLOS ONE* 2013;8:e79426. doi: 10.1371/journal.pone.0079426
88.Milks MW, Velez MR, Mehta N, et al. Usefulness of Integrating Heart Failure Risk Factors Into Impairment of Global Longitudinal Strain to Predict Anthracycline-Related Cardiac Dysfunction. *American Journal of Cardiology* 2018;121:867-873. doi: 10.1016/j.amjcard.2017.12.022
89.Mousavi N, Tan TC, Ali M, Halpern EF, Wang L, Scherrer-Crosbie M. Echocardiographic parameters of left ventricular size and function as predictors of symptomatic heart failure in patients with a left ventricular ejection fraction of 50–59% treated with anthracyclines. *European Heart Journal - Cardiovascular Imaging* 2015;16:977-984. doi: 10.1093/ehjci/jev113
90.Narayan HK, French B, Khan AM, et al. Noninvasive Measures of Ventricular-Arterial Coupling and Circumferential Strain Predict Cancer Therapeutics–Related Cardiac Dysfunction. *JACC: Cardiovascular Imaging* 2016;9:1131-1141. doi: doi:10.1016/j.jcmg.2015.11.024
91.Oikawa M, Yaegashi D, Yokokawa T, et al. D-Dimer Is a Predictive Factor of Cancer Therapeutics-Related Cardiac Dysfunction in Patients Treated With Cardiotoxic Chemotherapy. *Frontiers in Cardiovascular Medicine* 2022;Volume 8 - 2021 doi: 10.3389/fcvm.2021.807754

92.Posch F, Niedrist T, Glantschnig T, et al. Left ventricular ejection fraction and cardiac biomarkers for dynamic prediction of cardiotoxicity in early breast cancer. *Frontiers in Cardiovascular Medicine* 2022;Volume 9 - 2022 doi: 10.3389/fcvm.2022.933428
93.Terluk A, Stefani L, Boyd A, et al. Redefining anthracycline-related subclinical cardiotoxicity: ‘Absolute’ and ‘relative’ change in longitudinal strain. *ESC Heart Failure* 2024;11:3210-3221. doi: https://doi.org/10.1002/ehf2.14884
94.Tian C, Zhang H, Liu J, Xu M, Ma L. GDF-15 is a potential candidate biomarker for an elevated risk of cardiotoxicity in breast cancer patients receiving neoadjuvant dual anti-HER2 therapy. *Frontiers in Pharmacology* 2024;Volume 15 - 2024 doi: 10.3389/fphar.2024.1396133
95.Tjong MC, Bitterman DS, Brantley K, et al. Major adverse cardiac event risk prediction model incorporating baseline Cardiac disease, Hypertension, and Logarithmic Left anterior descending coronary artery radiation dose in lung cancer (CHyLL). *Radiother Oncol* 2022;169:105-113. doi: 10.1016/j.radonc.2022.02.010
96.Tlegenova Z, Balmagambetova S, Zholdin B, et al. Role of Clinical Risk Factors and B-Type Natriuretic Peptide in Assessing the Risk of Asymptomatic Cardiotoxicity in Breast Cancer Patients in Kazakhstan. *Diagnostics* 2023;13:3557.
97.Tu C, Shen H, Li X, et al. Longitudinal Evaluation of Coronary Arteries and Myocardium in Breast Cancer Using Coronary Computed Tomographic Angiography. *JACC Cardiovasc Imaging* 2024;17:1335-1347. doi: doi:10.1016/j.jcmg.2024.05.017
98.Usendia C, Shukla A, Kulkarni M, et al. Study of usefulness of speckle-tracking echocardiography in detecting left ventricular dysfunction among adult cancer patients undergoing chemotherapy. *Heart Vessels Transplant* 2023;7:282-289. doi: DOI: 10.24969/hvt.2023.401
99.Visscher H, Ross CJD, Rassekh SR, et al. Pharmacogenomic Prediction of Anthracycline-Induced Cardiotoxicity in Children. *Journal of Clinical Oncology* 2012;30:1422-1428. doi: 10.1200/jco.2010.34.3467
100.Visscher H, Rassekh SR, Sandor GS, et al. Genetic Variants in SLC22A17 and SLC22A7 are Associated With Anthracycline-Induced Cardiotoxicity in Children. *Pharmacogenomics* 2015;16:1065-1076. doi: 10.2217/pgs.15.61
101.Yaegashi D, Oikawa M, Yokokawa T, et al. Red Blood Cell Distribution Width Is a Predictive Factor of Anthracycline-Induced Cardiotoxicity. *Frontiers in Cardiovascular Medicine* 2020;Volume 7 - 2020 doi: 10.3389/fcvm.2020.594685
102.Zhou Y, Hou Y, Hussain M, et al. Machine Learning–Based Risk Assessment for Cancer Therapy–Related Cardiac Dysfunction in 4300 Longitudinal Oncology Patients. *Journal of the American Heart Association* 2020;9:e019628. doi: doi:10.1161/JAHA.120.019628
103.Sangha V, Nargesi AA, Dhingra LS, et al. Detection of Left Ventricular Systolic Dysfunction From Electrocardiographic Images. *Circulation* 2023;148:765-777. doi: 10.1161/circulationaha.122.062646
104.Chatterjee NA, Tikkanen JT, Panicker GK, et al. Simple electrocardiographic measures improve sudden arrhythmic death prediction in coronary disease. *Eur Heart J* 2020;41:1988-1999. doi: 10.1093/eurheartj/ehaa177

105.Wilson PW, D'Agostino RB, Levy D, Belanger AM, Silbershatz H, Kannel WB. Prediction of coronary heart disease using risk factor categories. *Circulation* 1998;97:1837-1847. doi: 10.1161/01.cir.97.18.1837
106.D'Agostino RB, Vasan RS, Pencina MJ, et al. General Cardiovascular Risk Profile for Use in Primary Care. *Circulation* 2008;117:743-753. doi: doi:10.1161/CIRCULATIONAHA.107.699579
107.Sorror ML, Maris MB, Storb R, et al. Hematopoietic cell transplantation (HCT)-specific comorbidity index: a new tool for risk assessment before allogeneic HCT. *Blood* 2005;106:2912-2919. doi: 10.1182/blood-2005-05-2004
108.Khan SS, Ning H, Shah SJ, et al. 10-Year Risk Equations for Incident Heart Failure in the General Population. *J Am Coll Cardiol* 2019;73:2388-2397. doi: https://doi.org/10.1016/j.jacc.2019.02.057
109.Hippisley-Cox J, Coupland C, Brindle P. Development and validation of QRISK3 risk prediction algorithms to estimate future risk of cardiovascular disease: prospective cohort study. *BMJ* 2017;357:j2099. doi: 10.1136/bmj.j2099
110.Conroy RM, Pyörälä K, Fitzgerald AP, et al. Estimation of ten-year risk of fatal cardiovascular disease in Europe: the SCORE project. *European Heart Journal* 2003;24:987-1003. doi: 10.1016/s0195-668x(03)00114-3
111.Hageman S, Pennells L, Ojeda F, Kaptoge S. SCORE2 risk prediction algorithms: new models to estimate 10-year risk of cardiovascular disease in Europe. *European Heart Journal* 2021;42:2439-2454. doi: 10.1093/eurheartj/ehab309
112.De Vries TI, Cooney MT, M. SR, Hageman S. SCORE2-OP risk prediction algorithms: estimating incident cardiovascular event risk in older persons in four geographical risk regions. *European Heart Journal* 2021;42:2455-2467. doi: 10.1093/eurheartj/ehab312
113.Armenian SH, Lacchetti C, Barac A, et al. Prevention and Monitoring of Cardiac Dysfunction in Survivors of Adult Cancers: American Society of Clinical Oncology Clinical Practice Guideline. *J Clin Oncol* 2017;35:893-911. doi: 10.1200/jco.2016.70.5400
114.Lyon AR, Dent S, Stanway S, et al. Baseline cardiovascular risk assessment in cancer patients scheduled to receive cardiotoxic cancer therapies: a position statement and new risk assessment tools from the Cardio-Oncology Study Group of the Heart Failure Association of the European Society of Cardiology in collaboration with the International Cardio-Oncology Society. *Eur J Heart Fail* 2020;22:1945-1960. doi: 10.1002/ejhf.1920
115.Herrmann J, Lerman A, Sandhu NP, Villarraga HR, Mulvagh SL, Kohli M. Evaluation and management of patients with heart disease and cancer: cardio-oncology. *Mayo Clin Proc* 2014;89:1287-1306. doi: 10.1016/j.mayocp.2014.05.013
